# Modulation of a Supramolecular Figure‐of‐Eight Strip Based on a Photoswitchable Stiff‐Stilbene

**DOI:** 10.1002/chem.202002051

**Published:** 2020-06-03

**Authors:** Romain Costil, Stefano Crespi, Lukas Pfeifer, Ben L. Feringa

**Affiliations:** ^1^ Stratingh Institute for Chemistry Zernike Institute for Advanced Materials University of Groningen Nijenborgh 4 9747 AG Groningen The Netherlands

**Keywords:** coordination-induced assembly, figure-of-eight, metallo-supramolecular complex, photoswitch, 3D architectures

## Abstract

The preparation, assembly and dynamic properties of photoswitchable bisphosphine ligands based on the stiff‐stilbene scaffold are reported. Directional bonding and coordination‐induced assembly allow complexation of these ligands with palladium(II), resulting in the formation of discrete metallo‐supramolecular entities. While the *Z* isomer forms a simple bidentate metallo‐macrocycle, an intricate double helicate figure‐of‐eight dimer is observed with the *E* ligand. Topologically 3D complexes can thus be obtained from 2D ligands. Upon irradiation with UV light, isomerization of the ligands allows control of the architecture of the formed complexes, resulting in a light‐triggered modulation of the supramolecular topology. Furthermore, a mechanistic investigation unveiled the dynamic nature of the helicate chirality, where a transmission of motion from the palladium centers yields an „eight‐to‐eight“ inversion.

## Graphical abstract

**Self‐assembly** of a metallo‐supramolecular figure‐of‐eight double helicate is presented. Using photoswitchable bisphosphine ligands as a rigid scaffold, the topology of the complex can be controlled. 3D architectures can thus be obtained from planar ligands. The dynamic chirality of the figure‐of‐eight strip results from transmission of motion from the metal centers (see figure).

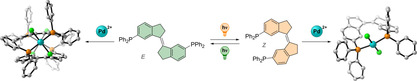

Molecular structures with a complex topology such as figure‐of‐eight strips have attracted attention not only because of the intrinsic aesthetic appeal,^1^ but also for their occurrence in natural compounds such as Lissoclinamide 7, a marine alkaloid with high cytotoxicity.^2^ Furthermore, this structural motif was observed in the recombinant structure of circular DNA.^3^ Moving away from the toolbox of biogenic molecules allows for more adaptability in the design of synthetic mimics to create artificial systems following a minimalistic approach compared to complex bio‐macromolecules.^4^ Various strategies have been introduced to engineer systems adopting this conformation.^5^ These include templating flexible macromolecules with metals^6^, ^7^ and organic effectors,^8^, ^9^ or using a rigid core to provide helical chirality^10^ and induce a twisted conformation.^11^, ^12^ This topology can be elusive^13^ or persistent,^14^ depending on the strategy.

Chemists have designed a variety of responsive metallo‐supramolecular systems triggered by various external and reversible stimuli such as light, pH, redox or temperature, to modulate the properties of complex systems.^15^ Amongst these, light is an ideal trigger due to its high spatio‐temporal resolution and tunability.^16^ A few examples allowing the control of topology,^17^ catalytic activity,^18^, ^19^ material properties,^20^, ^21^ or biological activity^22^ have been reported.

Recently, Sauvage and co‐workers reported the assembly of a flexible macrocycle into a metallo‐supramolecular figure‐of‐eight motif by binding to copper,^6^ while the work of Anderson et al. focused on the generation of this topology using organic molecules.^8^ However, the control of structural information in supramolecular entities using external actuators in combination with such intricate topologies is still rare.^23^ Typically, rigid ligands with well‐defined angles between the complexing moieties lead to highly defined structures such as pores^17^ or cages.^24^, ^25^ Alternatively, a more flexible design of the backbone can increase the supramolecular complexity for example, extended (double‐) helical structures.^1^, ^26^, ^27^ We were interested in designing minimalistic ligands for the photoaddressable self‐assembly of complexes with such chiral three‐dimensional topology. Herein, we report the forging of intricate chiral assemblies from rigid, structurally simple yet photoresponsive bisphosphine ligands.

Photochemical switches based on overcrowded alkenes^28^ such as stiff‐stilbenes offer opportunities as templates for supramolecular assemblies (Figure 1 a).^29^, ^30^ The large geometrical change induced upon isomerization—with dihedral angles of ca. 0° and 180° for the *Z* and *E* isomers, respectively—yields drastic differences between the molecular architectures.^17^ Furthermore, these rigid ligands with encoded directionality are ideal for coordination‐driven self‐assembly using directional bonding while maintaining a responsive behavior, as proposed by Stang and co‐workers.^31^ This approach has been explored to create intricate, polymeric^17^ metallo‐supramolecular assemblies and generates complexity from simple molecules in adaptive systems.^32^

**Figure 1:**
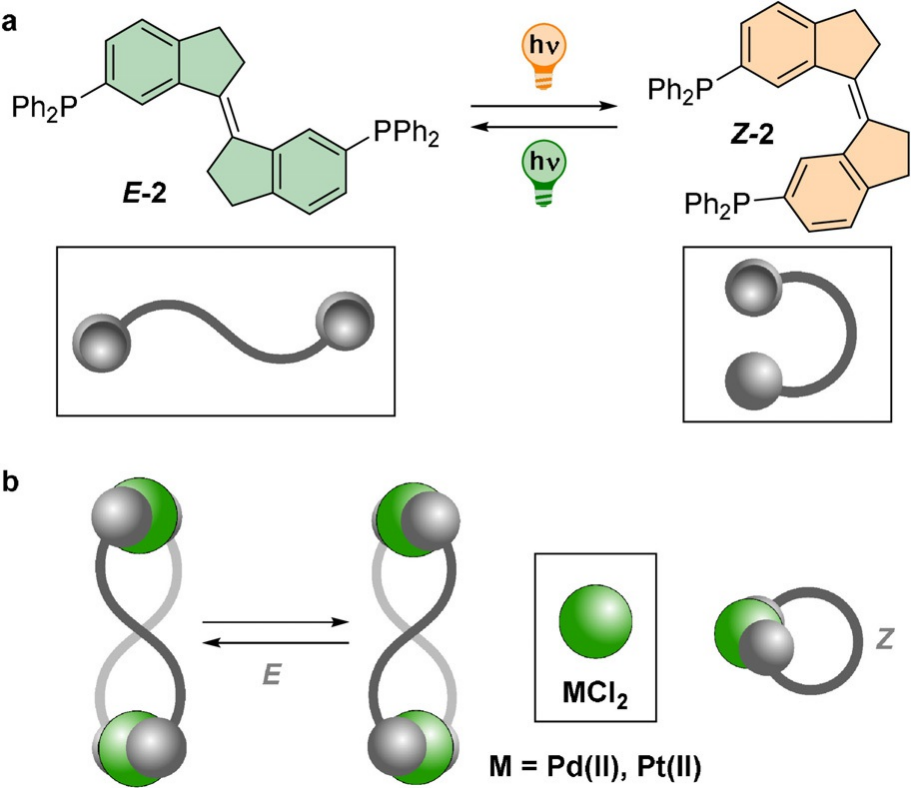
a) Photoswitchable ligand **2**, and b) its metallo‐supramolecular complexes.

We envisioned that using an easily accessible stiff‐stilbene skeleton would enable the preparation of self‐assembled, neutral palladium complexes with topologically complex architectures (Figure 1 b). The rigidity of the ligand, combined with the moderate energy of the coordination bond, facilitates the generation of discrete metallo‐macrocyclic structures, including a figure‐of‐eight strip, using simple scaffolds. Exploiting their intrinsic responsive nature, switching between these ligands was observed upon light irradiation, allowing the reversible control of chiral architectures.

Bisphosphine ligands ***Z*** **‐2** and ***E*** **‐2** were prepared in two steps from the corresponding dibromides^33^ (Scheme 1), which were readily converted to ***Z*** **‐1**/***E*** **‐1** in an aromatic Finkelstein reaction following a procedure by Buchwald.^34^ The resulting iodides proved suitable for phosphination. While different conditions were needed to provide each isomer, ***Z*** **‐2** and ***E*** **‐2** were obtained in moderate to good yields (see Supporting Information).

**Scheme 1:**
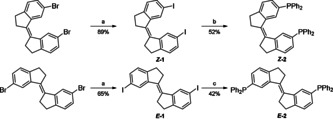
Synthesis of the bisphosphine ligands. a) CuI (15 mol %), DMEDA (30 mol %), NaI (6.0 equiv), dioxane (0.5 m), 130 °C, 24–48 h. b) HPPh_2_ (3.0 equiv), Pd(PPh_3_)_4_ (5 mol %), Et_3_N (4.0 equiv), toluene (0.1 m), 100 °C, 24 h. c) HPPh_2_ (2.2 equiv), Pd(OAc)_2_ (5 mol %), KOAc (2.2 equiv), DMAc (0.1 m), 120 °C, 3 h.

The electron density of the isomeric phosphines was compared via the corresponding selenide, prepared by refluxing the phosphine compounds with an excess of selenium in chloroform (see Supporting Information), and analyzed using ^31^P NMR. The resulting phosphorous signal appeared at 32.5 ppm and 33.0 ppm for the *Z* and *E* compounds, respectively. The ^1^ *J*(^77^Se‐^31^P) spin‐spin coupling was found to be equal in both isomers (364 Hz), suggesting similar donor properties of the phosphine lone pair of both ligands ***Z*** **‐2** and ***E*** **‐2**.^35^

The photoswitching of the isolated bisphosphines was studied in N_2_‐purged benzene (or [D_6_]benzene) solutions through UV‐vis and NMR spectroscopy. The absorption spectrum of ***Z*** **‐2** (*λ*_max_=356 nm) showed a distinct bathochromic shift compared to that of ***E*** **‐2** (*λ*_max_=338 and 356 nm), hence wavelengths of 365 and 385 nm were used to induce the *E→Z* and *Z→E* isomerization, respectively. In both cases, the photostationary states were reached by prolonging the irradiation until no further spectral changes were observed. Consequently, the *E*‐isomer was converted into the *Z* with a 365 nm LED at 20 °C resulting in the decrease of the absorption bands with maxima at 338 and 356 nm. The photostationary distribution associated to this conversion was 54:46 *E*:*Z* (Figure 2 a). Irradiating the sample at shorter wavelengths did not improve the photostationary state (PSS). On the other hand, the *Z* isomer was converted quantitatively into the *E* form via irradiation with *λ*=385 nm light, restoring the 338 and 356 nm absorption bands (Figure 2 b). The presence of isosbestic points corroborates the unimolecular nature of the photochemical transition, while the possibility to cycle many times between the two irradiation wavelengths provided evidence of the stability of the photoswitch (Figure 2 a).

**Figure 2:**
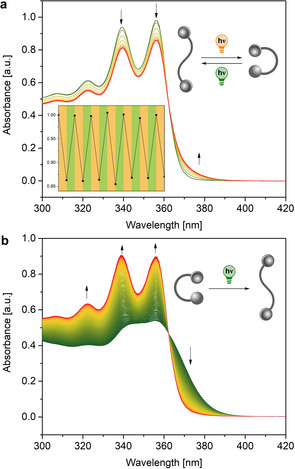
Photochemical switching of the bisphosphine ligands ***E*** **‐2** and ***Z***‐**2**. a) *E*→*Z* isomerization (365 nm irradiation). In the inset, the fatigue cycles for the *E*→*Z* (365 nm irradiation) and *Z*→*E* (385 nm irradiation) observed at 356 nm is shown. b) *Z*→*E* isomerization (385 nm irradiation).

Upon complexation of each bisphosphine ligand **2** with Pd(CH_3_CN)_2_Cl_2_ in toluene at room temperature, a single product was observed by ^1^H and ^31^P NMR. ***Z*** **‐2** formed the symmetrical complex ***Z*** **‐3** in excellent yield (Figure 3, see Supporting Information). In [D_6_]benzene, a deshielded aromatic signal at 10.41 ppm appeared as a triplet, suggesting the presence of virtual coupling by complexation of palladium in a *trans* fashion.^36^ *Trans* complexation with palladium was also supported by the downfield shift of the phosphine signal by ^31^P NMR at 20.6 ppm.^37^ Diffusion Ordered Spectroscopy (DOSY) NMR confirmed the presence of a single compound with a diffusion coefficient of 5.50 10^−6^ cm^2^ s^−1^ in [D_6_]benzene. This corresponds to a radius of about 6.68 Å, in line with the formation of a monomeric bidentate palladium complex. Finally, a single species was observed by Electrospray Ionisation Mass Spectroscopy (ESI‐MS) for the [***Z*** **‐3**‐Cl]^+^ ion. Treatment of ***Z*** **‐2** with one equivalent of K_2_PtCl_4_ in a mixture of benzene, ethanol and water formed a similar species (see Supporting Information), as confirmed by ^1^H, ^31^P NMR, as well as ESI‐MS. Presumably, ***Z*** **‐2** chelates Pt^II^ in a *trans*‐spanning bidentate complex in a similar fashion to Pd^II^.

**Figure 3:**
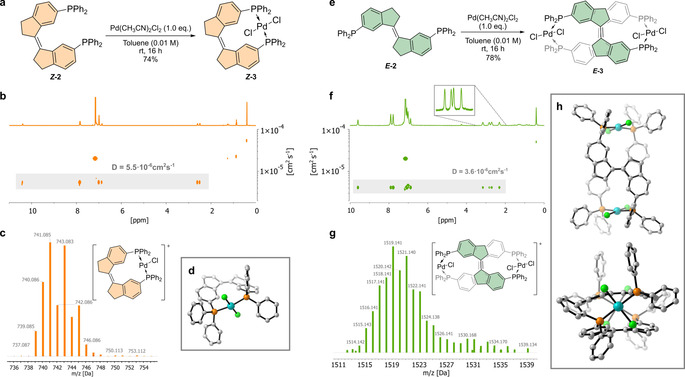
Synthesis and characterization of the palladium complexes. a) Synthesis of ***Z*** **‐3**. b) DOSY NMR analysis of ***Z*** **‐3**. c) ESI‐MS of the [***Z*** **‐3**‐Cl]^+^ ion of ***Z*** **‐3**. d) Single crystal X‐ray diffraction structure of ***Z*** **‐3** (protons and solvent omitted for clarity). e) Synthesis of ***E*** **‐3**. f) DOSY NMR analysis of ***E*** **‐3**. Inset shows diastereotopic C*H_A_H_B_* protons. g) ESI‐MS of the [***E*** **‐3**‐Cl]^+^ ion of ***E*** **‐3**. h) Single crystal X‐ray diffraction structure of ***E*** **‐3** (protons and solvent omitted for clarity).

Single crystals suitable for X‐ray diffraction were grown from a saturated solution of ***Z*** **‐3** in CDCl_3_. The structure obtained confirmed the *trans* arrangement of the phosphine atoms, with the chloride atoms pointing perpendicularly to the ligand's plane (Figure 3 d). The palladium atom was found to be slightly out of planarity (∠PPdP′=164.8°) in order to arrange for chelation, while the ligand adopts a skewed conformation, with a dihedral angle of 29.4° between the two phosphines.

Interestingly, the ^1^H NMR spectrum of the only product of the reaction of ***E*** **‐2** and Pd(CH_3_CN)_2_Cl_2_ included diastereotopic signals in the CH_2_ region, while the ^31^P NMR showed only one phosphine signal at around 21.8 ppm (Figure 3). This, together with the highly deshielded ^1^H triplet at 9.61 ppm, indicated the formation of an alternative complex ***E*** **‐3**. While the ^31^P NMR suggested the formation of a discrete metallo‐supramolecular entity, the ^1^H NMR pointed towards a chiral complex. ESI‐MS of the product revealed the presence of a single ion of a mass corresponding to [***E*** **‐3**‐Cl]^+^. Similarly, DOSY NMR confirmed the presence of a single species with a diffusion coefficient of 3.56 10^−6^ cm^2^ s^−1^ in [D_6_]benzene, supporting the formation of a dimer. Remarkably, while the group of Stang and co‐workers described the formation of metallo‐supramolecular polymers using stiff stilbene incorporating pyridine ligands,^17^ we only observed the discrete dimeric species ***E*** **‐3** with the phosphorus‐based system. Ligand ***E*** **‐2** also formed dimeric complexes when reacted with platinum(II) (see Supporting Information). However, due to the preference of platinum for *cis*‐chelation, a conformationally heterogenous mixture of dimers was observed by ^31^P NMR (see the Supporting Information). This configurational inhomogeneity prevented further analysis (vide infra).

Calculation of the structure of ***E*** **‐3** by DFT (ωB97X‐D/def2‐TZVP(def2‐TZVPP,SDD)//M06‐L/6‐31G*(LANL2DZ)) confirmed the existence of a dimer in which each palladium center binds with one phosphine atom of each ligand in a *trans* fashion (see the Supporting Information). The stiff‐stilbenes were found to lie antiparallel to one another, generating a bis‐helical structure. The same structure was found by X‐ray diffraction of crystals of ***E*** **‐3** grown by vapor diffusion of diisopropyl ether into a saturated solution of this compound in tetrahydrofuran (Figure 3 h). In this structure, the bisphosphine ligands are slightly twisted out of planarity, with a dihedral angle of 175.3° around the double bond. The figure‐of‐eight motif was thus confirmed by the presence of a tightly packed double helicate, connected at each extremity by coordination with a palladium atom.^38^ The increase of three‐dimensionality upon complexation was demonstrated by analysis of the Potential Moment of Inertia of ***Z*** **‐2** and ***E*** **‐2** compared to **Z‐3** and ***E*** **‐3** using LLAMA (see the Supporting Information).^39^

The conformational dynamics of complex ***E*** **‐3** were then investigated by Chiral Stationary Phase HPLC. Two distinct peaks were observed (Figure 4 a). Thorough analysis of the chromatogram at 25 °C revealed that full resolution could not be achieved as a plateau was observed. This suggested the dynamic nature of the system, that is, enantiomeric interconversion of the double helicate. Dynamic HPLC was used to probe the kinetic profile of this interconversion.^40^ The column temperature was adjusted to control on‐column interconversion from 22 to 37 °C. Retention times were kept as low as possible to prevent interaction with the stationary phase to have an impact on the barrier to interconversion.^41^ When raising the temperature, the height of the plateau was found to increase, a typical characteristic of chiral compounds racemizing within minutes at room temperature.^42^ Using the unified equation developed by Trapp,^42^ the kinetics of enantiomerization could be calculated by Eyring analysis.

**Figure 4:**
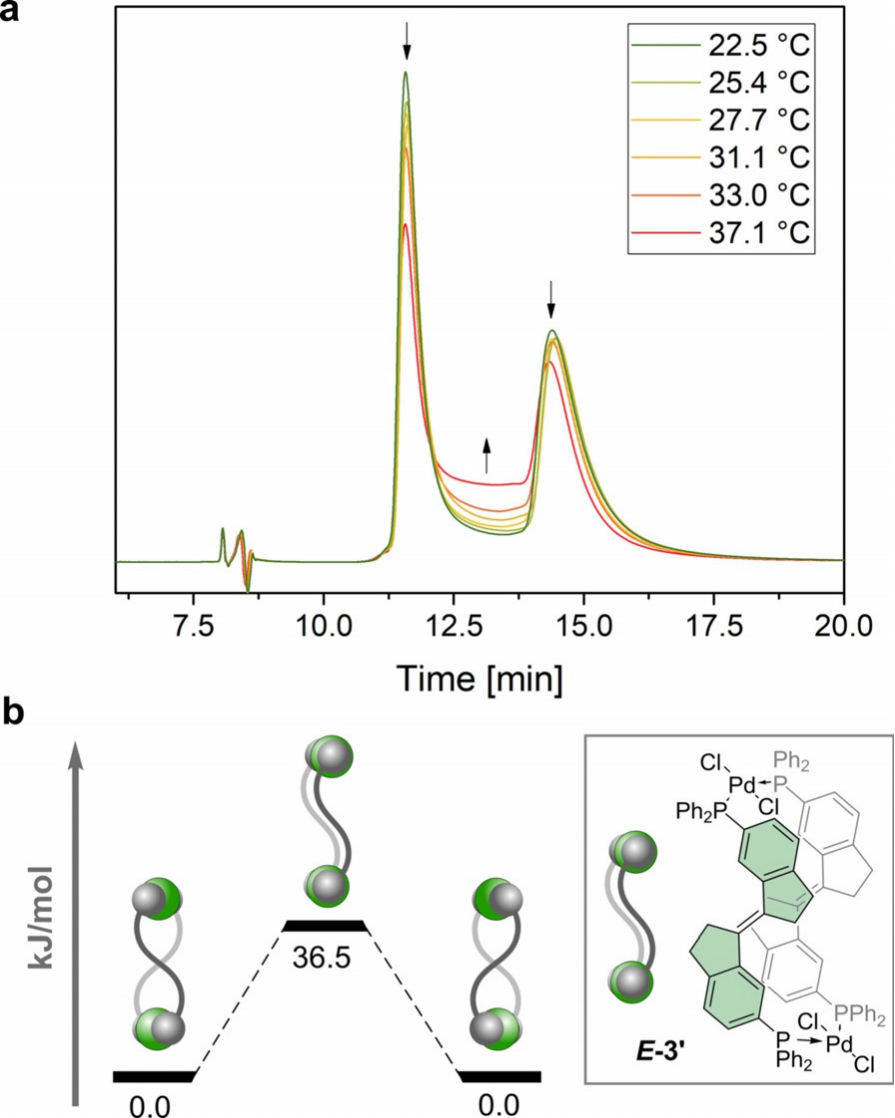
a) Part of the variable‐temperature HPLC chromatogram of ***E*** **‐3** on a CHIRALPAK ID column eluting with 35 % CH_2_Cl_2_ in heptane. b) Energy profile of the enantiomerization of ***E*** **‐3** via the population of ***E*** **‐3′** (ωB97X‐D/def2‐TZVP(def2‐TZVPP,SDD)//M06‐L/6‐31G*(LANL2DZ)).

Complex ***E*** **‐3** readily interconverts at 25 °C with an *on‐column* barrier to racemization Δ*G* ^≠^_293 K_=92.7±0.6 kJ mol^−1^, corresponding to a half‐life of racemization of ca. 32 min at 20 °C. The enthalpy of activation was found to be Δ*H* ^≠^=46.9 kJ mol^−1^ with an entropy value Δ*S* ^≠^=−155.5 J mol^−1^ K^−1^. The negative entropic factor suggests the absence of a dissociative mechanism. Racemization therefore likely occur by gear slippage via a highly symmetrical transition state where the palladium centers serve as midpoints of molecular motion.^43^ This reaction plausibly populates the *meso* form intermediate ***E*** **‐3′** which could not be observed due to its intrinsic instability compared to ***E*** **‐3** (calculated Δ*G* ^≠^_293 K_=36.5 kJ mol^−1^, see Figure 4 b). The enantiomers of the corresponding dimers, formed with platinum instead of palladium, could not be separated under similar conditions even at 0 °C (See Supporting Information). The stronger Pt−P bond, together with the larger Van der Waals radius of platinum vs. palladium and the seemingly lower interconversion barrier of the Pt^II^ dimers suggests indeed that enantiomerization of ***E*** **‐3** occurs through an associative mechanism.

In conclusion, we prepared and characterized photoswitchable bisphosphine ligands based on a stiff‐stilbene scaffold. Complexation of each isomer with palladium(II) resulted in the selective formation of discrete palladamacrocycles. While the *Z* ligand complexed in a bidentate fashion, the *E* isomer formed a dimeric species. Interestingly, the rigidity and directionality of this compound forced a topologically complex figure‐of‐eight strip, as demonstrated by DOSY NMR, mass spectrometry and X‐ray diffraction. Both enantiomers of this supramolecule were observed by CSP‐HPLC. Enantiomeric interconversion readily occurred at room temperature as demonstrated by on‐column helix inversion. Most likely, enantiomerization happens through a rigid transition state produced via gear slippage where the palladium(II) centers act as transmitters of molecular motion. This study demonstrates that the degree of three‐dimensionality of higher‐order structures obtained by coordination‐driven self‐assembly can be controlled by isomerization of simple, rod‐like planar ligands featuring directional bonding.

## Conflict of interest

The authors declare no conflict of interest.

## Supporting information

As a service to our authors and readers, this journal provides supporting information supplied by the authors. Such materials are peer reviewed and may be re‐organized for online delivery, but are not copy‐edited or typeset. Technical support issues arising from supporting information (other than missing files) should be addressed to the authors.

Supplementary
