# Supplementary material for: Modulation of a Supramolecular Figure‐of‐Eight Strip Based on a Photoswitchable Stiff‐Stilbene
Source: Chemistry. 2020 Jun 3;26(35):7783–7. doi: 10.1002/chem.202002051 (PMC7384132; doi:10.1002/chem.202002051)
Supplement: Supplementary file 1 — Supplementary [file CHEM-26-7783-s001.zip › z002051.pdf]

Table of Contents

Experimental Section 2

General methods and materials 2

Synthesis and characterization 3

UV-Vis spectra 7

NMR spectra 9

Variable-Temperature HPLC 30

Photochemical isomerization 32

Potential Moment of Inertia (PMI) plot 35

X-Ray structures 36

Computational analysis 40

References 48

Experimental Section

General methods and materials

Toluene was dried by using an MBRAUN solvent purification system (MBSPS-800). Dry DMAc and dioxane were purchased from Acros Organics and were stored under N_2. The degassing of solvents was carried out by sparging with N_2 for 15 min. All other chemicals were commercial products and were used as received. Flash chromatography (FC) was performed on a Büchi Reveleris purification system with Büchi cartridges, and thin-layer chromatography (TLC) was carried out on aluminum sheets coated with silica 60 F_{254} obtained from Merck (compounds were visualized with a UV lamp (254 nm)). ^1H and ^{13}C spectra were recorded on Varian Mercury-Plus 400 or on Varian Unity Inova 600 spectrometers at 298 K. Chemical shifts (δ) are denoted in parts per million (ppm) relative to $CDCl_3$ or C_6D_6 (for ^1H detection, δ = 7.26 ppm ($CDCl_3$) or 7.16 ppm (C_6D_6); for ^{13}C detection, δ = 77.16 ppm ($CDCl_3$) or 128.06 ppm (C_6D_6)). The splitting pattern of peaks is designated as follows: s (singlet), d (doublet), t (triplet), m (multiplet), br (broad), or dd (doublet of doublets). High-resolution mass spectrometry (ESI-MS) was performed on an LTQ Orbitrap XL spectrometer with ESI ionization. UV-vis spectra were recorded on Specord S600 and Hewlett-Packard HP 8543 diode arrays, equipped with a Quantum Northwest Peltier controller, in a 0.1 cm quartz cuvette. Irradiation of UV-vis samples was carried out at 20 °C using a Thorlabs model M365F1 high-power LED (4.1 mW) and a Thorlabs model M385F1 high-power LED (10.7 mW) positioned at a distance of 1 cm. NMR irradiation studies were performed at 20 °C with a Thorlabs model M365FP1 high-power LED (15.5 mW) and a Thorlabs model M385FP1 high power LED (23.2 mW).

Single-crystals were mounted on a cryoloop and placed in the nitrogen stream (100 K) of a Bruker-AXS D8 Venture diffractometer. Data collection and processing was carried out using the Bruker APEX3 software suite.[1] A multi-scan absorption correction was applied, based on the intensities of symmetry-related reflections measured at different angular settings (*SADABS*).[1] The structure was solved either using *SHELXS*[2] (LP18006) or *SHELXT*[3] (18005, 18011). and refinement was performed using *SHELXL*.[2] The hydrogen atoms were generated by geometrical considerations, constrained by idealized geometries and allowed to ride on their carrier atoms with an isotropic displacement parameter related to the equivalent displacement parameter of their carrier atoms.

Synthesis and characterization

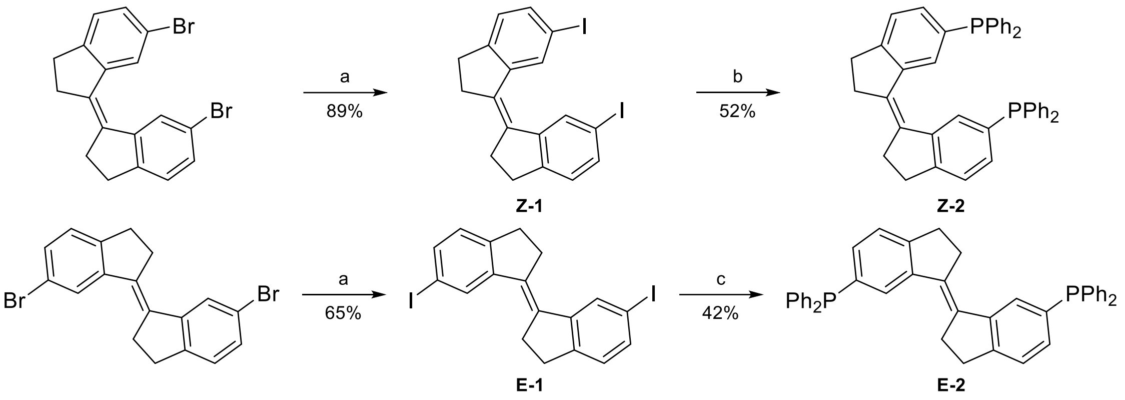

Scheme S1. Synthesis of the bisphosphine ligands. a) CuI (15 mol%), DMEDA (30 mol%), NaI (6.0 equiv.), dioxane (0.5 M), 130 °C, 24–48 h. b) $HPPh_2$ (3.0 equiv.), $Pd(PPh_3)_4$ (5 mol%), Et_3N (4.0 equiv.), toluene (0.1 M), 100 °C, 24 h. c) $HPPh_2$ (2.2 equiv.), $Pd(OAc)_2$ (5 mol%), KOAc (2.2 equiv.), DMAc (0.1 M), 120 °C, 3 h.

Z-6,6'-diiodo-2,2',3,3'-tetrahydro-1,1'-biindenylidene *Z*-1

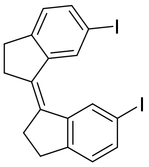

In a sealed tube, a suspension of (Z)-6,6'-dibromo-2,2',3,3'-tetrahydro-1,1'-biindenylidene[5] (450 mg, 1.15 mmol, 1.0 equiv.), NaI (1.04 g, 6.9 mmol, 6.0 equiv.), CuI (33 mg, 0.17 mmol, 0.15 equiv.) and DMEDA (37 µL, 0.35 mmol, 0.30 equiv.) in dioxane (2.3 mL, 0.5 M) was heated at 120 °C under a N_2 atmosphere for 24 h. The mixture was then treated with a saturated aqueous NH_4Cl solution, and extracted with $CHCl_3$. The subsequent organic layer was washed with brine, dried over $MgSO_4$, and volatiles were removed under vacuum. Purification by flash column chromatography (SiO_2, pentane) afforded the title compound as an off-white solid (0.50 g, 89%) with moderate purity. **^{1}H NMR (300 MHz, CDCl$_3$)** δ 8.38 (s, 2H, CH_{Ar}), 7.48 (d, J = 7.9 Hz, 2H, CH_{Ar}), 7.04 (d, J = 8.0 Hz, 2H, CH_{Ar}), 2.92 (t, J = 6.6 Hz, 4H, CH_2), 2.76 (t, J = 6.6 Hz, 4H, CH_2). **^{13}C NMR (101 MHz, CDCl$_3$)** δ 147.9 (C_{Ar}), 142.8 (C_{Ar}), 136.1 (C_{Ar}H), 135.2 (C_{Ar}), 132.4 (C_{Ar}H), 127.2 (C_{Ar}H), 90.9 (C_{Ar}I), 34.8 (CH$_2$), 30.4 (CH$_2$). **HRMS (APCI) m/z:** 484.9254 ([M+H]$^+$, calcd for $C_{18}H_{15}I_2^+$: 484.9258)

Z-6,6'-bis(diphenylphosphaneyl)-2,2',3,3'-tetrahydro-1,1'-biindenylidene *Z*-2

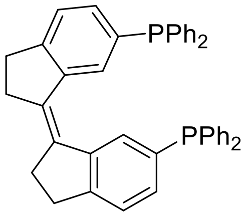

A suspension of ***Z*-1** (220 mg, 0.45 mmol, 1.0 equiv.), $HPPh_2$ (0.24 mL, 1.4 mmol, 3.0 equiv.), $Pd(PPh_3)_4$ (27 mg, 0.023 mmol, 5mol%), Et_3N (25 mL, 1.8 mmol, 4.0 equiv.) in toluene (4.5 mL, 0.1 M) was refluxed under a N_2 atmosphere for 24 h. The mixture was then filtered over Celite, washing with CH_2Cl_2. Volatiles were remove under vacuum, and the mixture was triturated in pentane, followed by flash column chromatography (SiO_2, pentane/CH_2Cl_2 95:5 to 60:40) to afford of the title compound as a white solid (140 mg, 52%). **^{1}H NMR (400 MHz, CDCl$_3$)** δ 8.21 (d, J = 10.3 Hz, 2H, CH_{Ar}), 7.35 – 7.22 (m, 20H, CH_{Ar}), 7.20 (d, J = 7.7 Hz, 2H, CH_{Ar}), 6.96 (dd, J = 7.5, 5.8 Hz, 2H, CH_{Ar}), 2.94 (t, J = 6.8 Hz, 4H, CH_2), 2.78 (t, J = 6.8 Hz, 4H, CH_2). **^{13}C NMR (101 MHz, CDCl$_3$)** δ 149.5 (C_{Ar}), 140.9 (d, J = 12.1 Hz, C_{Ar}), 138.1 (d, J = 12.5 Hz, C_{Ar}), 135.2 (C_{Ar}), 134.4 (d, J = 12.1 Hz, C_{Ar}), 133.8 (d, J = 19.5 Hz, CH$_{Ar}$), 132.8 (d, J = 8.9 Hz, CH$_{Ar}$), 129.7 (d, J = 35.6 Hz, CH$_{Ar}$), 128.5, 128.4 (d, J = 2.6 Hz, CH$_{Ar}$), 125.6 (d, J = 4.5 Hz, CH$_{Ar}$), 35.1 (CH$_2$), 30.7 (CH$_2$). **^{31}P NMR (162 MHz, CDCl$_3$)** δ -8.32. **HRMS (ESI) m/z:** 623.2103 ([M+Na]$^+$, calcd for $C_{42}H_{34}P_2Na^+$: 623.2033)

Z-(2,2',3,3'-tetrahydro-[1,1'-biindenylidene]-6,6'-diyl)bis(diphenylphosphine selenide) ***Z*-2Se**

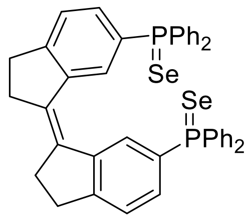

A suspension of ***Z*-2** (10 mg, 0.017 mmol, 1.0 equiv.) and Se (27 mg, 0.34 mmol, 2.0 equiv.) in $CHCl_3$ (0.8 mL, 0.02 M) was refluxed under a N_2 atmosphere for 4 h. The mixture was then filtered over Celite, washing with CH_2Cl_2. Volatiles were removed under vacuum to afford of the title compound as an off-white solid (12 mg, 93%). **^{1}H NMR (400 MHz, $CDCl_3$)** δ 8.63 (d, J = 15.2 Hz, 2H, CH_{Ar}), 7.71 (d, J = 7.7 Hz, 4H, CH_{Ar}), 7.67 (d, J = 7.8 Hz, 4H, CH_{Ar}), 7.43 – 7.36 (m, 14H, CH_{Ar}), 7.35 – 7.27 (m, 2H), 2.99 (t, J = 6.7 Hz, 4H, CH_2), 2.79 (t, J = 6.8 Hz, 4H, CH_2). **^{31}P NMR (162 MHz, $CDCl_3$)** δ 32.5 (d, J = 364 Hz). **HRMS (ESI) m/z:** 761.0530 ($[M+H]^+$, calcd for $C_{42}H_{35}P_2Se_2^+$: 761.0544).

E-6,6'-diiodo-2,2',3,3'-tetrahydro-1,1'-biindenylidene ***E*-1**

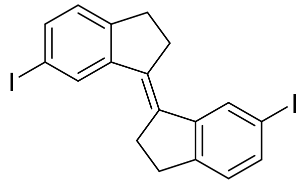

In a sealed tube, a suspension of (E)-6,6'-dibromo-2,2',3,3'-tetrahydro-1,1'-biindenylidene[5] (0.80 g, 2.1 mmol, 1.0 equiv.), NaI (1.84 g, 12.3 mmol, 6.0 equiv.), CuI (59 mg, 0.31 mmol, 0.15 equiv.) and DMEDA (66 µL, 0.62 mmol, 0.30 equiv.) in dioxane (4.1 mL, 0.5 M) was heated at 120 °C under a N_2 atmosphere for 48 h. The mixture was then treated with a saturated aqueous NH_4Cl solution, and extracted with $CHCl_3$. The subsequent organic layer was washed with brine, dried over $MgSO_4$, and volatiles were removed under vacuum. Trituration in toluene afforded the title compound as a beige solid (0.66 g, 65%). **^{1}H NMR (400 MHz, C_6D_6)** δ 7.87 (s, 2H, CH_{Ar}), 7.38 (d, J = 7.9 Hz, 2H, CH_{Ar}), 6.62 (d, J = 7.9 Hz, 2H, CH_{Ar}), 2.52 (d, J = 6.3 Hz, 4H, CH_2), 2.48 (d, J = 4.6 Hz, 4H, CH_2); too insoluble for a ^{13}C NMR and HRMS measurements.

E-6,6'-bis(diphenylphosphaneyl)-2,2',3,3'-tetrahydro-1,1'-biindenylidene ***E*-2**

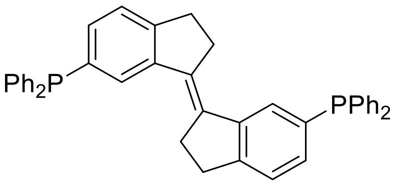

A suspension of ***E*-1** (532 mg, 1.1 mmol, 1.0 equiv.), $HPPh_2$ (0.42 mL, 2.4 mmol, 2.2 equiv.), $Pd(OAc)_2$ (12 mg, 0.055 mmol, 5mol%), KOAc (237 mg, 2.4 mmol, 2.2 equiv.) in DMAc (11.0 mL, 0.1 M) was heated to 120 °C under a N_2 atmosphere for 3 h. The mixture was then diluted in CH_2Cl_2, washed with water, brine, and dried over Na_2SO_4. Volatiles were remove under vacuum, and the mixture was triturated in pentane, followed by flash column chromatography (SiO_2, pentane/CH_2Cl_2 50:50 to 10:90) to afford of the title compound as a light yellow solid (277 mg, 42%). **^{1}H NMR (400 MHz, $CDCl_3$)** δ 7.46 (d, J = 8.3 Hz, 2H, CH_{Ar}), 7.34 (br. s, 18H, CH_{Ar}), 7.25 (t, J = 8.3 Hz, 3H, CH_{Ar}), 7.18 (t, J = 7.8 Hz, 3H, CH_{Ar}), 2.99 (t, J = 6.4 Hz, 4H, CH_2), 2.78 (t, J = 6.7 Hz, 4H, CH_2). **^{13}C NMR (101 MHz, $CDCl_3$)** δ 148.4 (C_{Ar}), 143.6 (d, J = 7.6 Hz, C_{Ar}), 137.5 (br. s, C_{Ar}), 135.5 (C_{Ar}), 133.8 (d, J = 18.8 Hz, CH_{Ar}), 132.9 (d, J = 20.6 Hz, CH_{Ar}), 130.1 (d, J = 20.1 Hz, CH_{Ar}), 128.8 (CH_{Ar}), 128.6 (d, J = 6.9 Hz, CH_{Ar}), 125.22 (d, J = 8.2 Hz, CH_{Ar}), 31.8 (CH_2), 31.0 (CH_2). **^{31}P NMR (162 MHz, $CDCl_3$)** δ -8.03. **HRMS (ESI) m/z:** 623.2104 ($[M+Na]^+$, calcd for $C_{42}H_{34}P_2Na^+$: 623.2033).

E-(2,2',3,3'-tetrahydro-[1,1'-biindenylidene]-6,6'-diyl)bis(diphenylphosphine selenide) ***E*-2Se**

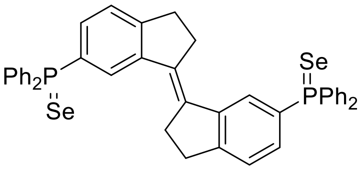

A suspension of ***E*-2** (10 mg, 0.017 mmol, 1.0 equiv.) and Se (27 mg, 0.34 mmol, 2.0 equiv.) in CHCl3 (0.8 mL, 0.02 M) was refluxed under a N_2 atmosphere for 4 h. The mixture was then filtered over Celite, washing with CH_2Cl_2. Volatiles were removed under vacuum to afford of the title compound as an off-white solid (13 mg, quant). **^{1}H NMR (400 MHz, $CDCl_3$)** δ 8.00 (d, J = 15.0 Hz, 2H, CH_{Ar}), 7.75 (d, J = 7.6 Hz, 4H, CH_{Ar}), 7.72 (d, J = 7.7 Hz, 4H, CH_{Ar}), 7.53 – 7.38 (m, 14H, CH_{Ar}), 7.32 (dd, J = 7.8, 3.0 Hz, 2H, CH_{Ar}), 3.07 (t, J = 6.6 Hz, 4H, CH_2), 2.93 (t, J = 6.5 Hz, 4H, CH_2). **^{31}P NMR (162 MHz, $CDCl_3$)** δ 33.0 (d, J = 364 Hz). **HRMS (ESI) m/z:** 761.0532 ($[M+H]^+$, calcd for $C_{42}H_{35}P_2Se_2^+$: 761.0544).

Z-6,6'-bis(diphenylphosphaneyl)-2,2',3,3'-tetrahydro-1,1'-biindenylidene palladium(II) dichloride ***Z*-3**

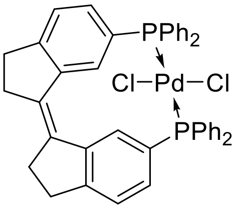

A solution of ***Z*-2** (60 mg, 0.1 mmol, 1.0 equiv.) and $PdCl_2(CH_3CN)_2$ (26 mg, 0.1 mmol, 1.0 equiv.) in toluene (10 mL, 0.01 M) was stirred at room temperature under a N_2 atmosphere for 16 h. The precipitate was then filtered and rinsed with pentane to afford of the title compound as a yellow solid (58 mg, 74%). **^{1}H NMR (400 MHz, $CDCl_3$)** δ 9.88 (t, J = 8.0 Hz, 2H, CH_{Ar}), 7.60 (q, J = 6.1 Hz, 8H, CH_{Ar}), 7.39 (q, J = 7.1 Hz, 4H, CH_{Ar}), 7.34 (t, J = 7.5 Hz, 8H, CH_{Ar}), 7.22 (d, J = 7.7 Hz, 2H, CH_{Ar}), 7.03 (dt, J = 8.1, 4.3 Hz, 2H, CH_2), 3.02 (t, J = 6.6 Hz, 2H), 2.90 (t, J = 6.6 Hz, 2H, CH_2). **^{13}C NMR (101 MHz, $CDCl_3$)** δ 150.9 (C_{Ar}), 140.9 (t, J = 7.8 Hz, C_{Ar}), 135.4 (C_{Ar}), 135.2 (t, J = 6.0 Hz, CH_{Ar}), 134.8 (t, J = 13.4 Hz, CH_{Ar}), 130.8 (t, J = 1.8 Hz, CH_{Ar}), 130.5 (CH_{Ar}), 129.1 (t, J = 24.3 Hz, C_{Ar}), 128.0 (t, J = 5.3 Hz, CH_{Ar}), 127.0 (t, J = 24.3 Hz, C_{Ar}), 125.2 (t, J = 4.5 Hz, CH_{Ar}), 34.1 (CH_2), 30.7 (CH_2). **^{31}P NMR (162 MHz, $CDCl_3$)** δ 20.6. **DOSY (600 MHz, C_6D_6)** D = $5.50.10^{-10}$ m^2 s^{-1}. **HRMS (ESI) m/z:** 741.085 ($[M-Cl]^+$, calcd for $C_{42}H_{34}ClP_2Pd^+$: 741.0859).

Z-6,6'-bis(diphenylphosphaneyl)-2,2',3,3'-tetrahydro-1,1'-biindenylidene platinum(II) dichloride ***Z*-3Pt**

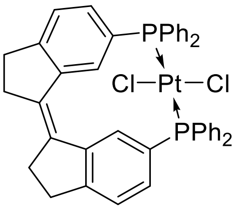

A solution of ***Z*-2** (30.0 mg, 0.05 mmol, 1.0 equiv.) in benzene (1.0 mL, 0.05 M) was added to a suspension of K_2PtCl_4 (20.7 mg, 0.05 mmol, 1.0 equiv.) in EtOH/H_2O (5:1, 1.2 mL, 0.04 M) and the mixture was stirred at room temperature for 72 h. Volatiles were then evaporated and the solid was triturated in water, followed by methanol to afford of the title compound as an off-white solid (26 mg, 60%). **^{1}H NMR (600 MHz, $CDCl_3$)** δ 9.88 (t, J = 7.8 Hz, 2H, CH_{Ar}), 7.65 – 7.51 (m, 8H, CH_{Ar}), 7.41 – 7.33 (m, 12H, CH_{Ar}), 7.22 (d, J = 7.7 Hz, 2H, CH_{Ar}), 7.07 (dt, J = 9.0, 4.4 Hz, 2H, CH_{Ar}), 3.04 (t, J = 6.9 Hz, 4H, CH_2), 2.90 (t, J = 6.9 Hz, 4H, CH_2). **^{13}C NMR (151 MHz, $CDCl_3$)** δ 150.8 (C_{Ar}), 140.5 (t, J = 7.4 Hz, C_{Ar}), 135.4 (C_{Ar}), 135.2 (t, J = 5.9 Hz, CH_{Ar}), 135.0 (t, J = 12.1 Hz, CH_{Ar}), 131.1 (t, J = 2.8 Hz, CH_{Ar}), 130.6 (CH_{Ar}), 128.2 (t, J = 28.1 Hz, C_{Ar}), 127.9 (t, J = 5.3 Hz, CH_{Ar}), 126.4 (t, J = 29.2 Hz, C_{Ar}), 124.9 (t, J = 4.8 Hz, CH_{Ar}), 34.0 (CH_2), 30.7 (CH_2). **^{31}P NMR (162 MHz, $CDCl_3$)** δ.19.3. **HRMS (ESI) m/z:** 830.1461 ($[M-Cl]^+$, calcd for $C_{42}H_{34}ClP_2Pt^+$: 830.1472).

E-3

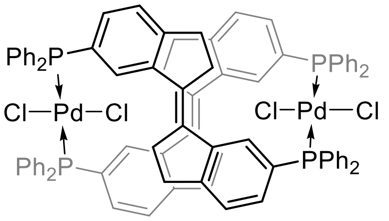

A solution of ***E*-2** (120 mg, 0.2 mmol, 1.0 equiv.) and $PdCl_2(CH_3CN)_2$ (52 mg, 0.2 mmol, 1.0 equiv.) in toluene (20 mL, 0.01 M) was stirred at room temperature under a N_2 atmosphere for 16 h. The volatiles were removed under vacuum, and the mixture was suspended in 4 mL of toluene. The precipitate was then filtered and rinsed with pentane to afford of the title compound as a yellow solid (121 mg, 79%). Note: analysis of this compound is reported $CDCl_3$ because of poor solubility in other solvents prevented ^{13}C NMR analysis. However, the title compound was found to exist as a mixture of *trans-trans* and *trans-cis* isomers at the palladium in a 6:1 ratio. Consequently, a ^{1}H NMR spectrum in C_6D_6 was also added to the spectra in the next Section. **^{1}H NMR (400 MHz, CDCl$_3$)** δ 9.21 (t, J = 7.5 Hz, 4H, C*H$_{Ar}$*), 7.62 (q, J = 6.5 Hz, 8H, C*H$_{Ar}$*), 7.55 (q, J = 5.9 Hz, 8H, C*H$_{Ar}$*), 7.46 – 7.32 (m, 24H, C*H$_{Ar}$*), 7.20 (d, J = 7.7 Hz, 4H, C*H$_{Ar}$*), 7.08 – 7.01 (m, 4H, C*H$_{Ar}$*), 2.96 (dd, J = 15.2, 9.5 Hz, 4H, C*H$_2$*), 2.90 – 2.80 (m, 4H, C*H$_2$*), 2.60 – 2.46 (m, 4H, C*H$_2$*), 2.23 (dd, J = 17.2, 9.3 Hz, 4H, C*H$_2$*). **^{13}C NMR (101 MHz, CDCl$_3$)** δ 151.3 (*C*$_{Ar}$), 144.0 (t, J = 7.2 Hz, *C*$_{Ar}$), 136.3 (*C*H$_{Ar}$), 135.6 (t, J = 5.3 Hz, *C*H$_{Ar}$), 135.3 (t, J = 12.6 Hz, *C*$_{Ar}$), 134.3 (t, J = 6.2 Hz, *C*H$_{Ar}$), 132.3 (t, J = 1.6 Hz, *C*$_{Ar}$), 130.4 (*C*H$_{Ar}$), 130.1 (*C*H$_{Ar}$), 129.8 (*C*H$_{Ar}$), 128.2 (t, J = 5.5 Hz, *C*H$_{Ar}$), 127.9 (*C*H$_{Ar}$), 127.6 (t, J = 5.0 Hz, *C*H$_{Ar}$), 124.9 (t, J = 4.4 Hz, *C*H$_{Ar}$), 32.5 (*C*H$_2$), 31.1 (*C*H$_2$). **^{31}P NMR (162 MHz, C$_6$D$_6$)** δ 21.5. **DOSY (600 MHz, C$_6$D$_6$)** D = 3.56 10^{-10} m^2 s^{-1}. **HRMS (ESI) m/z:** 1517.141 ([M-Cl]$^+$, calcd for $C_{84}H_{68}Cl_3P_4Pd_2^+$: 1517.1407).

E-3Pt

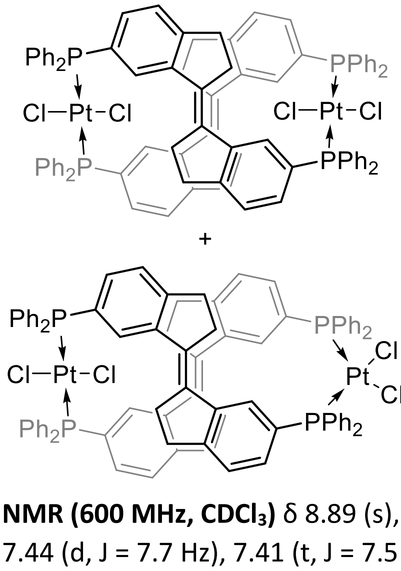

A solution of ***Z*-2** (30.0 mg, 0.05 mmol, 1.0 equiv.) in benzene (1.0 mL, 0.05 M) was added to a suspension of K_2PtCl_4 (20.7 mg, 0.05 mmol, 1.0 equiv.) in EtOH/H_2O (5:1, 1.2 mL, 0.04 M) and the mixture was stirred at room temperature for 72 h. Volatiles were then evaporated and the solid was triturated in water, followed by methanol to afford of the title compound as an off-white solid (23 mg, 53%). Note: analysis of this compound is reported $CDCl_3$ because of poor solubility in other solvents. However, the title compound was found to exist as a mixture of *trans-trans* and *trans-cis* isomers at the palladium in a 1:1.8 ratio (according to ^{31}P NMR shifts). Due to the substantial overlap in the ^{1}H NMR, the signals were not assigned. Similarly, ^{13}C NMR of this compound is not assigned but is reported in the next section. **^{1}H NMR (600 MHz, CDCl$_3$)** δ 8.89 (s), 8.03 (d, J = 7.6 Hz), 7.68 (d, J = 7.7 Hz), 7.64 (s), 7.49 (d, J = 7.6 Hz), 7.44 (d, J = 7.7 Hz), 7.41 (t, J = 7.5 Hz), 7.38 – 7.22 (m), 7.22 – 7.15 (m), 7.13 (t, J = 7.7 Hz), 7.03 (d, J = 7.8 Hz), 7.00 (d, J = 7.8 Hz), 6.92 (d, J = 7.8 Hz), 6.35 (s), 3.09 – 2.99 (m), 2.94 (dt, J = 17.1, 7.7 Hz), 2.89 – 2.74 (m), 2.75 – 2.63 (m, 2H), 2.63 – 2.45 (m), 2.34 (ddd, J = 17.6, 9.8, 2.8 Hz), 2.29 – 2.20 (m).. **^{31}P NMR (243 MHz, CDCl$_3$)** δ 21.4, 15.0, 14.5. **HRMS (ESI) m/z:** 1695.2647 ([M-Cl]$^+$, calcd for $C_{84}H_{68}Cl_3P_4Pt_2^+$: 1695.2632).

UV-Vis spectra

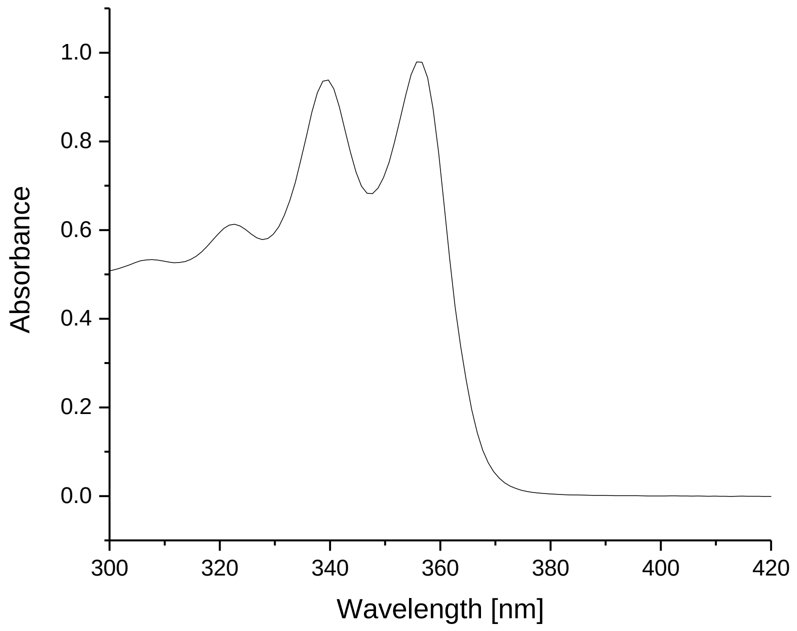

UV-Vis spectrum of *E*-2 in benzene (50 μM)

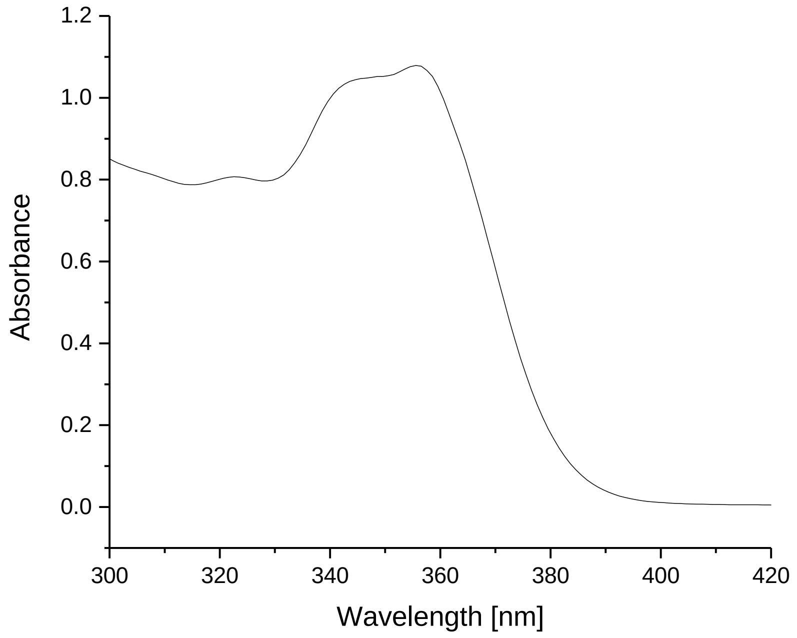

UV-Vis spectrum of *Z*-2 in benzene (50 μM)

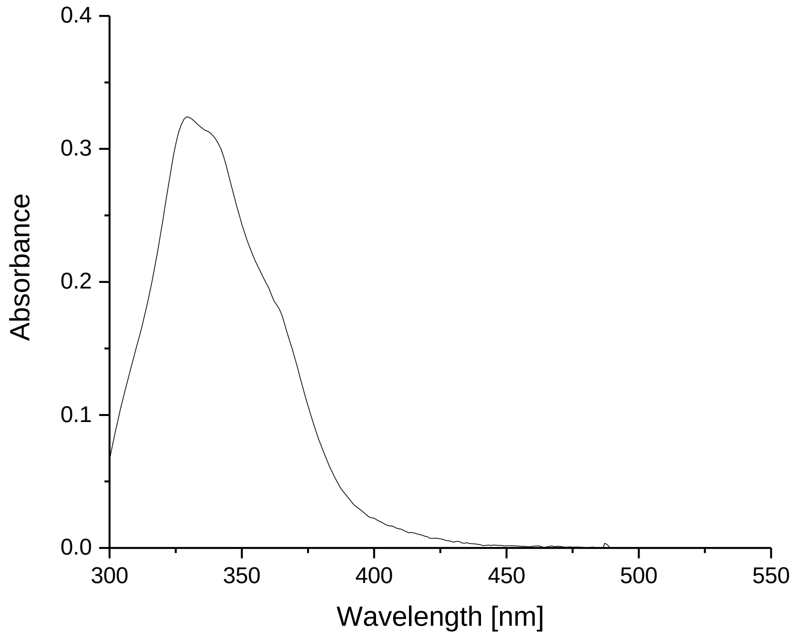

UV-Vis spectrum of *E*-3 in benzene (25 μM)

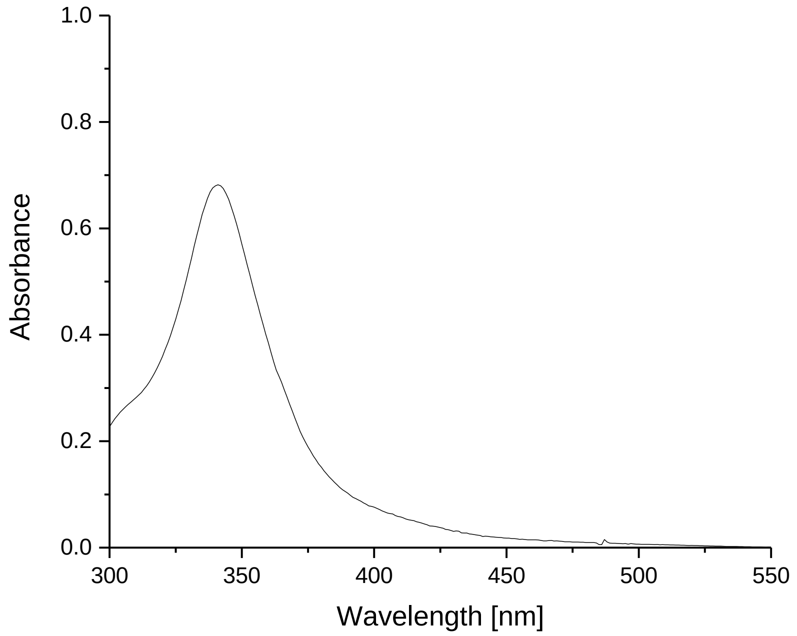

UV-Vis spectrum of *Z*-3 in benzene (25 μM)

NMR spectra

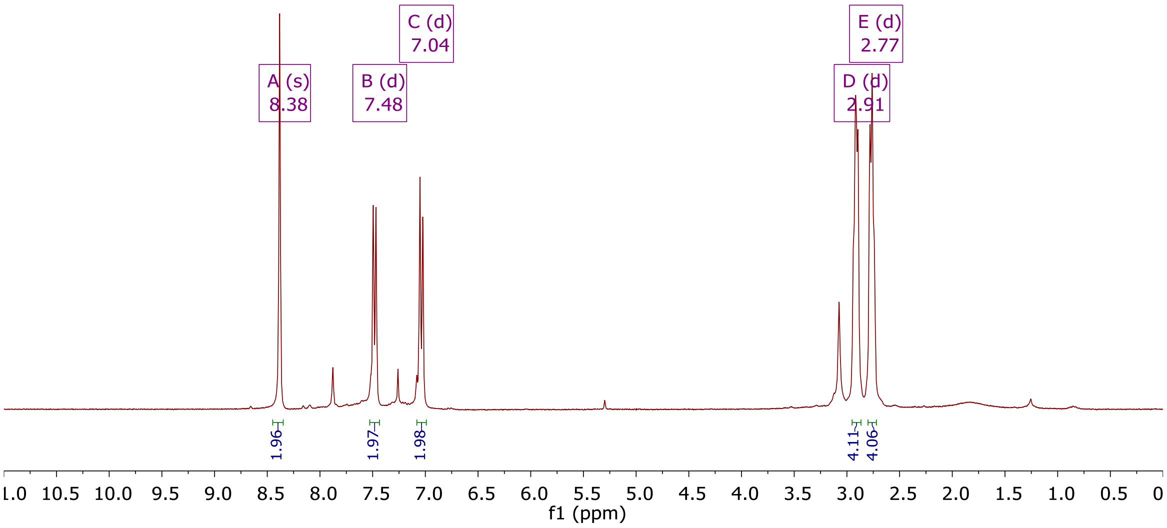

^{1}H NMR of *Z*-1 in $CDCl_3$

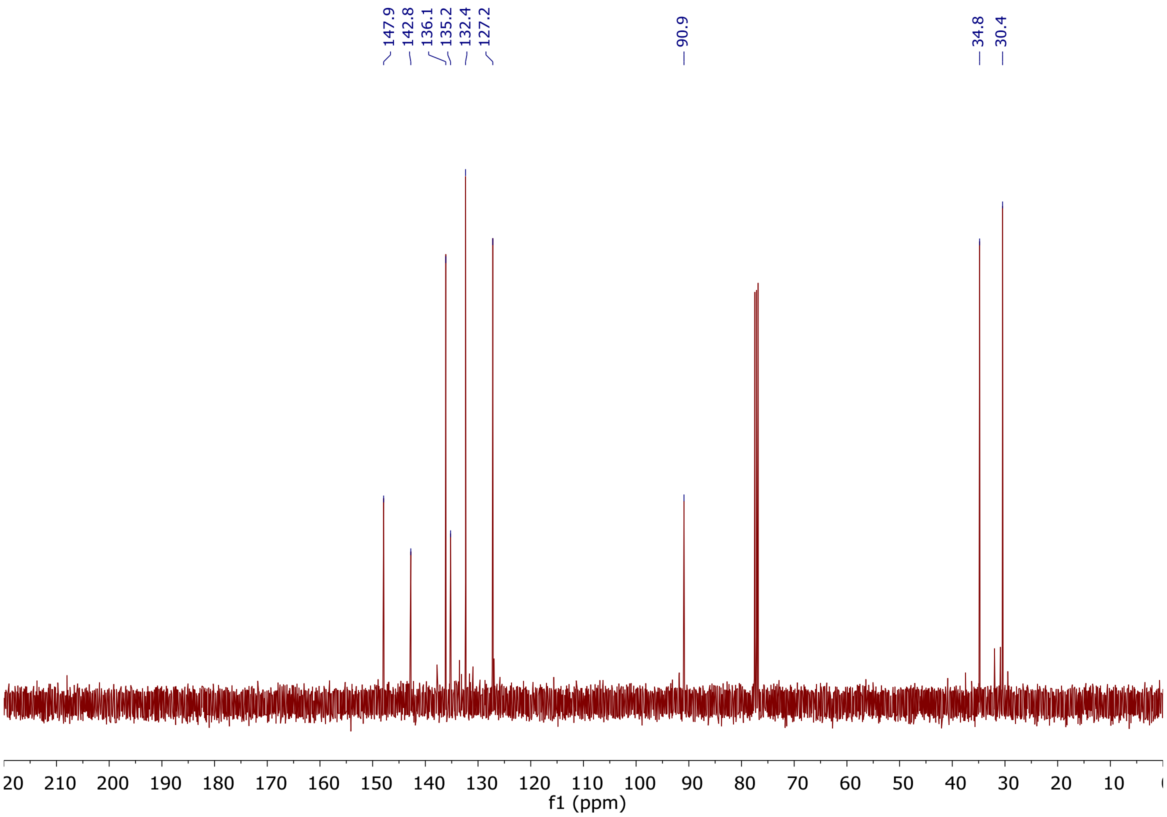

^{13}C NMR of *Z*-1 in $CDCl_3$

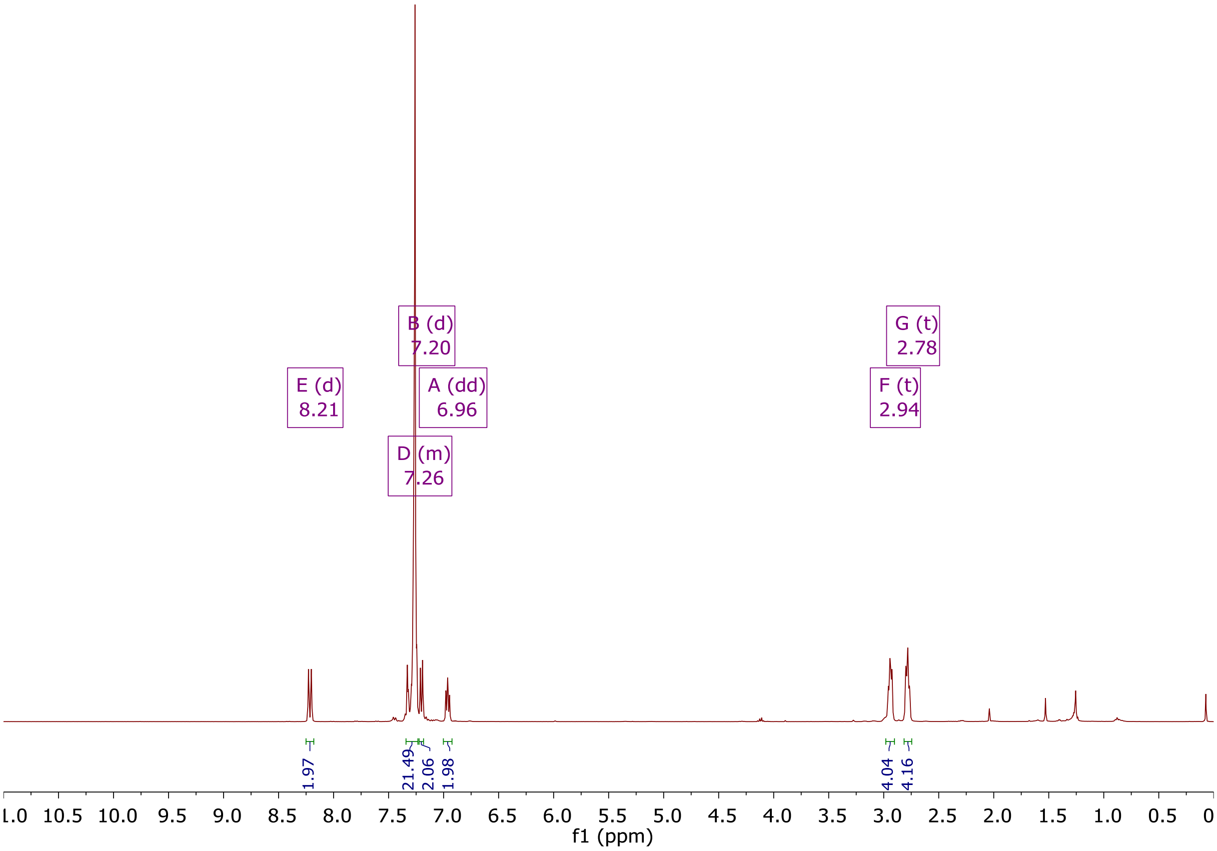

^{1}H NMR of *Z*-2 in $CDCl_3$

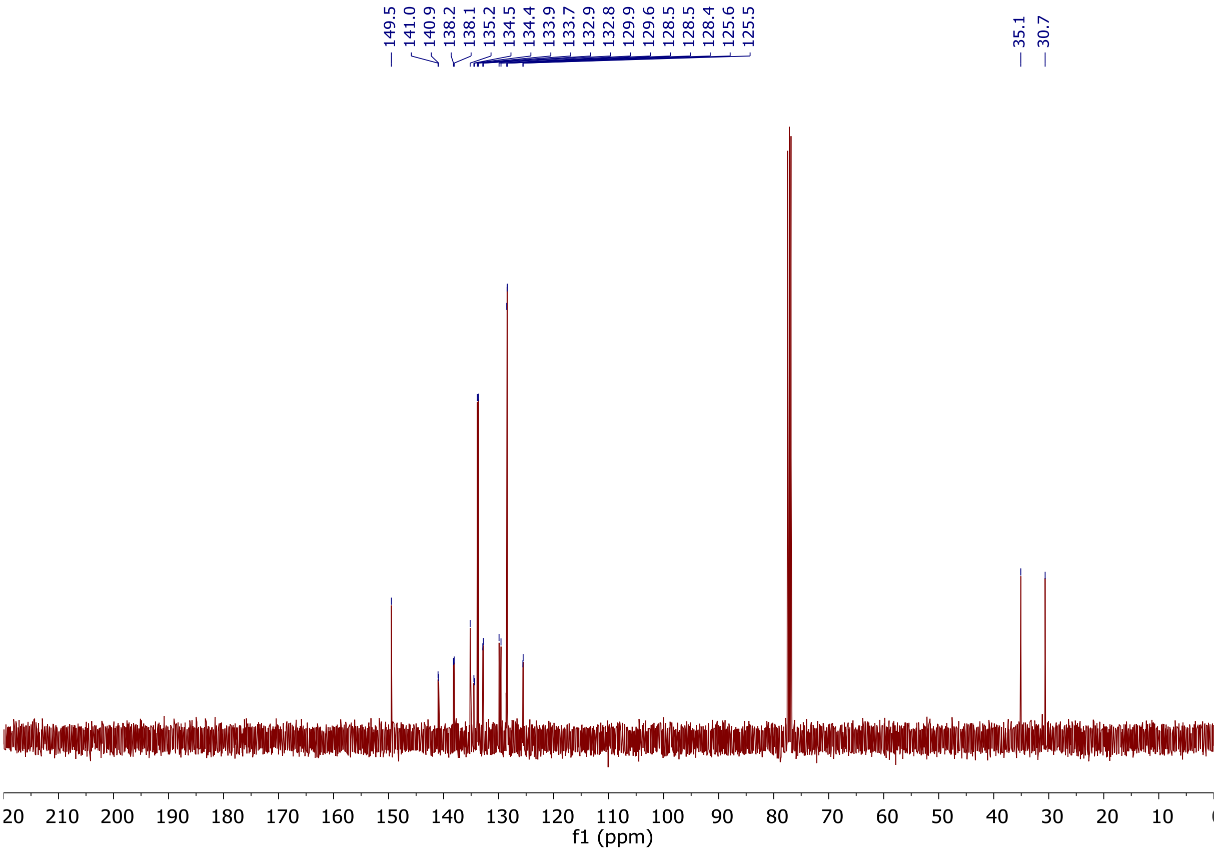

^{13}C NMR of *Z*-2 in $CDCl_3$

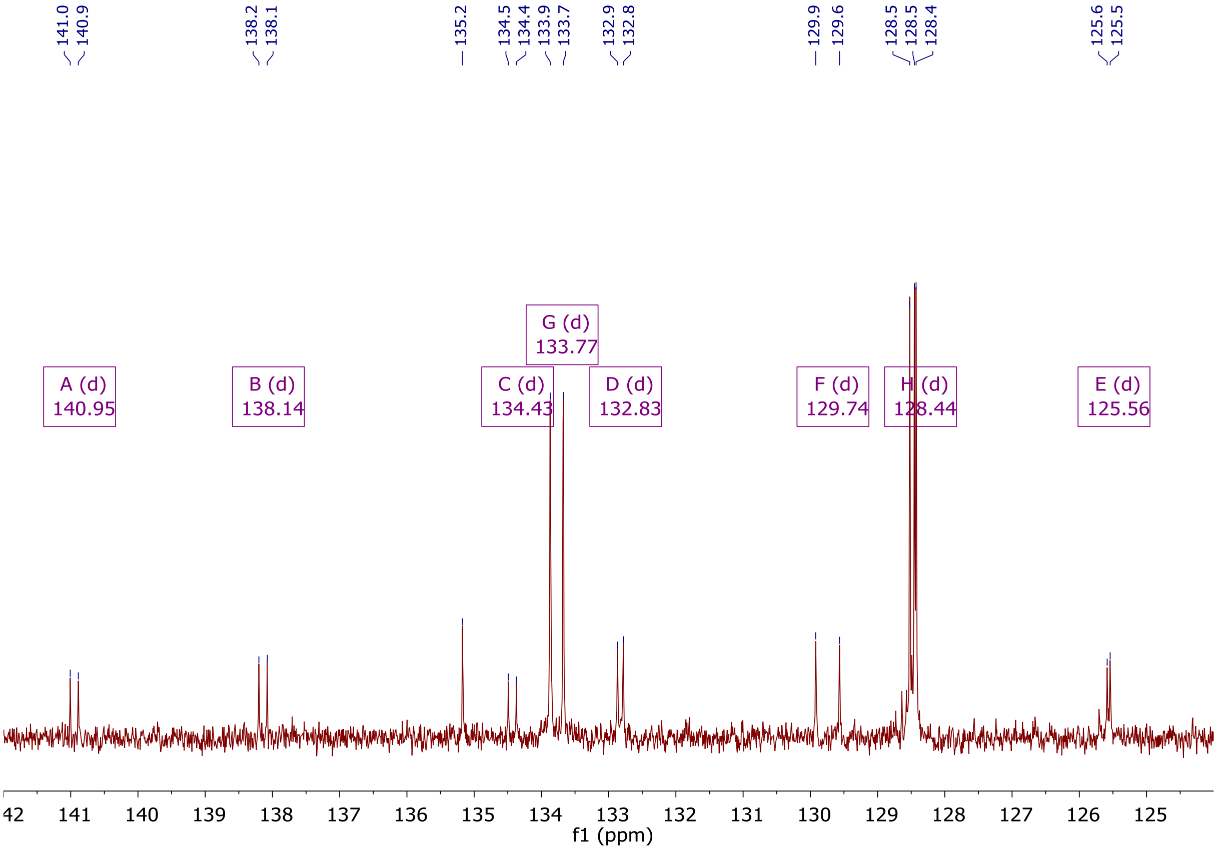

^{13}C NMR of *Z*-2 in $CDCl_3$; zoom on the $J_{P\text{-}C}$ area

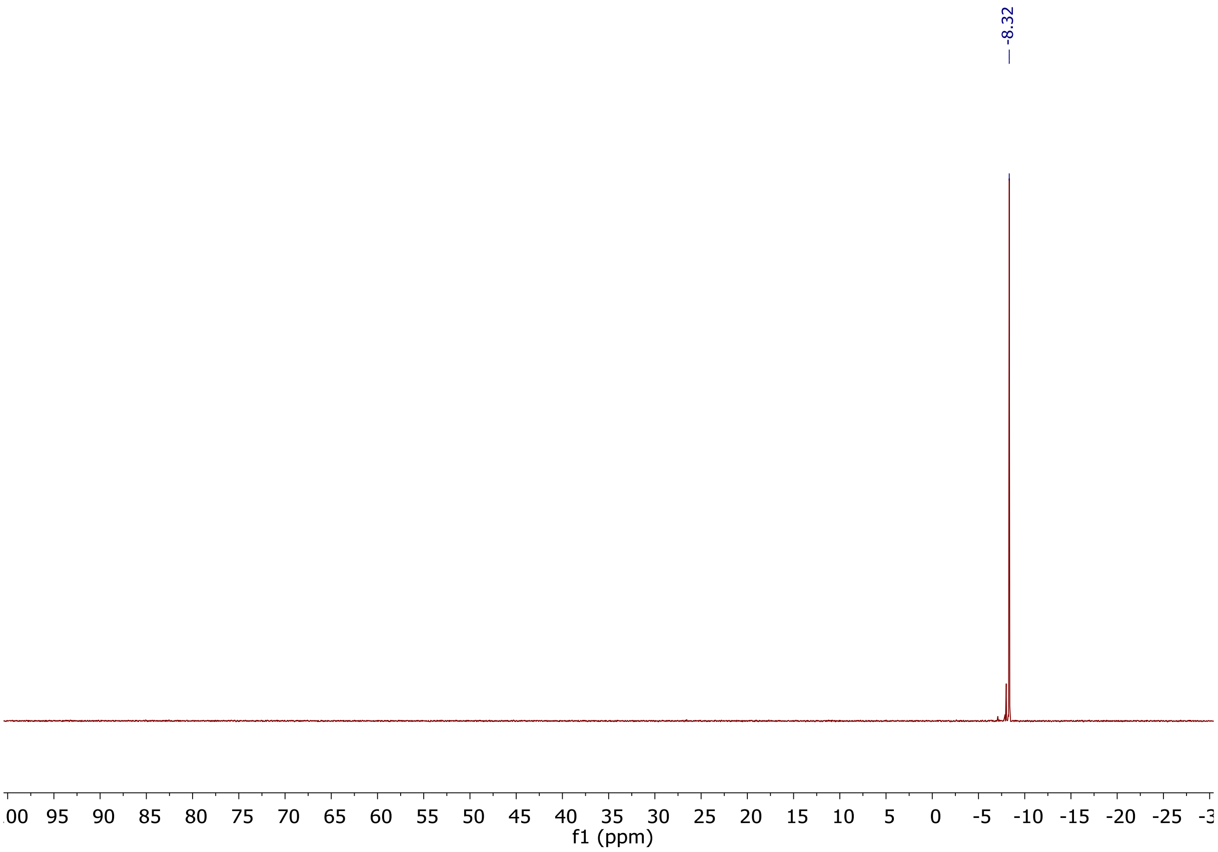

^{31}P NMR of *Z*-2 in $CDCl_3$

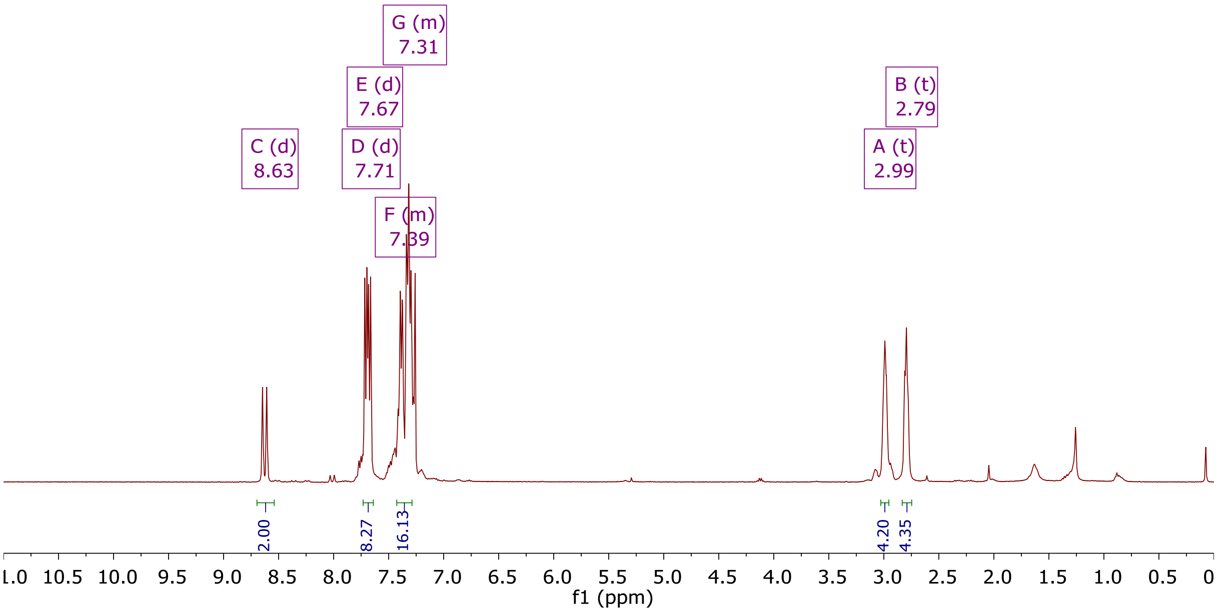

^{1}H NMR of *Z*-2Se in $CDCl_3$

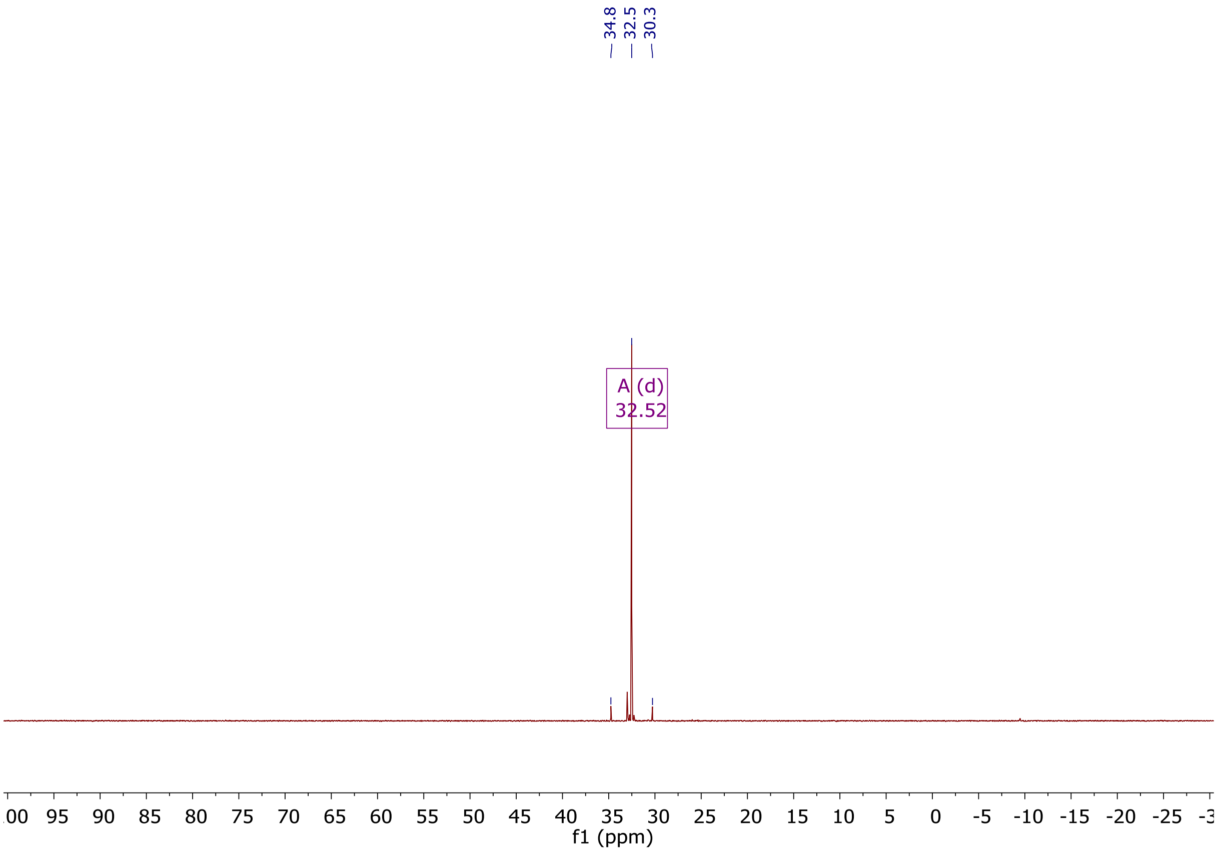

^{31}P NMR of *Z*-2Se in $CDCl_3$

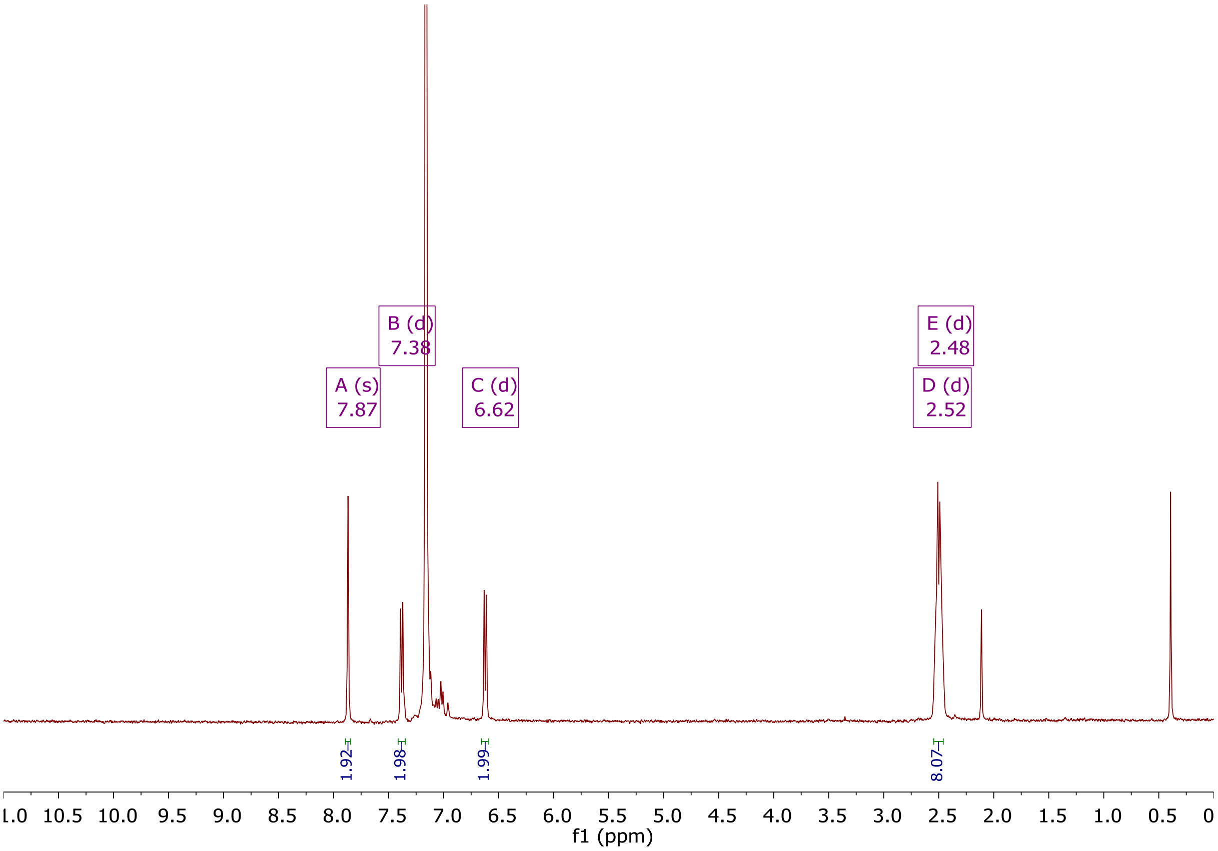

^{1}H NMR of *E*-1 in C_6D_6

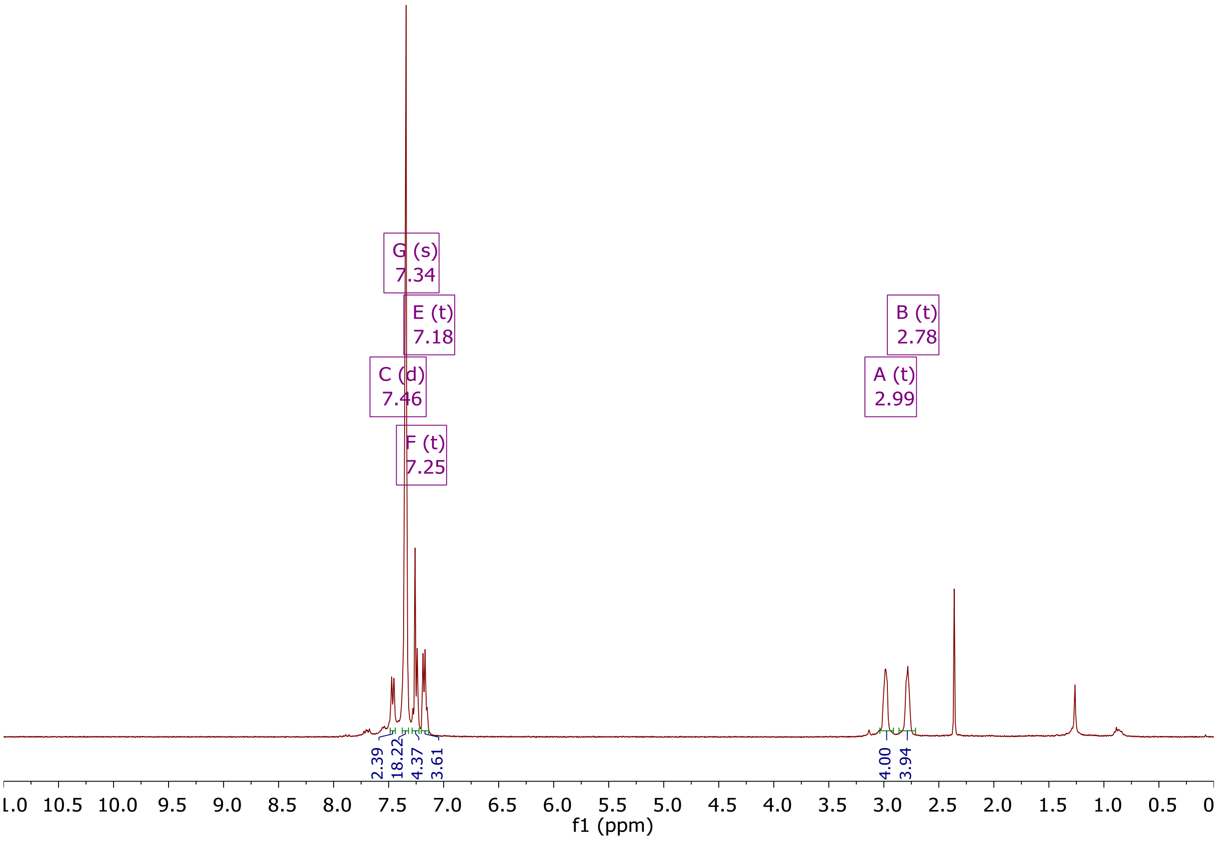

^{1}H NMR of *E*-2 in $CDCl_3$

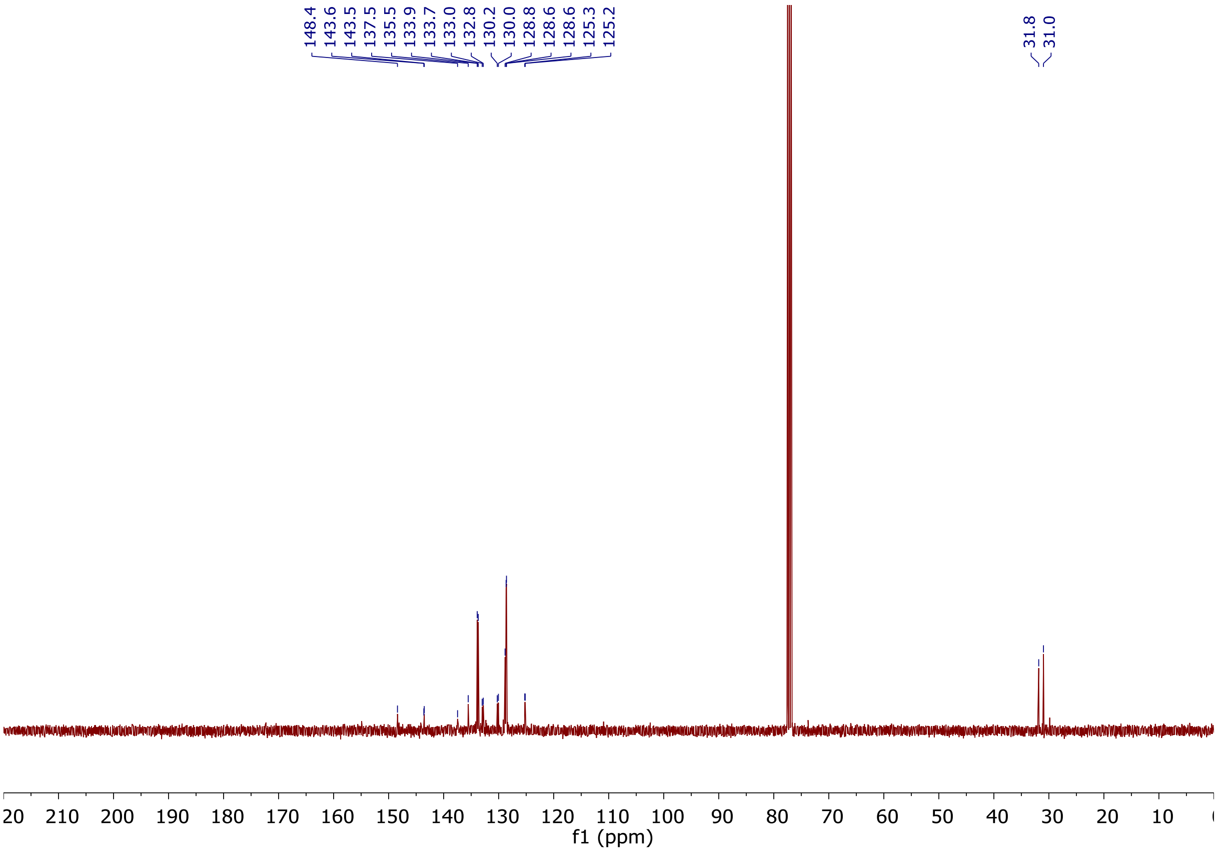

^{13}C NMR of *E*-2 in $CDCl_3$

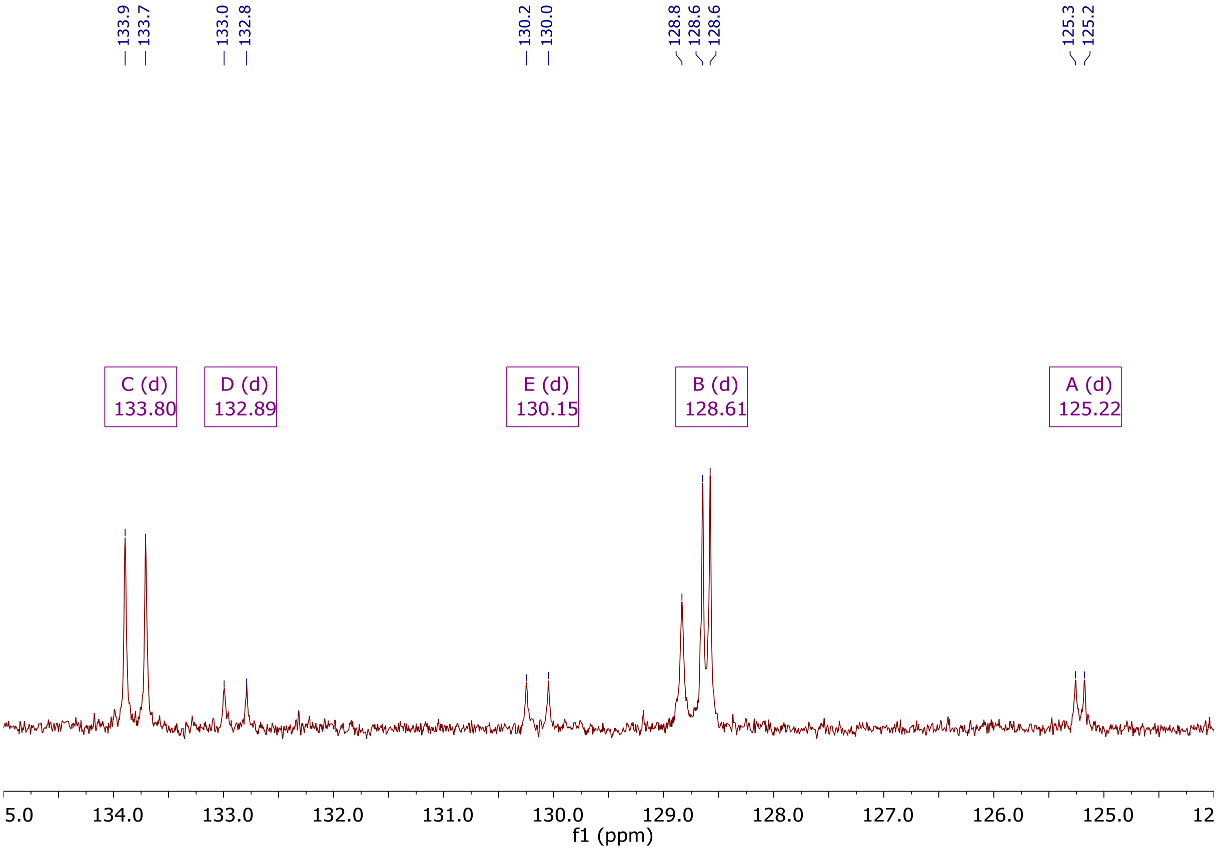

^{13}C NMR of *E*-2 in $CDCl_3$; zoom on the $J_{P\text{-}C}$ area

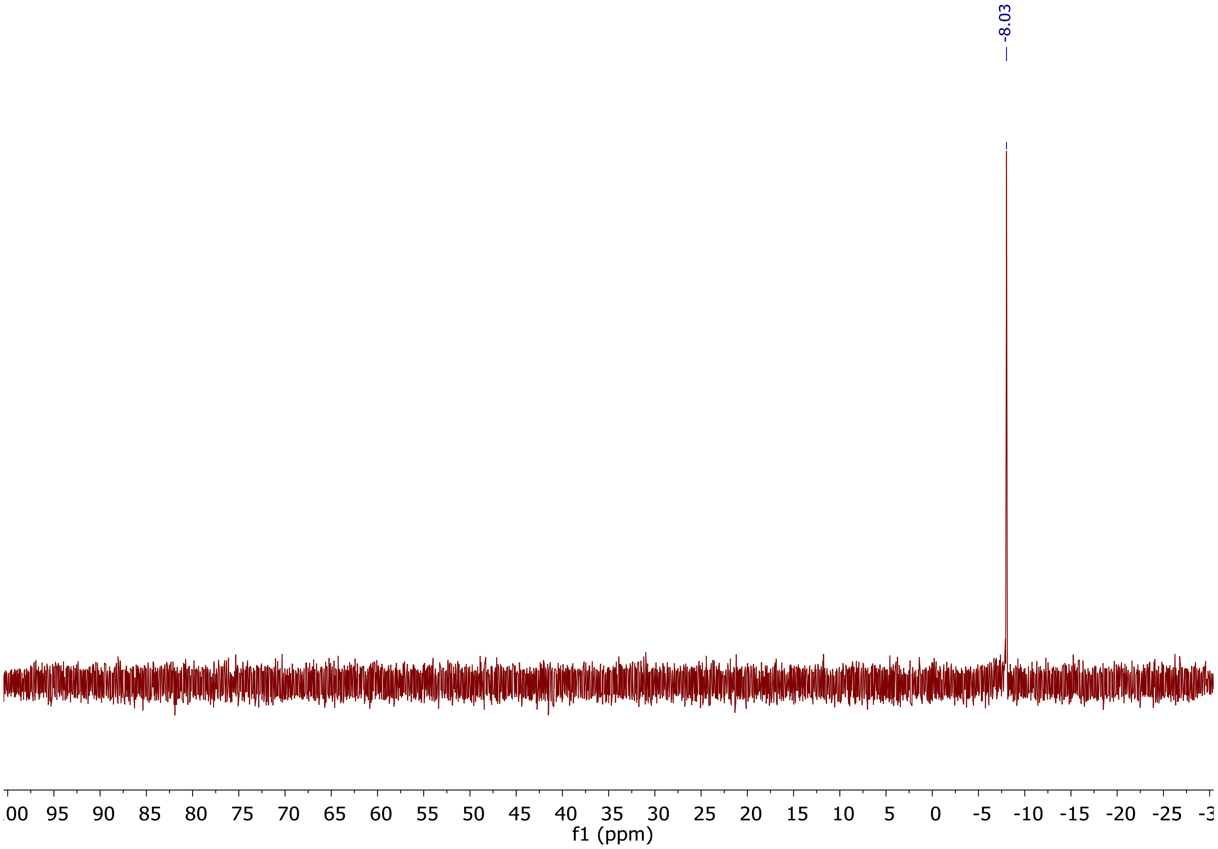

^{31}P NMR of *E*-2 in $CDCl_3$

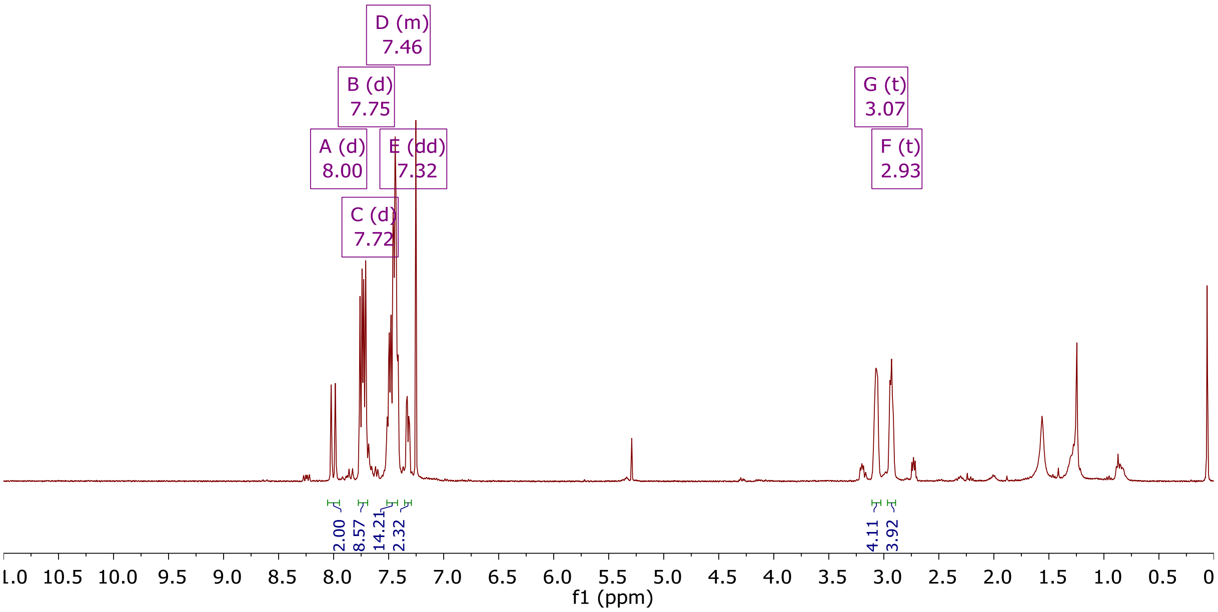

^{1}H NMR of *E*-2Se in CDCl$_3$

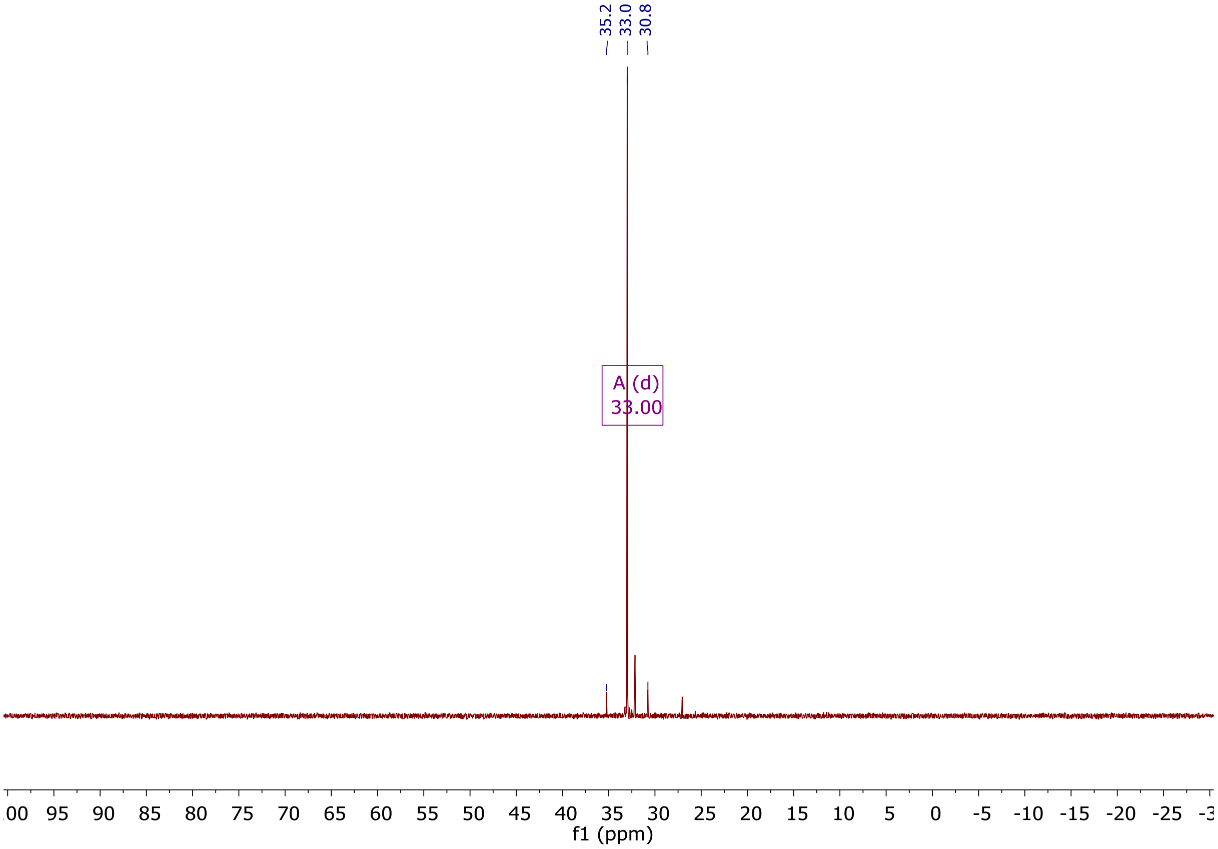

^{31}P NMR of *E*-2Se in CDCl$_3$

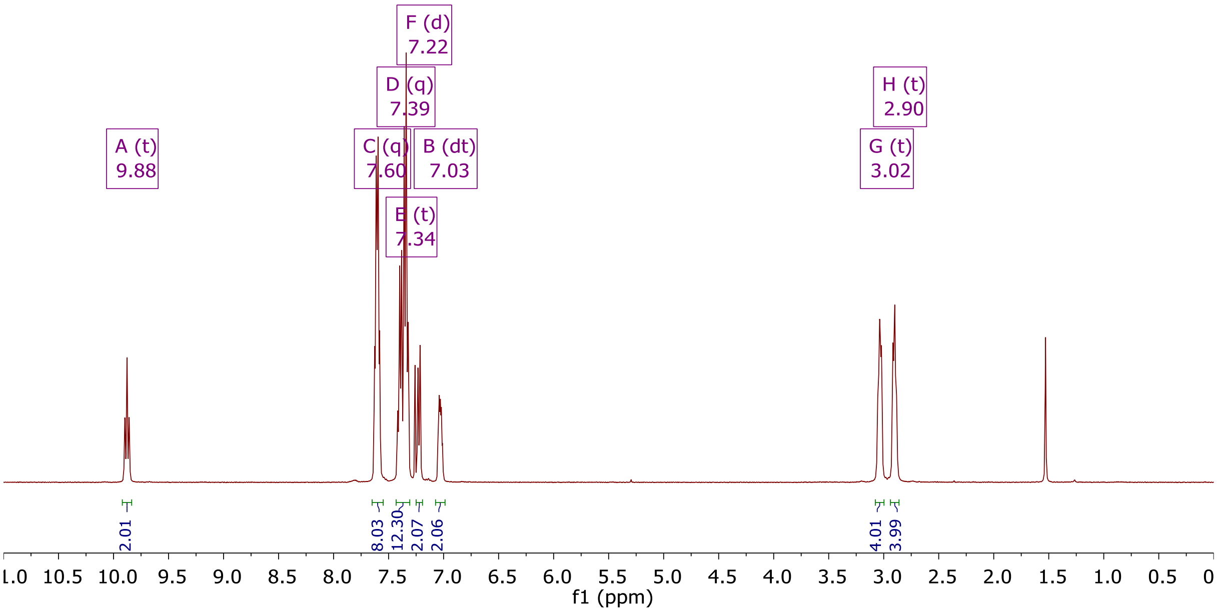

^{1}H NMR of *Z*-3 in $CDCl_3$

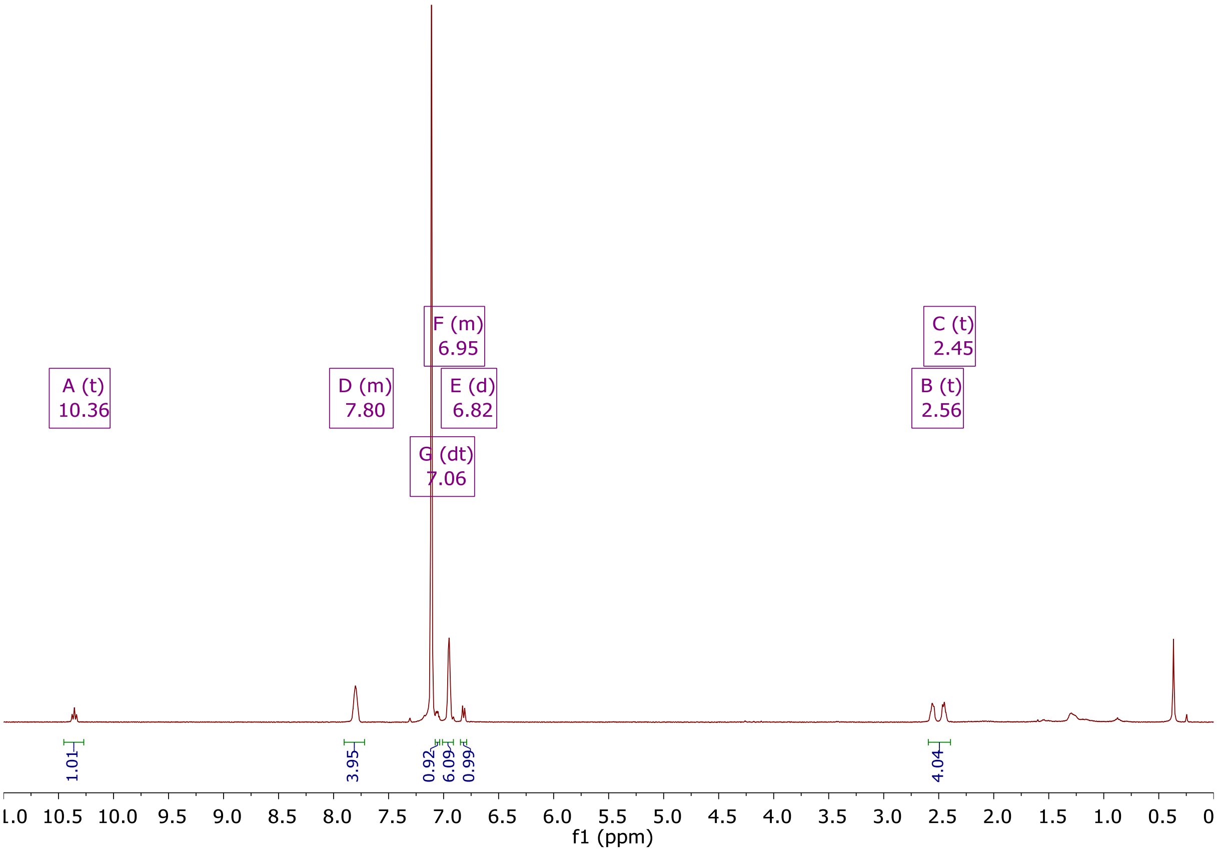

^{1}H NMR of *Z*-3 in C_6D_6

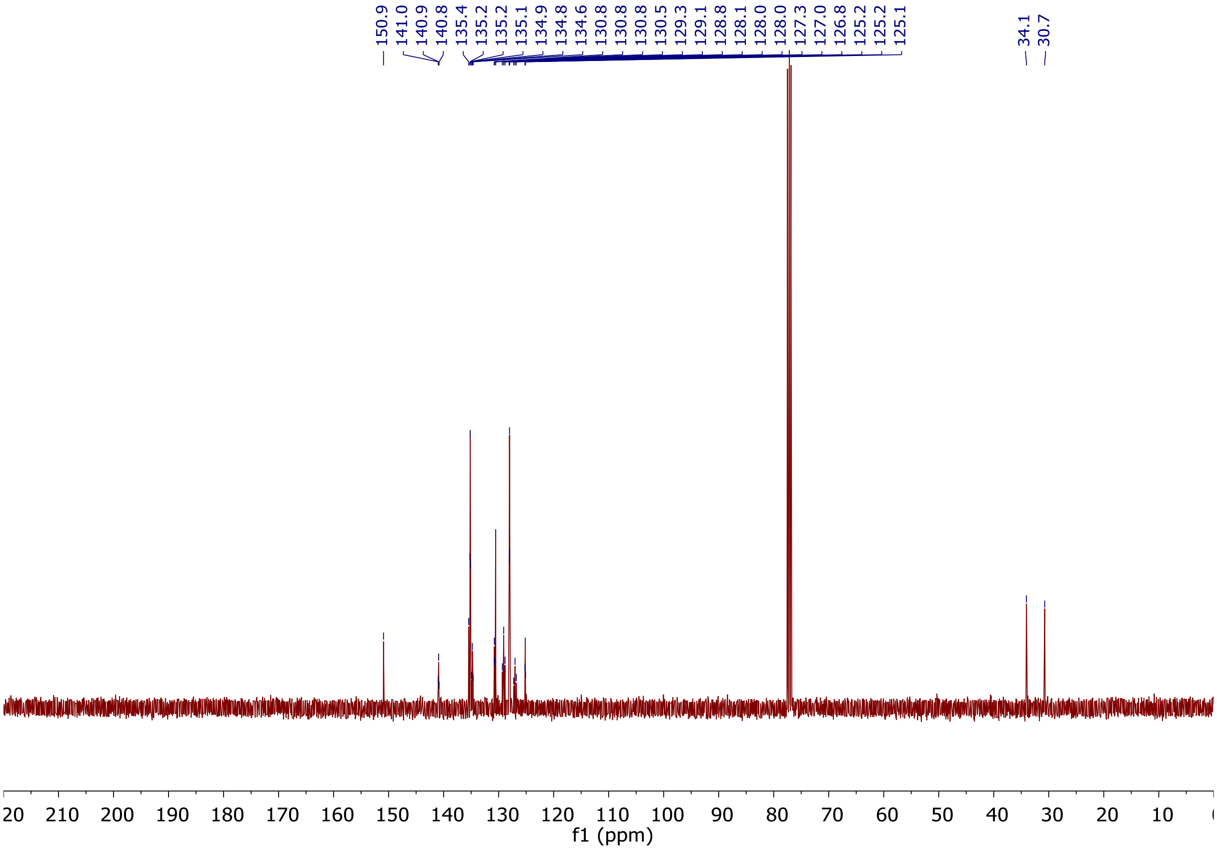

^{13}C NMR of *Z*-3 in $CDCl_3$

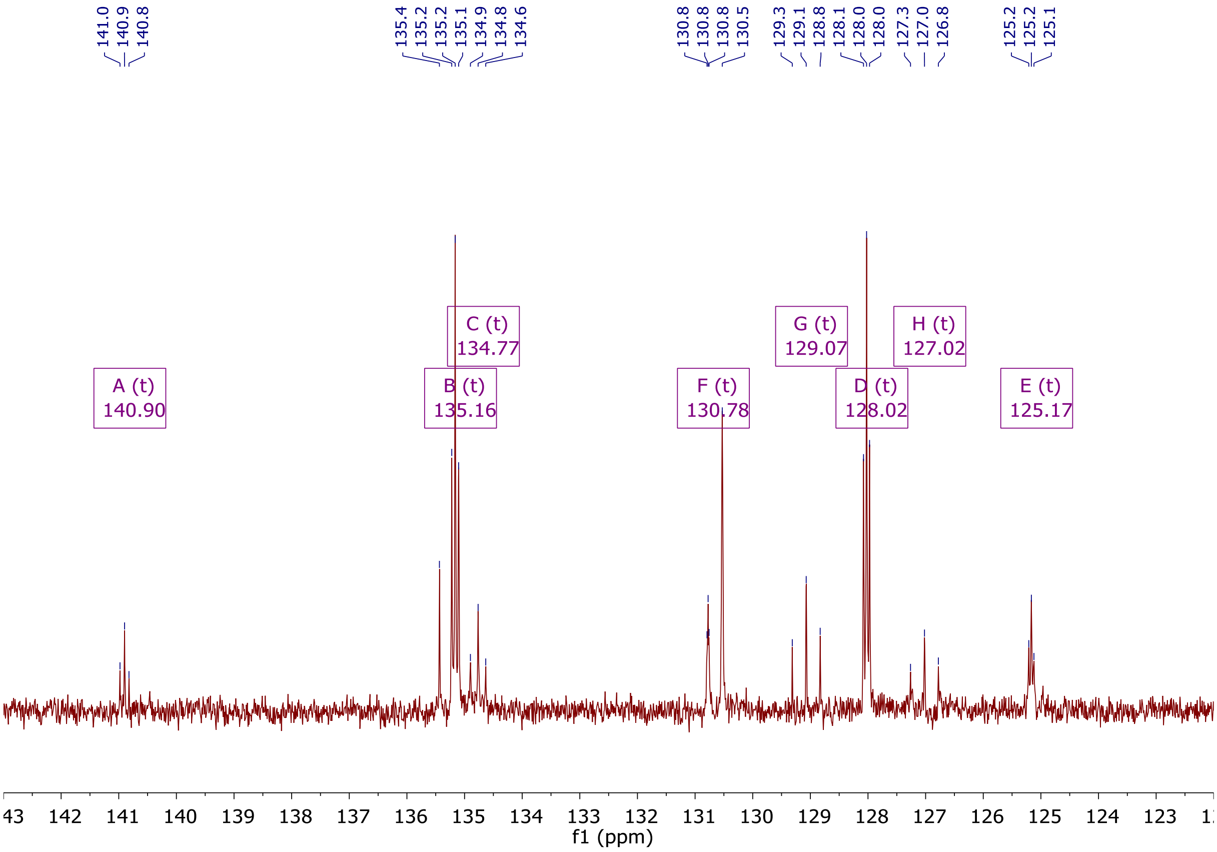

^{13}C NMR of *Z*-3 in $CDCl_3$; zoom on the $J_{P\text{-}C}$ area

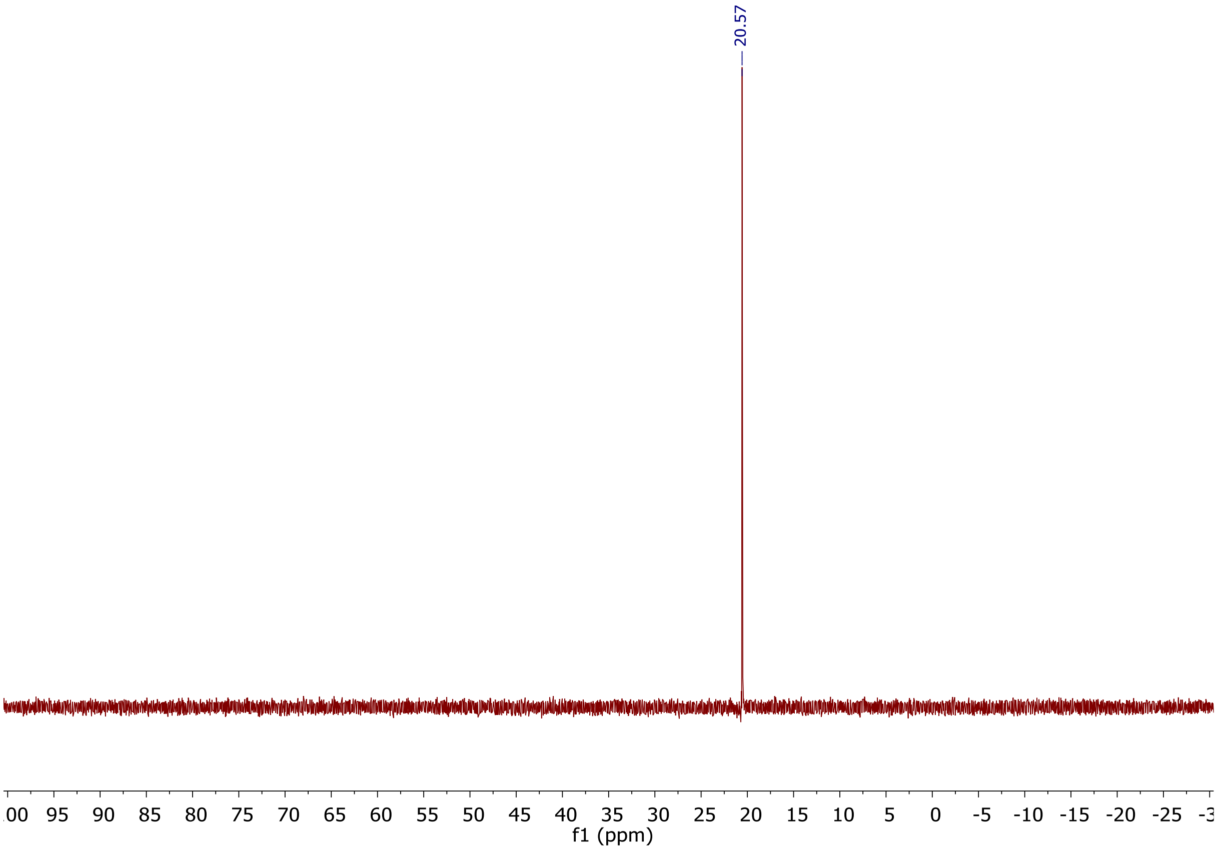

^{31}P NMR of *Z*-3 in $CDCl_3$

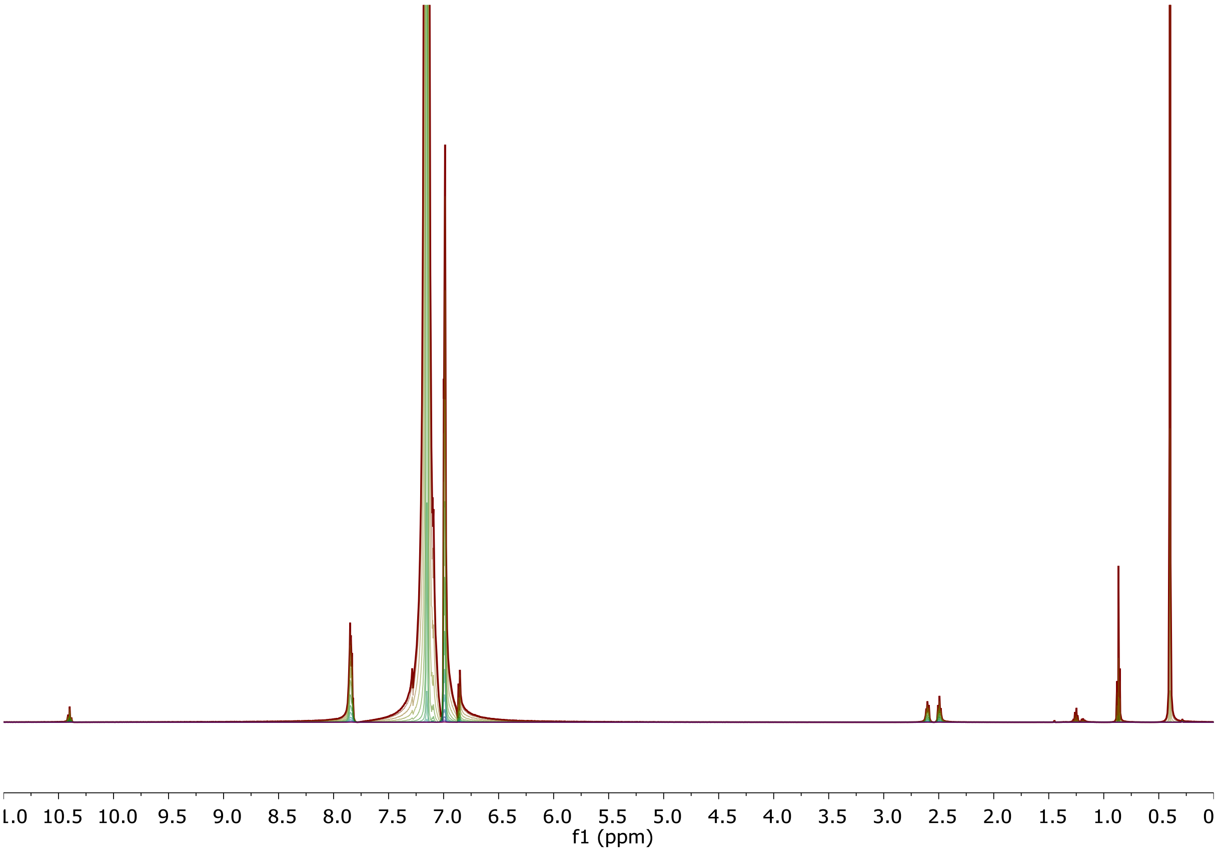

DOSY NMR of *Z*-3 in C_6D_6

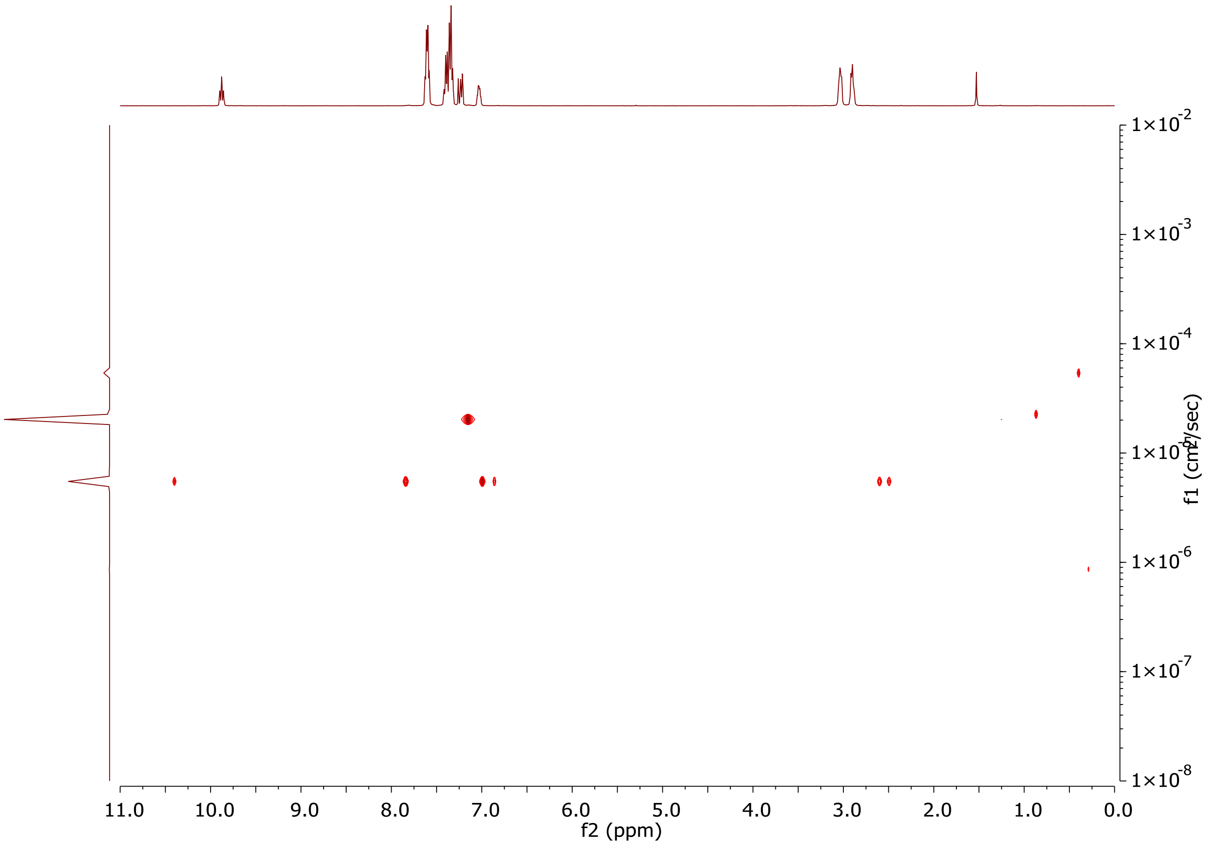

DOSY NMR of *Z*-3 in C_6D_6

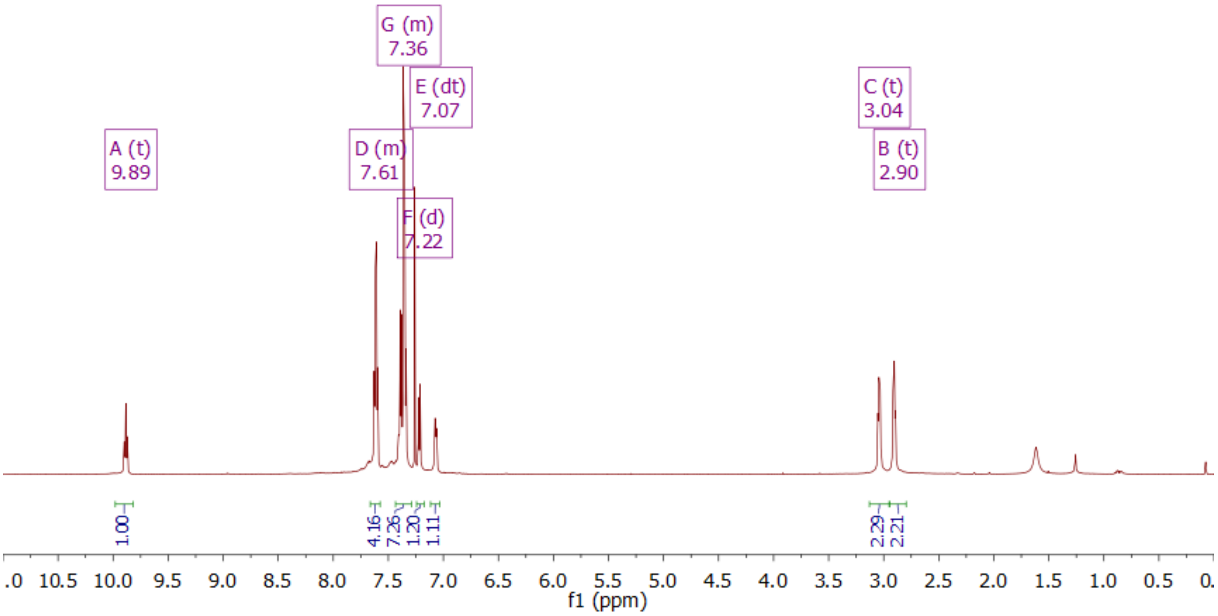

^{1}H NMR of *Z*-3Pt in $CDCl_3$

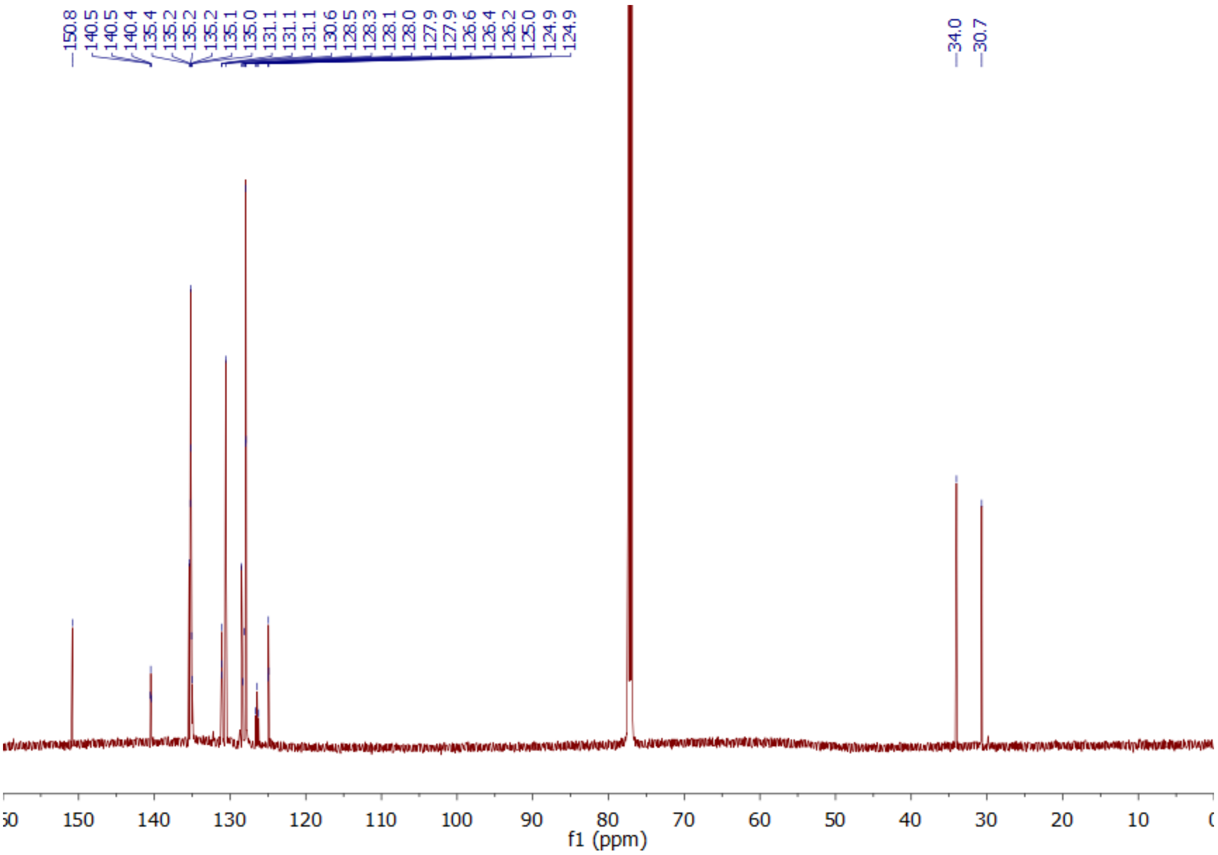

^{13}C NMR of *Z*-3Pt in $CDCl_3$

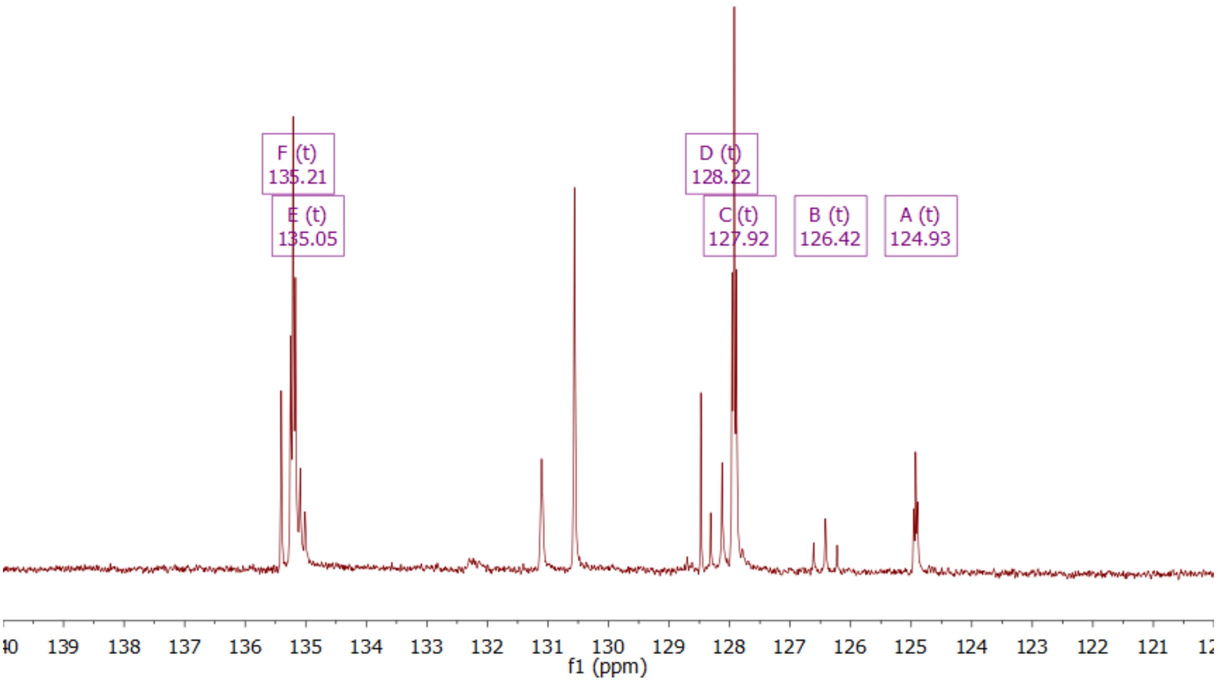

^{13}C NMR of *Z*-3Pt in $CDCl_3$; zoom on the $J_{P\text{-}C}$ area

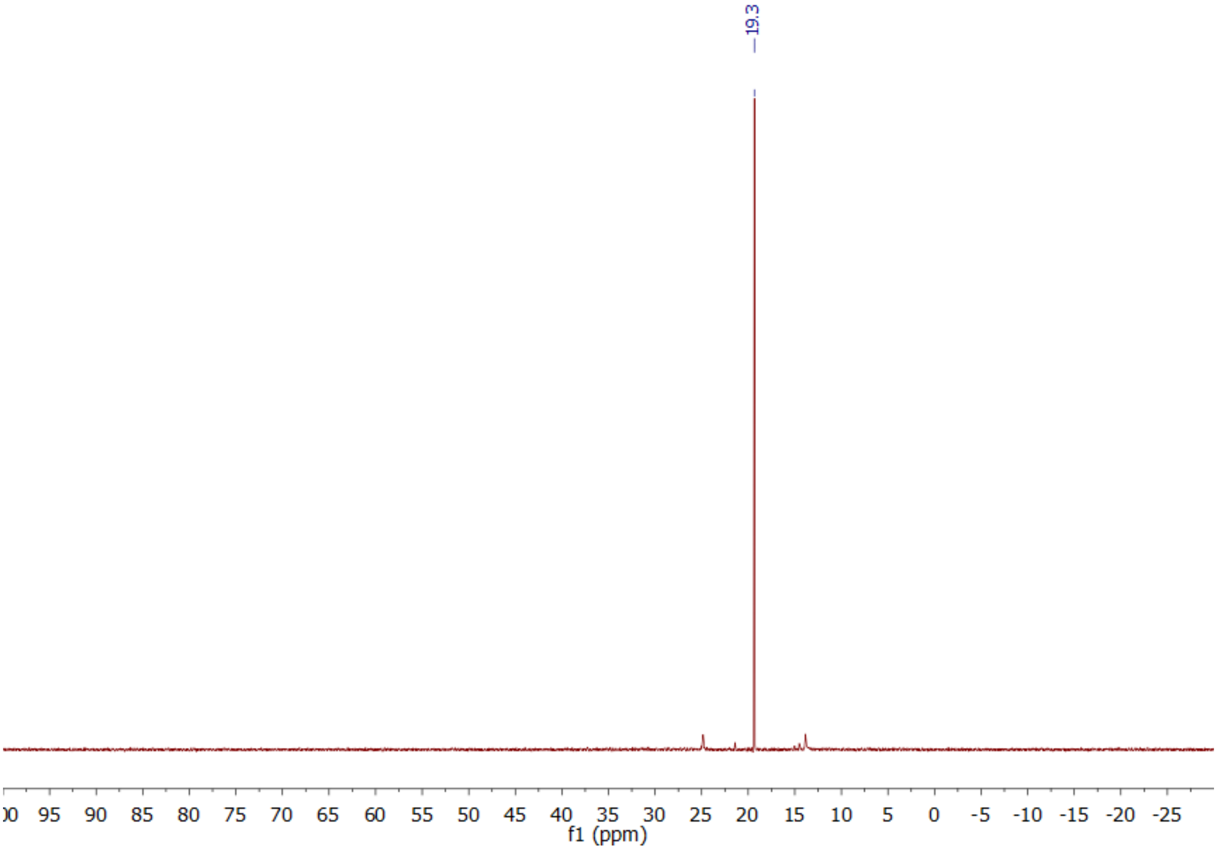

^{31}P NMR of *Z*-3Pt in $CDCl_3$

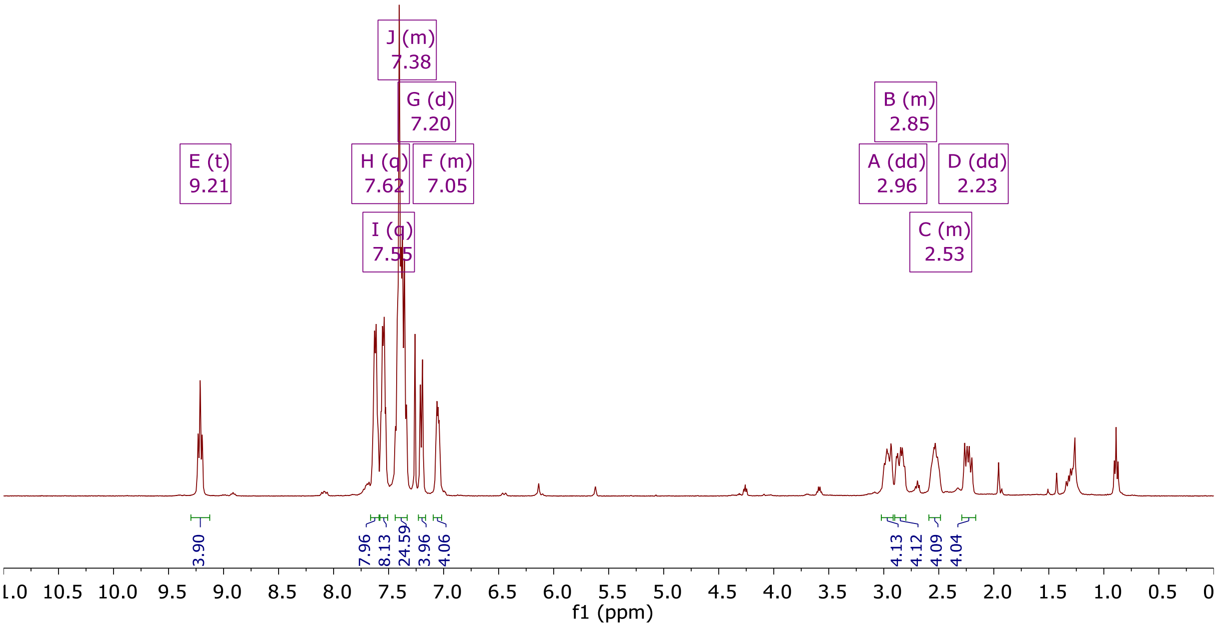

^{1}H NMR of *E*-3 in $CDCl_3$

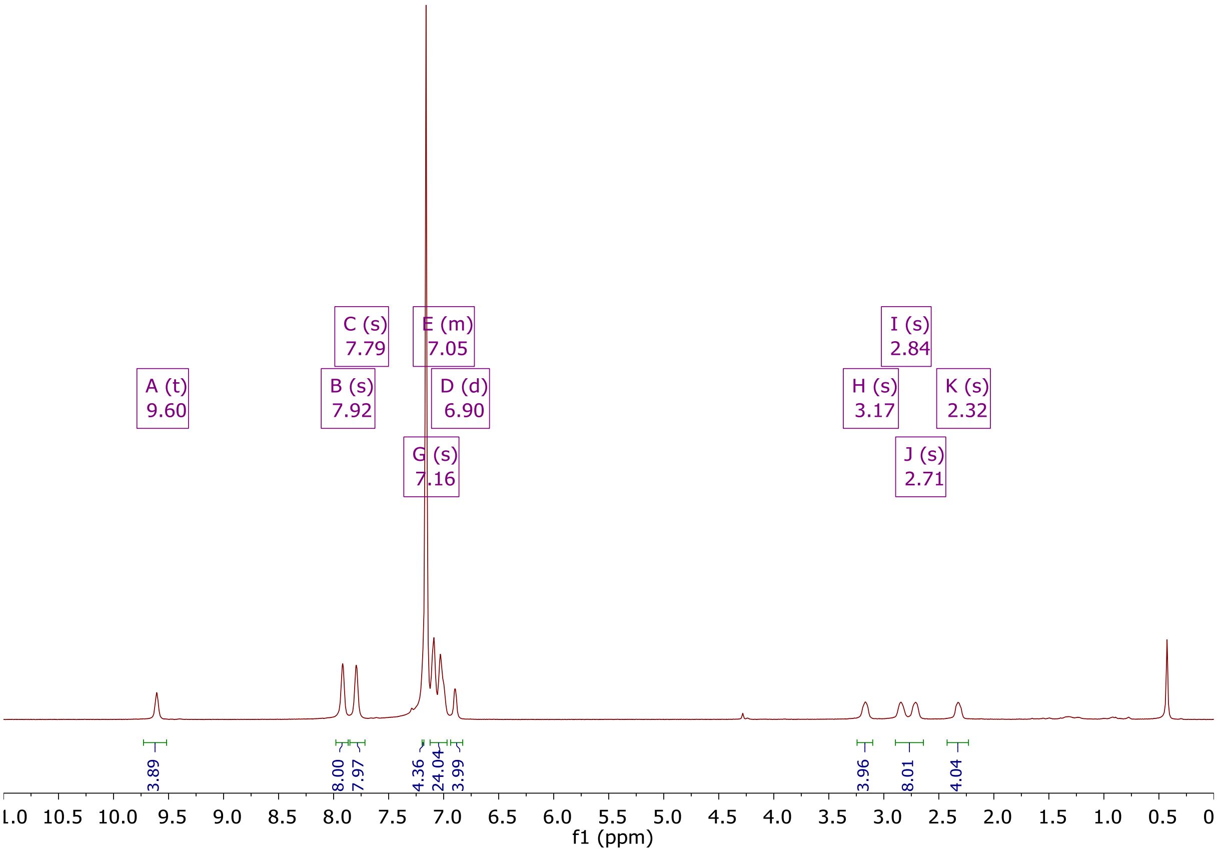

^{1}H NMR of *E*-3 in C_6D_6

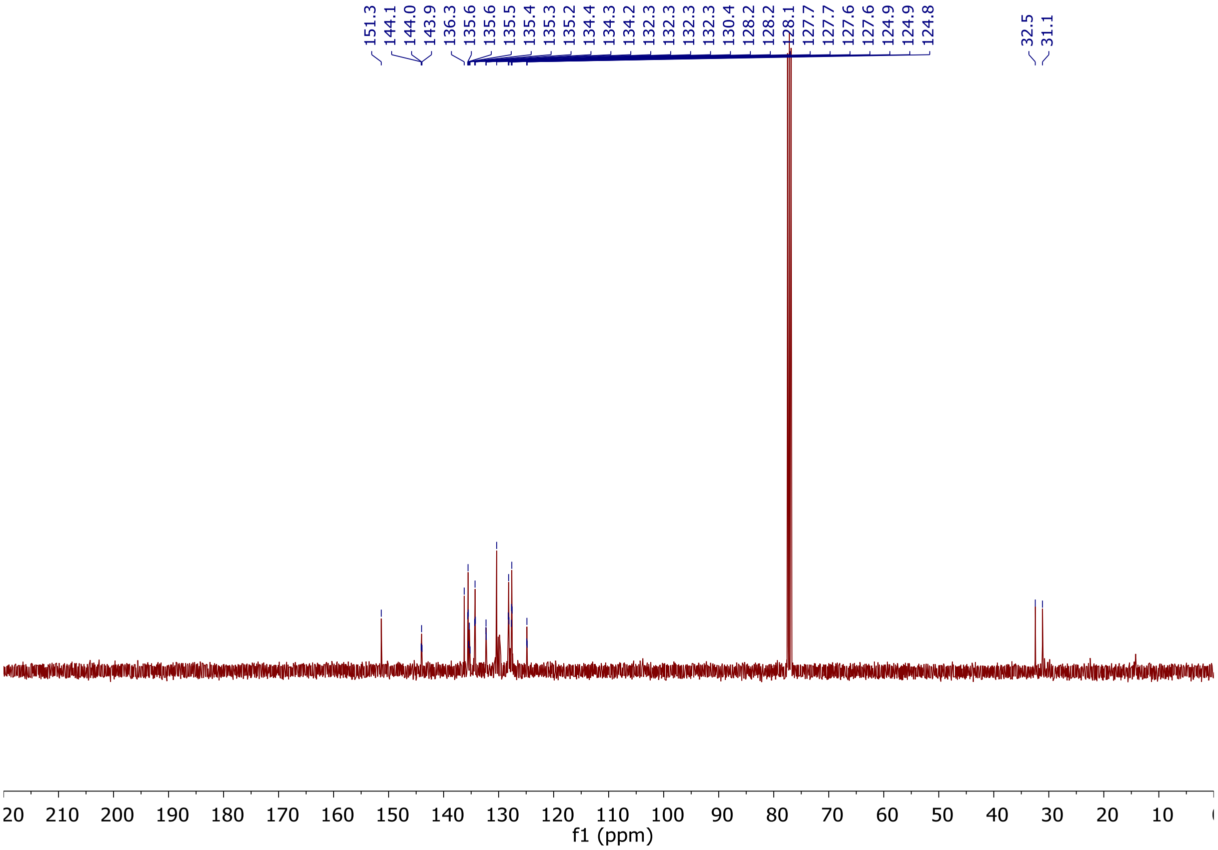

^{13}C NMR of *E*-3 in $CDCl_3$

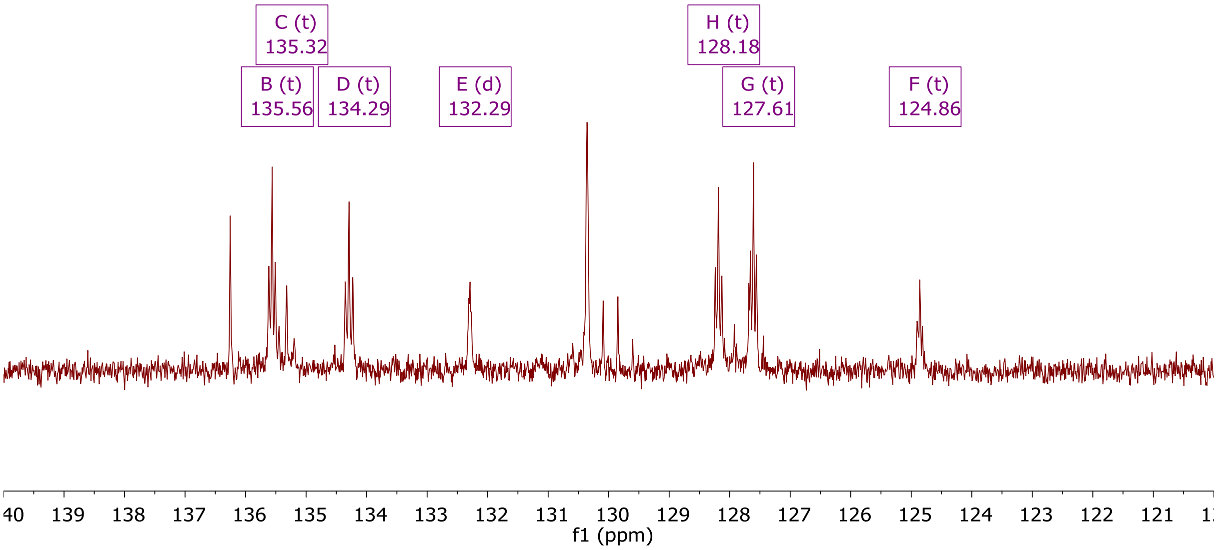

^{13}C NMR of *E*-3 in $CDCl_3$; zoom on the $J_{P\text{-}C}$ area

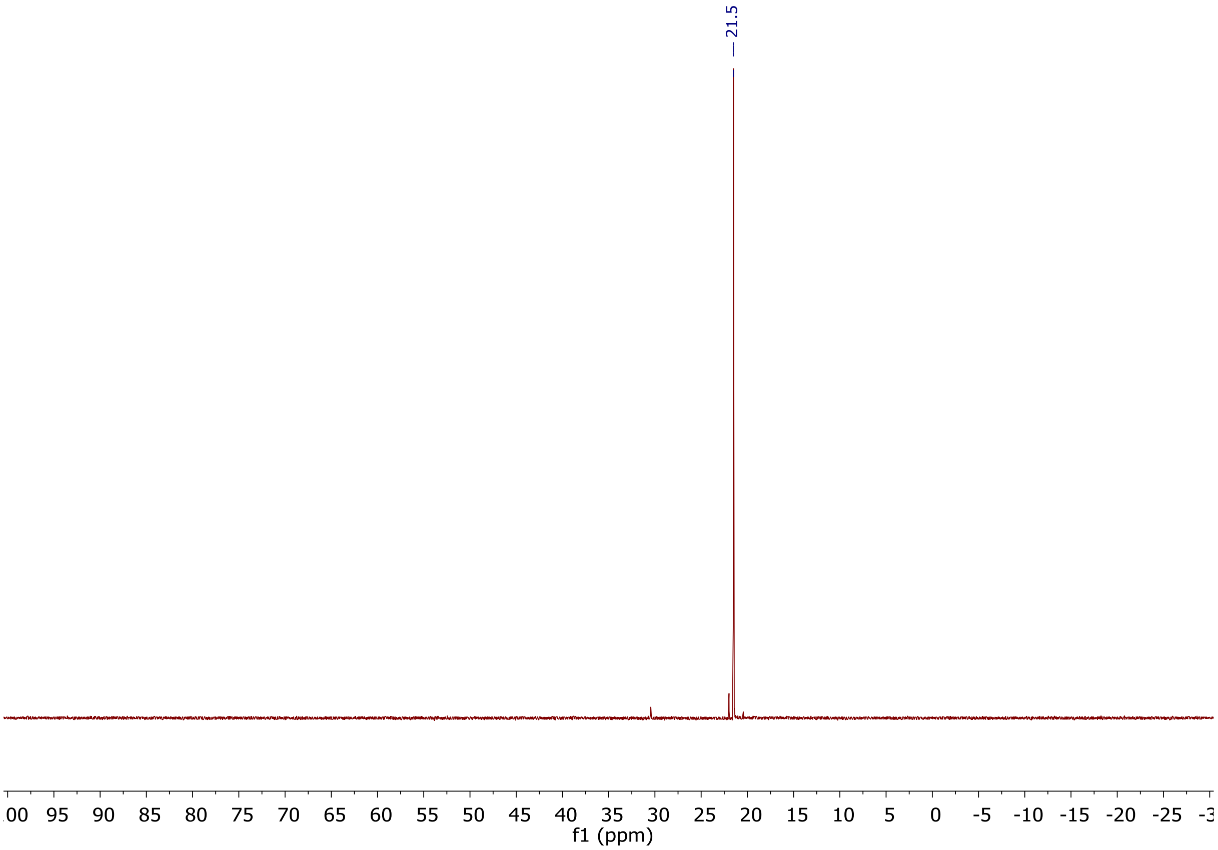

^{31}P NMR of *E*-3 in $CDCl_3$

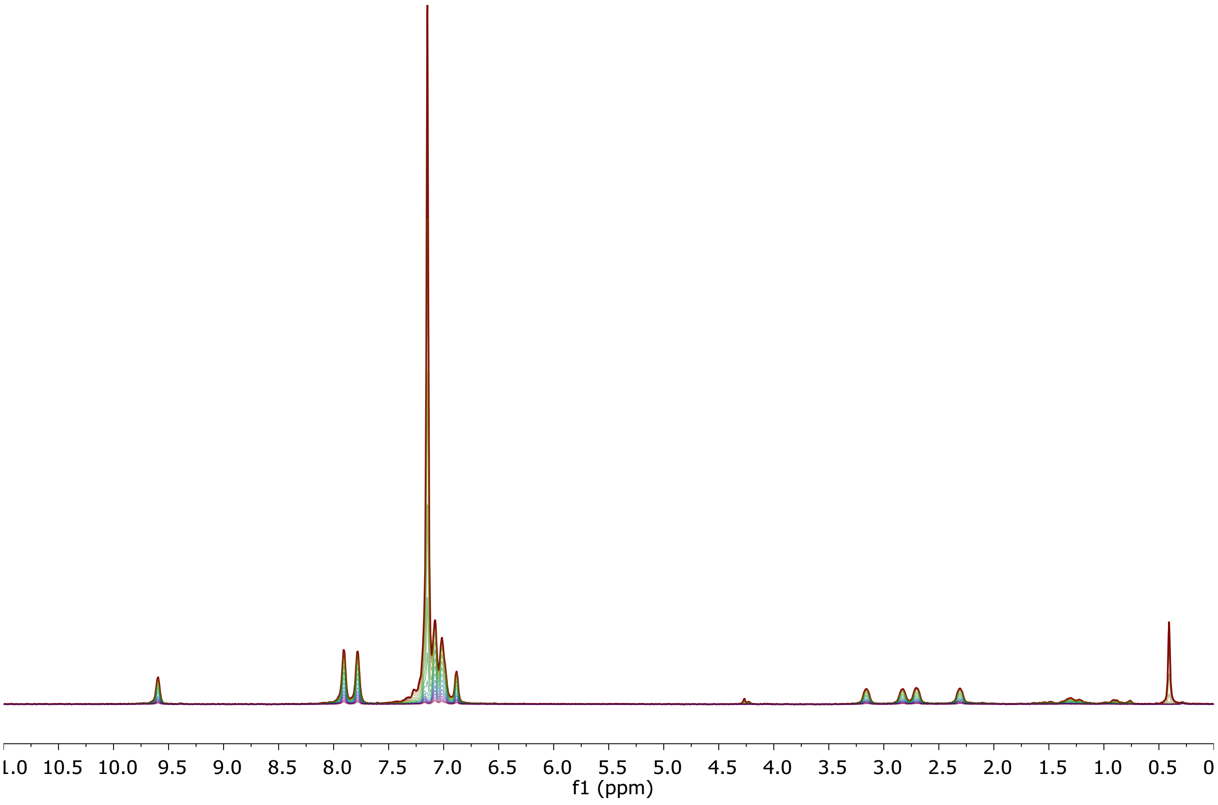

DOSY NMR of *E*-3 in C_6D_6

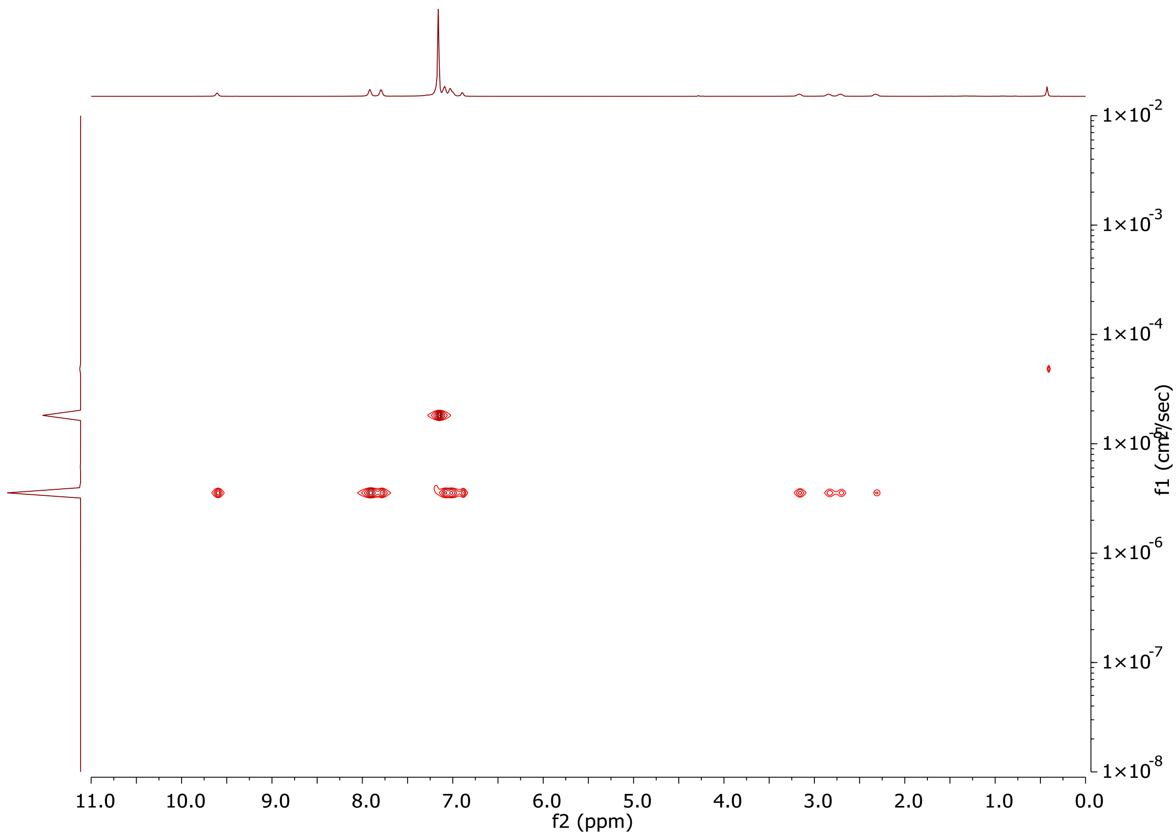

DOSY NMR of *E*-3 in C_6D_6

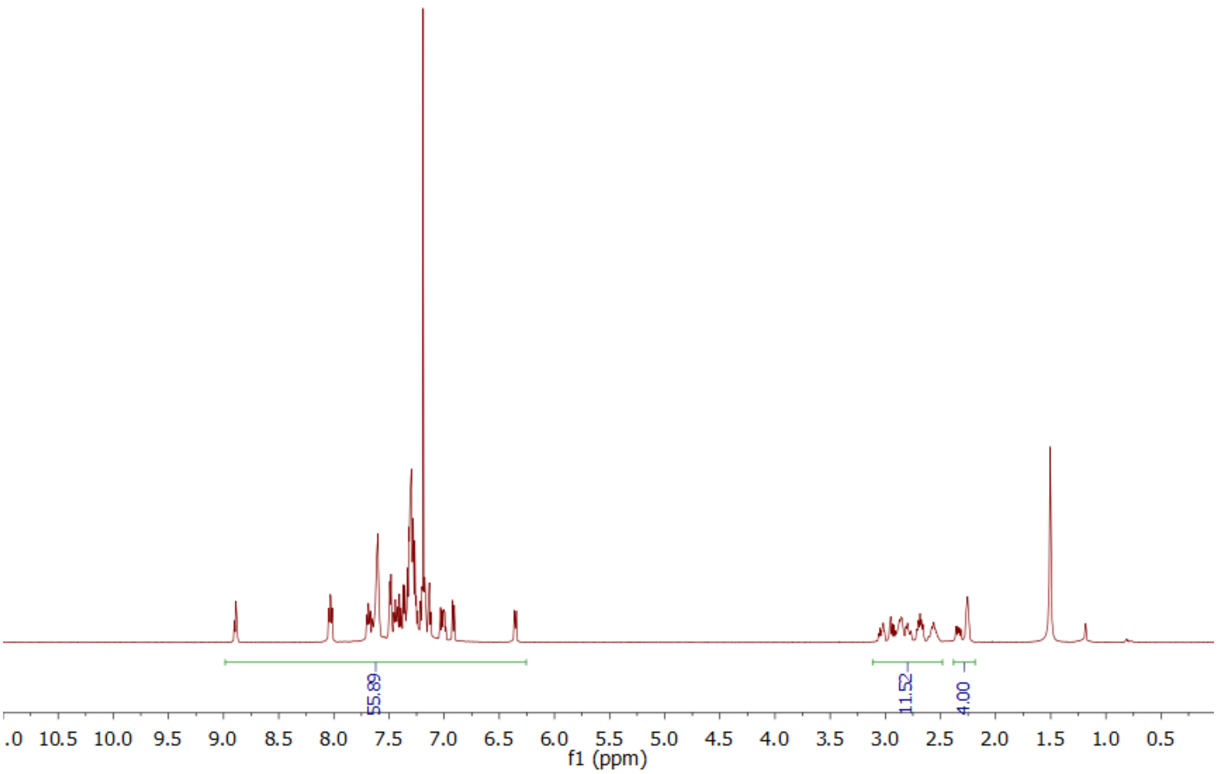

^{1}H NMR of *E*-3Pt in $CDCl_3$

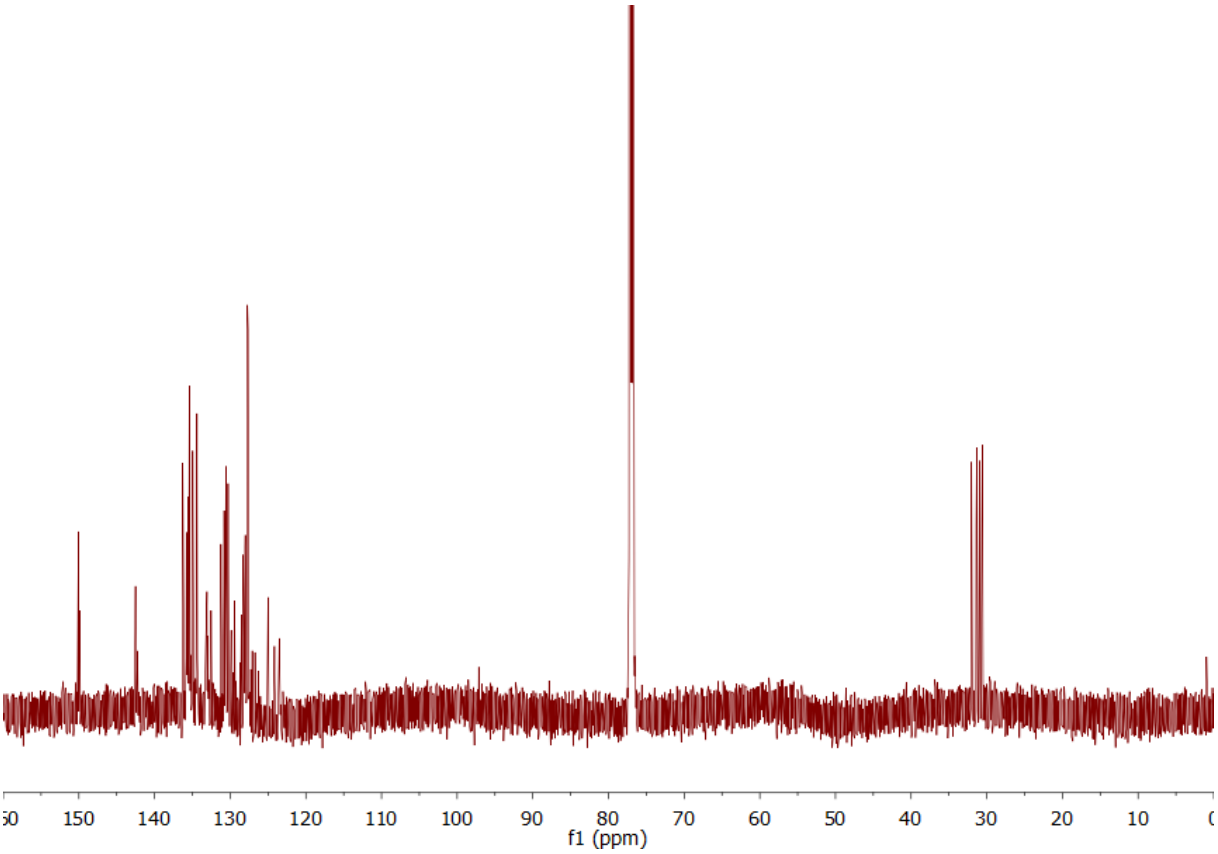

^{13}C NMR of *E*-3Pt in $CDCl_3$

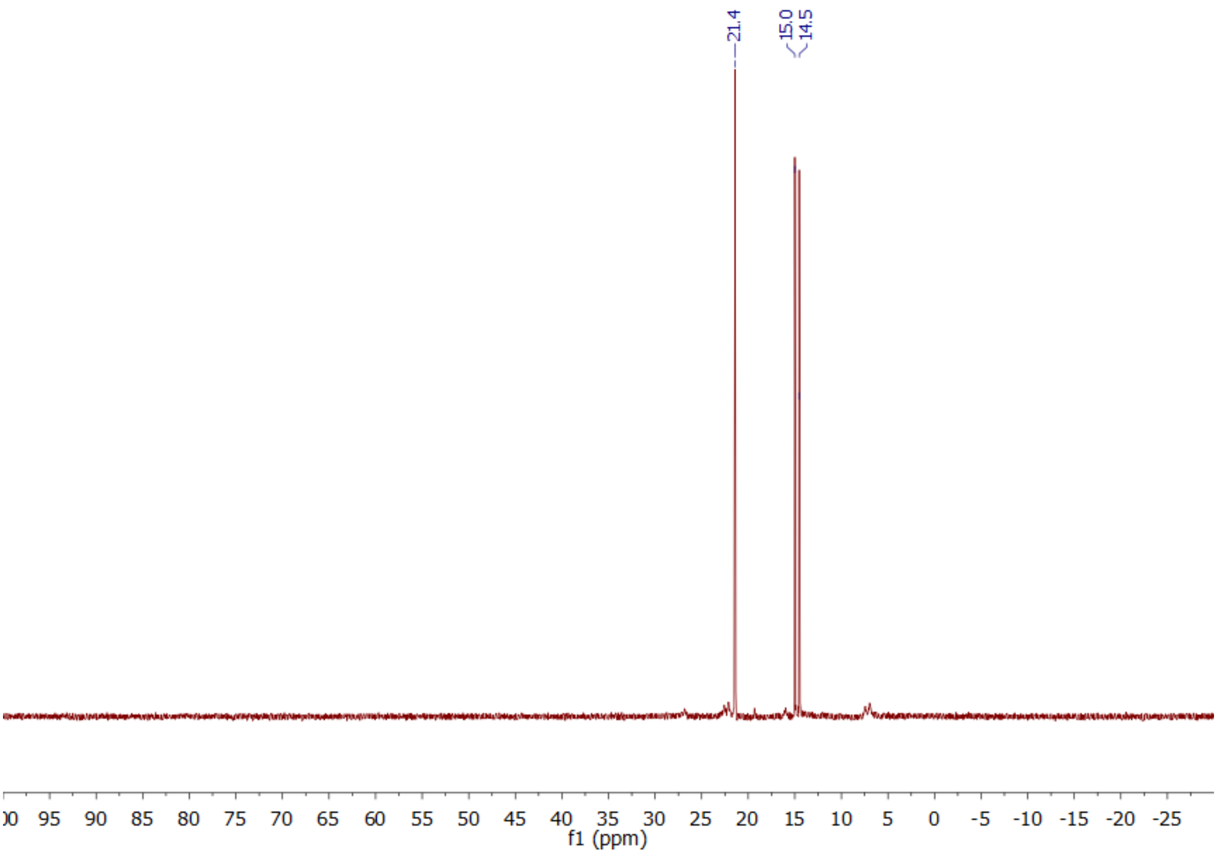

^{31}P NMR of *E*-3Pt in $CDCl_3$

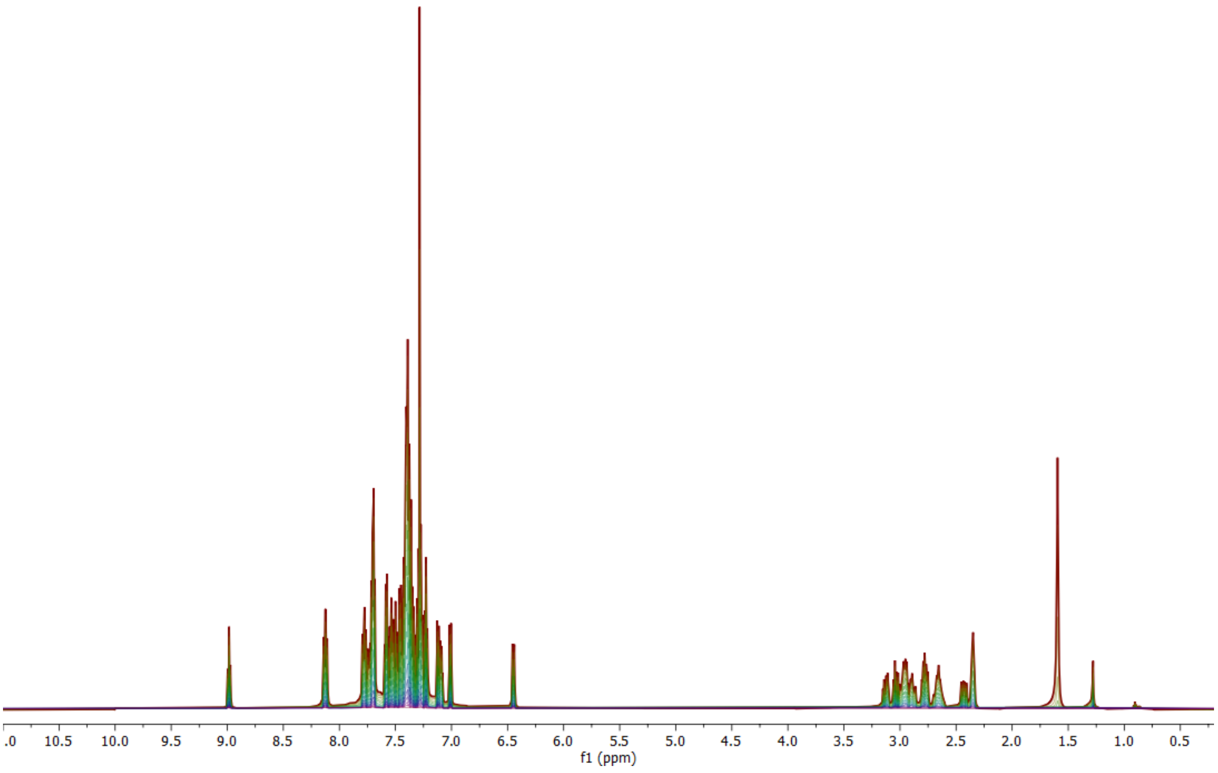

DOSY NMR of *E*-3Pt in $CDCl_3$

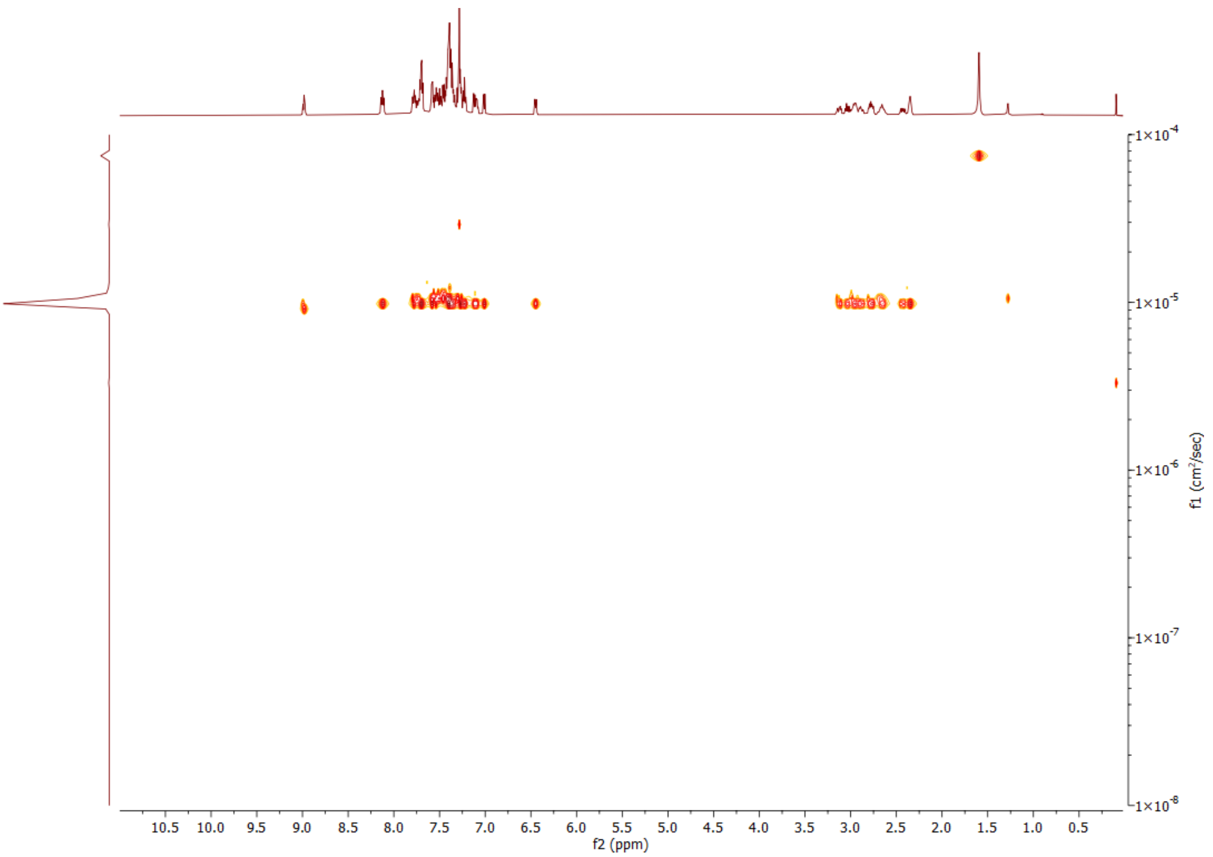

DOSY NMR of *E*-3Pt in $CDCl_3$

Variable-Temperature HPLC

Analytical chromatography was performed on a Shimadzu Prominence HPLC system. Enantiomers of ***E*-3** were separated on a Daicel CHIRALPAK IC-3 analytical column. Eluent: Heptane/CH_2Cl_2 70:30, 0.5 ml/min. The ΔG of isomerization was found to be 92.7 ± 0.6 kJ mol^{-1}. The 95% confidence interval of the measure is reported in the linearized Eyring plot graph.

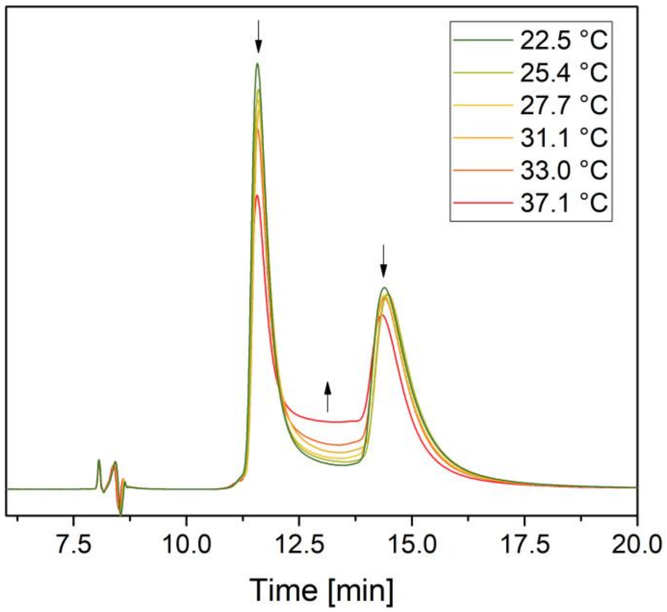

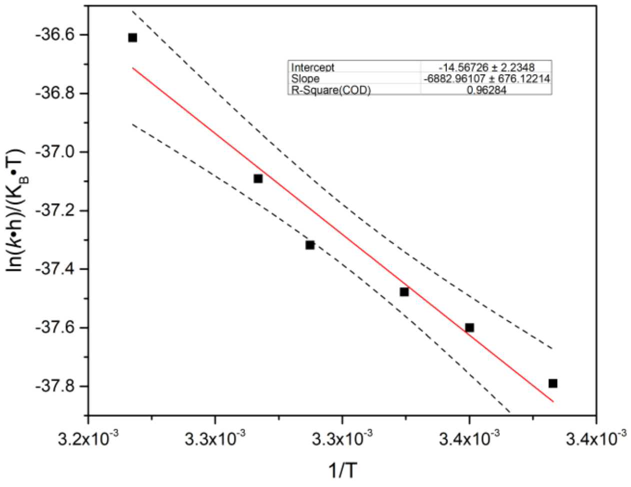

Entry	Temperature (K)	k (s^{-1})
1	295.6	2.384 10^{-4}
2	298.5	2.913 10^{-4}
3	300.8	3.316 10^{-4}
4	304.2	3.937 10^{-4}
5	306.1	4.967 10^{-4}
6	310.8	8.163 10^{-4}

**$\Delta H^{\neq}$**	46.9	kJ×mol^{-1}
**$\Delta S^{\neq}$**	-155.5	J×mol^{-1}×K^{-1}
**$\Delta G^{\neq}_{293\ K}$**	92.7 ± 0.6	kJ×mol^{-1}
**$k_{rac\ 293\ K}$**	1.9 10^{-4} ± 0.5	s^{-1}
**$t_{1/2\ 293\ K}$**	57.7	min

Separation of enantiomers of ***E*-3Pt** was attempted on a Daicel CHIRALPAK IC-3 analytical column. Eluent: Heptane/CH_2Cl_2 70:30, 0.5 ml/min. Column temperature of the following chromatograph is *ca.* 0 °C.

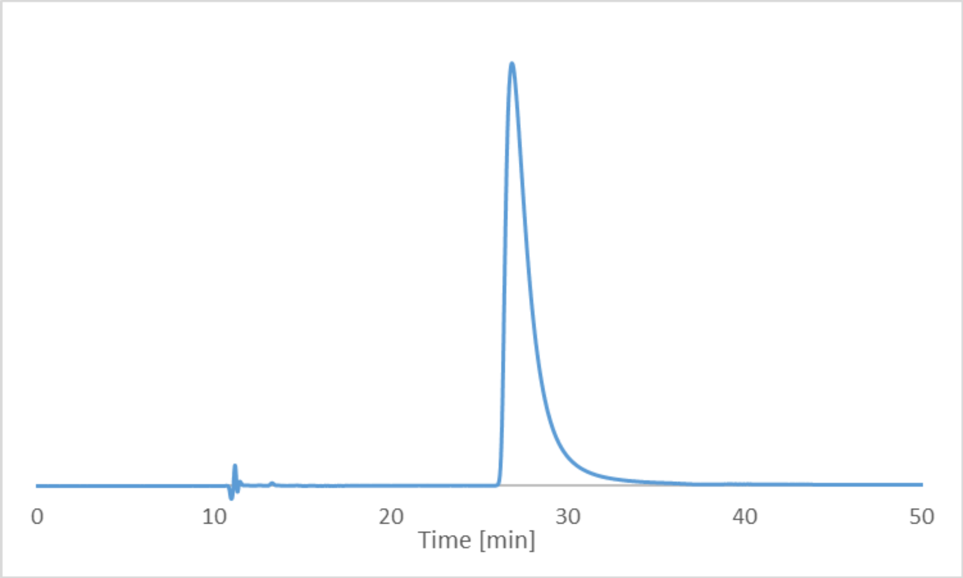

Photochemical isomerization

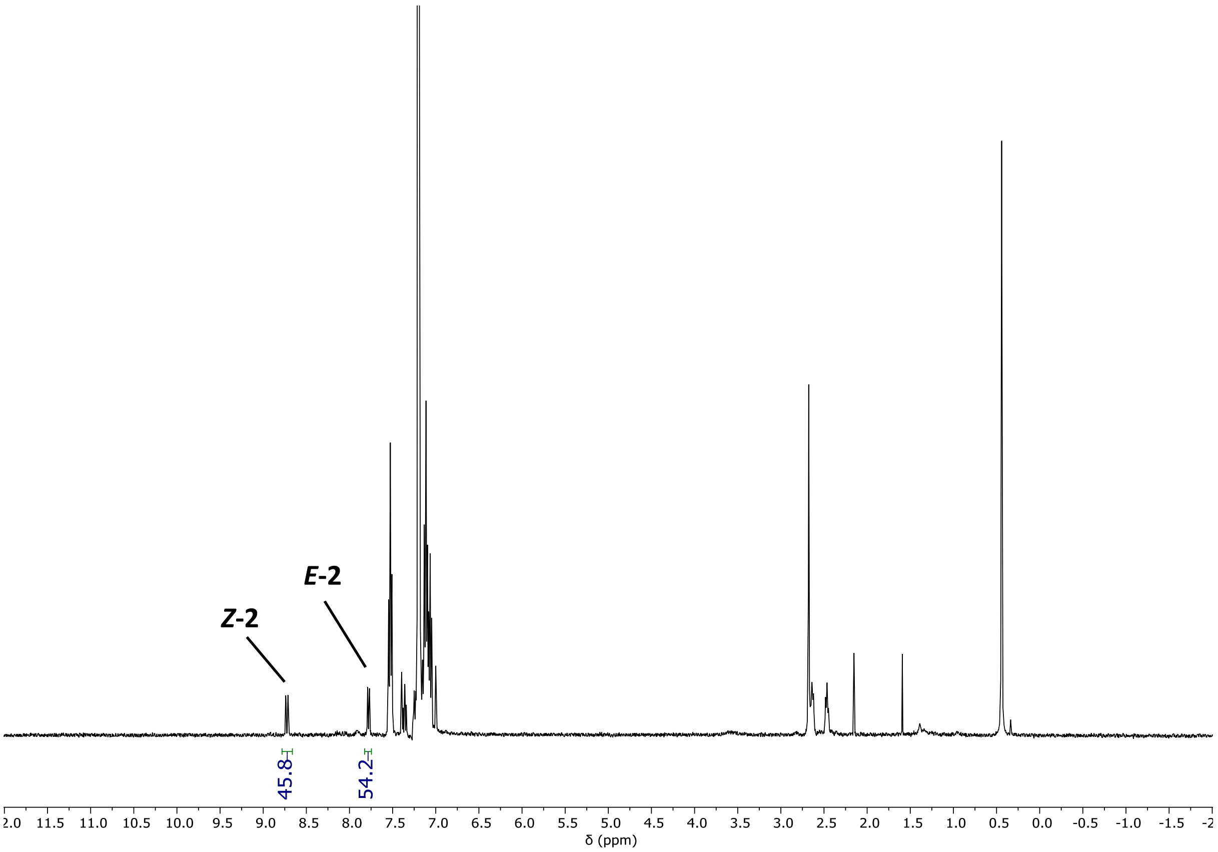

Photostationary state of the E→*Z* isomerization of *E*-2 (365 nm irradiation)

Photoisomerization of Pd complexes E-3 and Z-3.

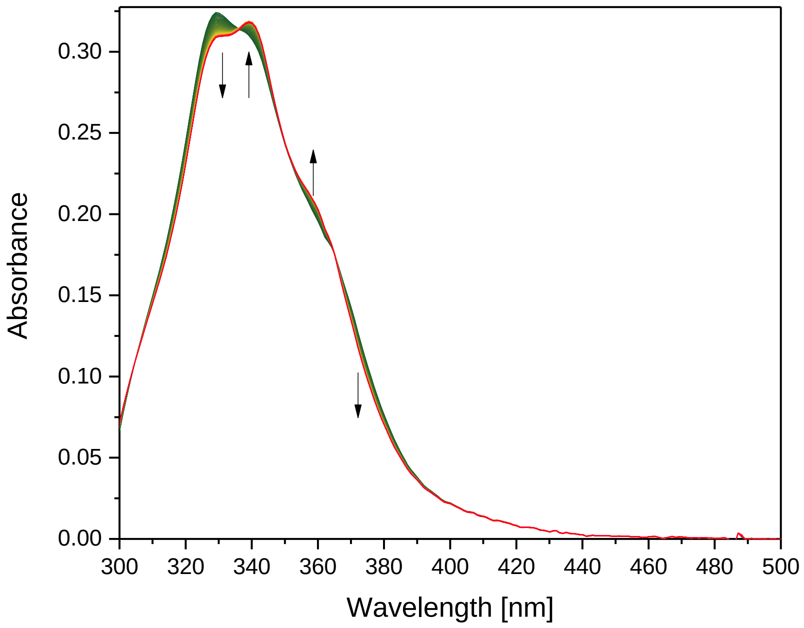

Time-resolved UV-Vis spectrum of the irradiation of *E*-3 in benzene (λ_{irr}=365 nm)

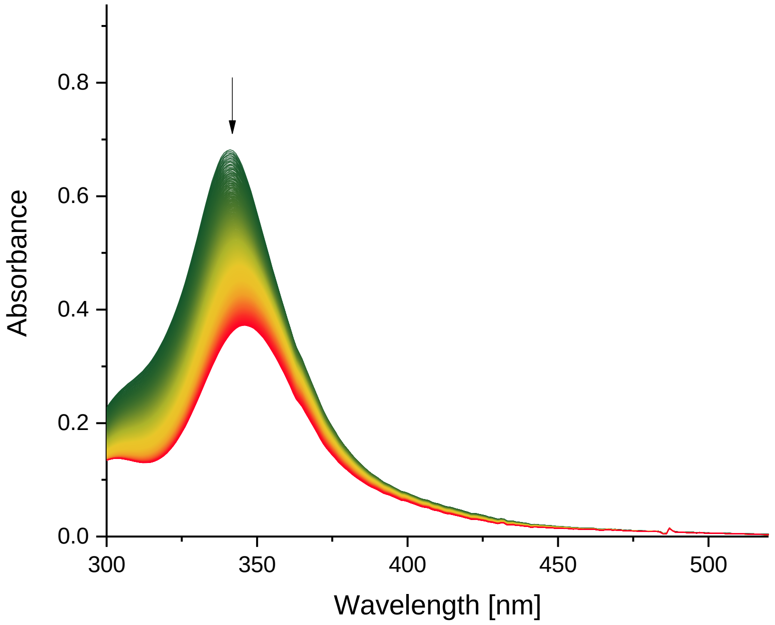

Time-resolved UV-Vis spectrum of the irradiation of *Z*-3 in benzene (λ_{irr}=365 nm). Photobleaching is observed under these reaction conditions.

The photochemical isomerization of ***E*-3** and ***Z-3*** (λ_{irr}=365 nm) was also followed by ^{31}P NMR. No change was observed for ***Z*-3** after 10 minutes of irradiation. For ***E*-3**, two peaks of the same area appeared at 22.0 and 30.4 ppm respectively. We attribute these signals to the isomerization of the palladium center, with the phosphine atoms in a *trans-cis* configuration, as opposed to a *trans-trans* configuration in the dark state of ***E*-3**.

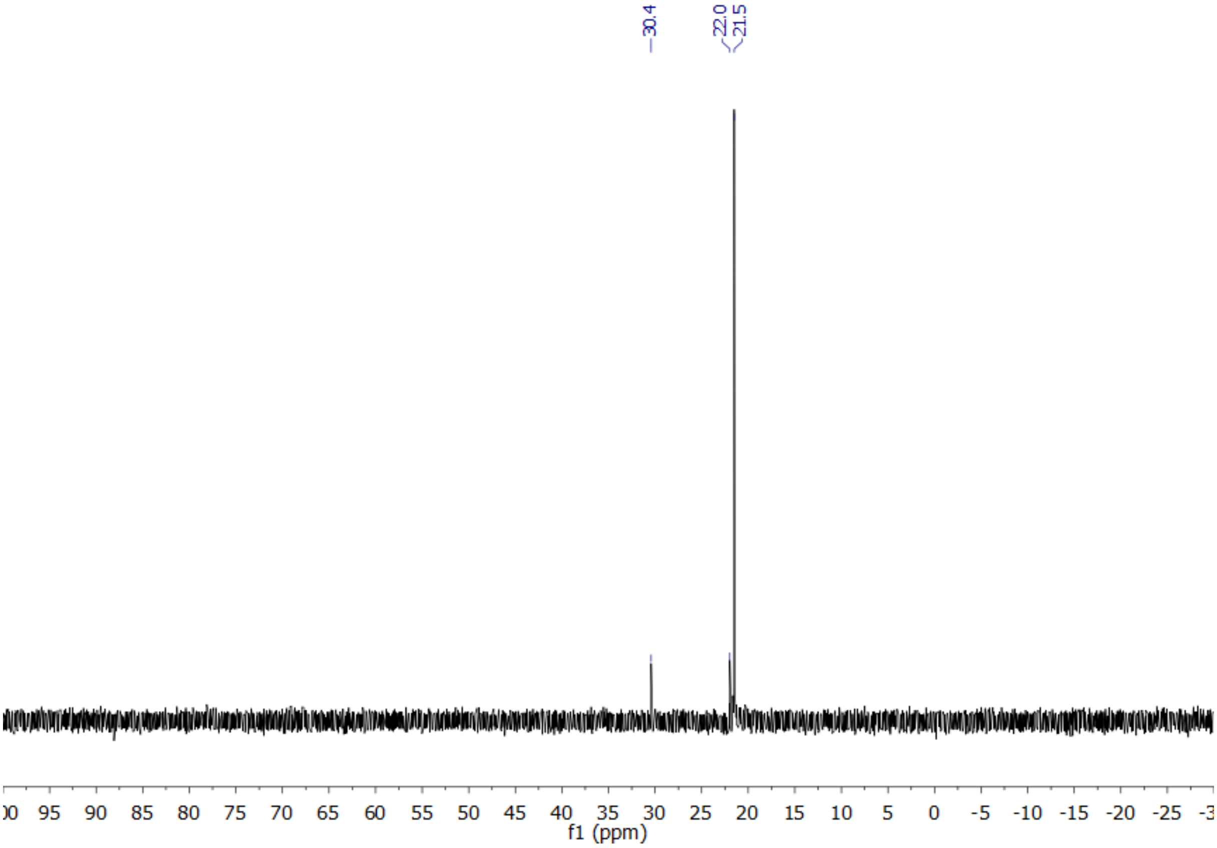

^{31}P NMR of *E*-3 after 10 minutes irradiation (λ_{irr}=365 nm)

Potential Moment of Inertia (PMI) plot

PMI plots represent the shape distribution of the molecules. The three vertices of the triangular plot represent the extremes of molecular geometry. The top left-hand corner represents a linear molecule (diacetylene), the top right-hand corner represents a spherical molecule (adamantane) and the bottom corner represents a disc-like molecule (benzene).[7]

A current trend in medicinal chemistry is to move away from the well populated linear - disc-like axis to generate molecules with more three-dimensionality. The metric that is provided is the mean PMI plot I1 and I2 coordinates of the selected molecules. To generate the PMI coordinates for each molecule, the LLAMA system randomly generates a number of 3D conformers, minimizes their energy and selects the lowest-energy conformer. The system then calculates the moments of inertia in the x, y and z axes. The PMI plot I1 coordinates are calculated by dividing inertia(x) by inertia(z). The I2 coordinates are calculated by dividing inertia(y) by inertia(z).

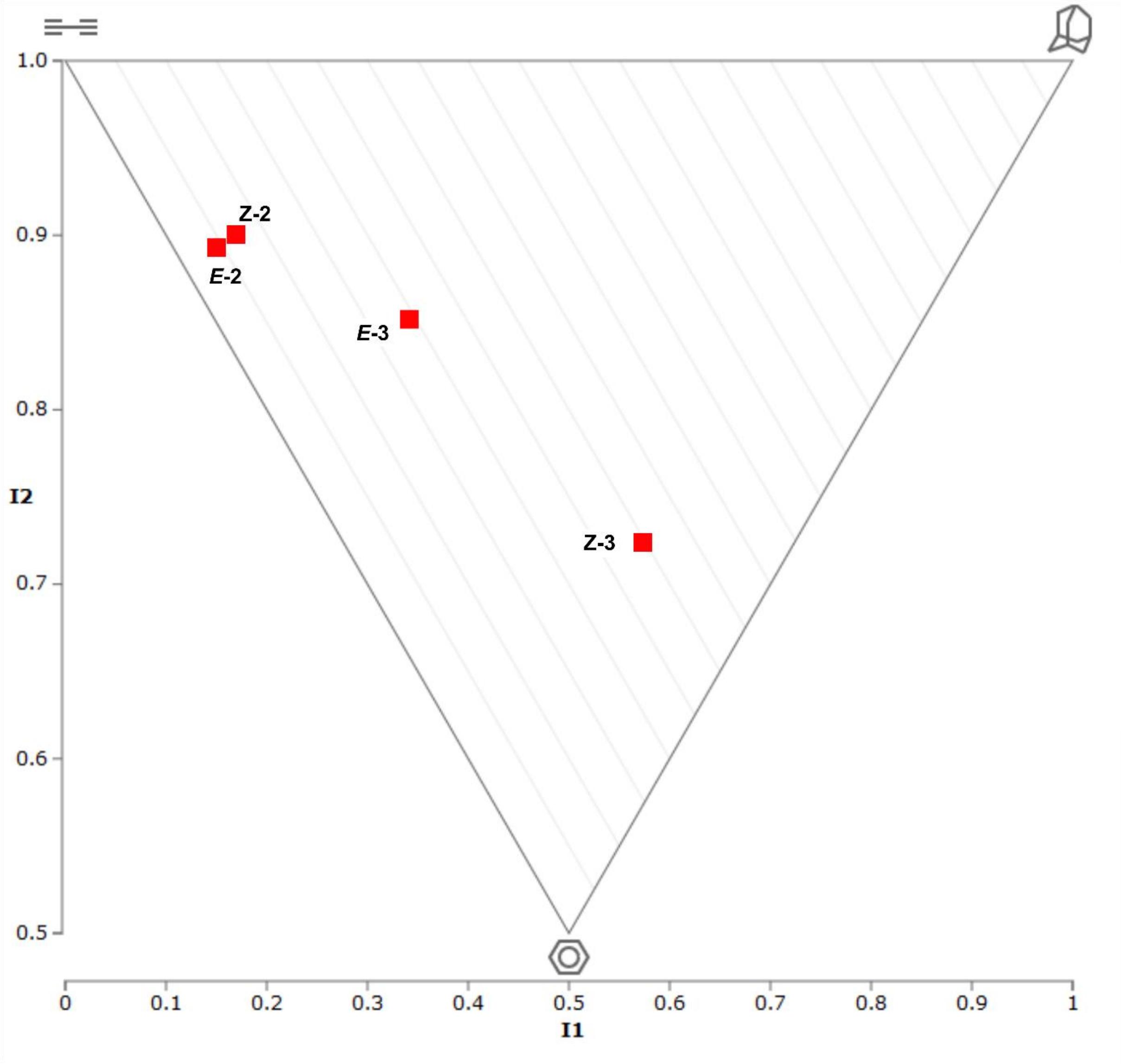

X-Ray structures

Single-crystals were mounted on a cryoloop and placed in the nitrogen stream (100 K) of a Bruker-AXS D8 Venture diffractometer. Data collection and processing was carried out using the Bruker APEX3 software suite.[1] A multi-scan absorption correction was applied, based on the intensities of symmetry-related reflections measured at different angular settings (*SADABS*).[1] The structures were solved using *SHELXT*[2] and refinement was performed using *SHELXL*.[3] The hydrogen atoms were generated by geometrical considerations, constrained by idealized geometries and allowed to ride on their carrier atoms with an isotropic displacement parameter related to the equivalent displacement parameter of their carrier atoms. **Z-3** crystallized as thin needles which were found to be weakly diffracting and its structure could therefore only be solved to a resolution of 0.90 Å. Crystals of **E-3** contained disordered solvent whose contribution was removed using the *PLATON/SQUEEZE* routine.[4] The structure was subsequently refined as an inversion twin for which BASF refined to 0.079. At the end of the refinement, three residual peaks with densities of 0.85–1.7 e/Å^3 which could not be assigned to any resolved structural feature of **E-3** remained within a radius of 1.9 Å around the Pd center. For a more detailed discussion of A- and B-level alerts raised be CheckCIF see below.

Z-3

THETM01_ALERT_3_B The value of sine(theta_max)/wavelength is less than 0.575: Calculated sin(theta_max)/wavelength = 0.5555

The weakly diffracting nature of the crystals led to a two-theta maximum of 118°. The data collection was appropriate for the sample and conditions, and it was possible to unambiguously establish the connectivity and assign the nature of the elements present in the structure.

E-3

PLAT094_ALERT_2_B Ratio of maximum/minimum residual density: 4.63

The absolute values for the highest peak and deepest hole left at the end of the refinement are 1.7 e/Å^3 and –0.36 e/Å^3. The highest residual peak is found in close vicinity to the Pd center. No suspicious elongation of displacement ellipsoids of neighboring atoms are observed.

Nr.	**Z-3**	**E-3**
Name		
Formula	C22.50 H18.50 Cl5.50 P Pd0.50	C42 H34 Cl2 P2 Pd1
Molecular Weight	568.02	777.93
Crystal System	triclinic	orthorhombic
T [K]	100(2)	100(2)
Space Group	P –1	F d d 2
a [Å]	9.6424(4)	35.2248(13)
b [Å]	14.4144(6)	39.0896(14)
c [Å]	18.0591(8)	11.6091(5)
α [°]	76.700(2)	90
β [°]	78.836(2)	90
γ [°]	73.209(2)	90
V [$Å^3$]	2316.67(17)	15984.8(11)
Z	4	16
D_{calc} [$g·cm^{-3}$]	1.629	1.293
F(0 0 0)	1140	6336
h_{min}, h_{max}	–10, 10	–43, 44
k_{min}, k_{max}	–16, 16	–48, 46
l_{min}, l_{max}	–19, 20	–14, 13
μ [mm^{-1}]	9.986	5.929
Crystal Size [mm]	0.20 x 0.05 x 0.03	0.20 x 0.20 x 0.20
Colour, Shape	clear_pale_yellow needle	clear_pale_yellow block
R_{int}	0.0490	0.0408
$θ_{min}$, $θ_{max}$ [°]	4.836, 58.917	3.378, 74.766
Total Reflections (before merge)	19481	41411
Data (I>3 x sigma(I)) [Reflections,Parameters,Restraints]	6578, 532,0	7666, 427, 1
S (=GooF)	1.058	1.057
Min. Residual Density [e/$Å^3$]	–1.026	–0.358
Max. Residual Density [e/Å3]	1.327	1.656
Threshold Expression	I>2sigma(I)	I>2sigma(I)
R_1	0.0668	0.0343
wR_2	0.1413	0.0819

Z-6,6'-bis(diphenylphosphaneyl)-2,2',3,3'-tetrahydro-1,1'-biindenylidene palladium(II) dichloride **Z-3**

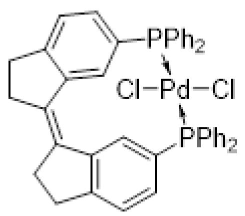

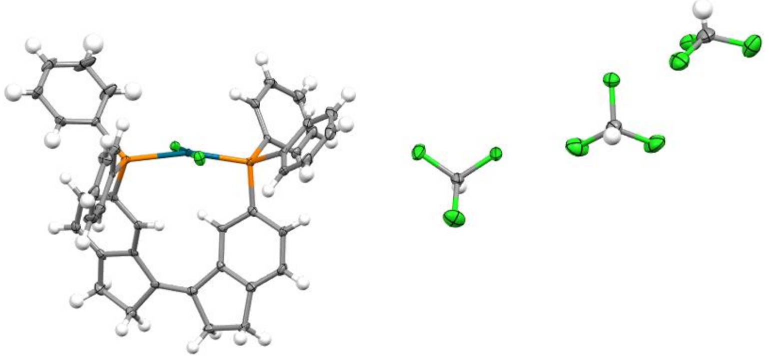

checkCIF/PLATON report

Structure factors have been supplied for datablock(s) LP-18043_xprep_a

THIS REPORT IS FOR GUIDANCE ONLY. IF USED AS PART OF A REVIEW PROCEDURE FOR PUBLICATION, IT SHOULD NOT REPLACE THE EXPERTISE OF AN EXPERIENCED CRYSTALLOGRAPHIC REFEREE.

No syntax errors found. CIF dictionary Interpreting this report

Datablock: LP-18043_xprep_a

Bond precision: C-C = 0.0107 A Wavelength=1.54178

Cell: a=9.6424(4) b=14.4144(6) c=18.0591(8)
alpha=76.700(2) beta=78.836(2) gamma=73.209(2)

Temperature: 100 K

	Calculated	Reported
Volume	2316.67(17)	2316.67(17)
Space group	P -1	P -1
Hall group	-P 1	-P 1
Moiety formula	C42 H34 Cl2 P2 Pd, 3(C H Cl3)	?
Sum formula	C45 H37 Cl11 P2 Pd	C22.50 H18.50 Cl5.50 P Pd0.50
Mr	1136.04	568.02
Dx,g cm-3	1.629	1.629
Z	2	4
Mu (mm-1)	9.986	9.986
F000	1140.0	1140.0
F000'	1151.29	
h,k,lmax	10,16,20	10,16,20
Nref	6655	6578
Tmin,Tmax	0.577,0.741	0.474,0.754
Tmin'	0.129	

Correction method= # Reported T Limits: Tmin=0.474 Tmax=0.754
AbsCorr = MULTI-SCAN

Data completeness= 0.988 Theta(max)= 58.917

R(reflections)= 0.0560(5632) wR2(reflections)= 0.1413(6578)

S = 1.058 Npar= 532

E-3

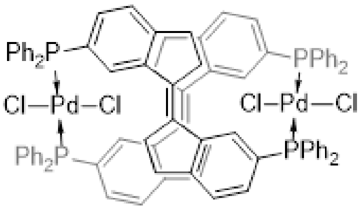

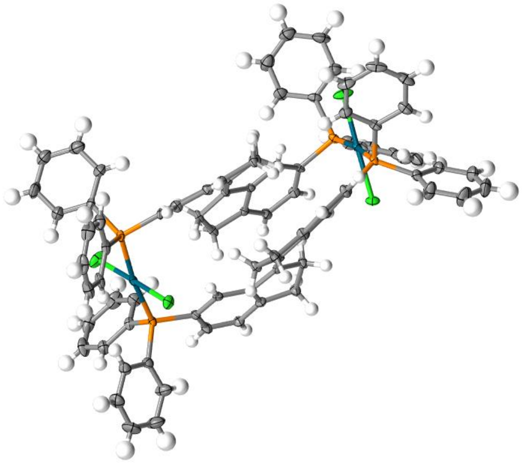

checkCIF/PLATON report

Structure factors have been supplied for datablock(s) lp18058_xprep_a_sq_sq

THIS REPORT IS FOR GUIDANCE ONLY. IF USED AS PART OF A REVIEW PROCEDURE FOR PUBLICATION, IT SHOULD NOT REPLACE THE EXPERTISE OF AN EXPERIENCED CRYSTALLOGRAPHIC REFEREE.

No syntax errors found. CIF dictionary Interpreting this report

Datablock: lp18058_xprep_a_sq_sq

Bond precision:	C-C = 0.0077 A		Wavelength=1.54178
Cell:	a=35.2248(13)	b=39.0896(14)	c=11.6091(5)
	alpha=90	beta=90	gamma=90
Temperature:	100 K		

	Calculated	Reported
Volume	15984.8(11)	15984.8(11)
Space group	F d d 2	F d d 2
Hall group	F 2 -2d	F 2 -2d
Moiety formula	C84 H68 Cl4 P4 Pd2 [+ solvent]	?
Sum formula	C84 H68 Cl4 P4 Pd2 [+ solvent]	C42 H34 Cl2 P2 Pd
Mr	1555.86	777.93
Dx,g cm-3	1.293	1.293
Z	8	16
Mu (mm-1)	5.929	5.929
F000	6336.0	6336.0
F000’	6371.52	
h,k,lmax	44,48,14	44,48,14
Nref	8201[4312]	7666
Tmin,Tmax	0.351,0.306	0.640,0.754
Tmin’	0.266	

Correction method= # Reported T Limits: Tmin=0.640 Tmax=0.754
AbsCorr = MULTI-SCAN

Data completeness= 1.78/0.93 Theta(max)= 74.766

R(reflections)= 0.0327(7411) wR2(reflections)= 0.0819(7666)

S = 1.057 Npar= 427

Computational analysis

All the calculations were carried out using the Gaussian 16 Rev. B.01 1 at the ωB97X-D/def2-TZVP(SDD)//M06L/6-31G*(LANL2DZ) level of theory.[8] All the optimizations were confirmed to be stationary points by the absence of imaginary frequencies.

***E*-3**

Pd	5.0525905902	0.0000038258	0.0000313192
Cl	4.9754551929	-1.211811271	2.0486302484
P	5.0256113429	-2.0288851065	-1.286649065
C	-0.0499991024	-0.6243269251	-2.0644623195
Cl	4.9756711973	1.2118088284	-2.0485791755
P	-5.1505416308	1.9537632523	-1.3031738396
C	1.0854190595	-1.5316205442	-2.1598616154
C	2.4384446207	-1.3193856588	-1.8718159665
H	2.8280455154	-0.3364643856	-1.6340800463
C	3.3276658944	-2.3949287787	-1.838972788
C	2.8775753794	-3.6866990328	-2.1595208594
H	3.5650617281	-4.5310044011	-2.1100340658
C	1.5429433744	-3.8942290773	-2.5131535501
H	1.1994466903	-4.8975587283	-2.7655956942
C	0.6491646964	-2.8285288671	-2.4976628014
C	-0.8244971912	-2.8297610758	-2.773437641
H	-1.3522864114	-3.6355113163	-2.2473493925
H	-1.0125291137	-2.9951748295	-3.8442313817
C	-1.3019926879	-1.4347981239	-2.3302163655
H	-1.9508960371	-0.9688490927	-3.0832592173
H	-1.9213763785	-1.5118428268	-1.4219058012
C	0.0009008157	0.6888356302	-1.7087374998
C	-1.1243461986	1.5759813887	-1.4442533877
C	-2.5027087714	1.3458260174	-1.4963281522
H	-2.9128055422	0.382860625	-1.7971258994
C	-3.3924124931	2.3742904223	-1.1546473555
C	-2.9141719384	3.6114726296	-0.7112590568
H	-3.6131292455	4.3894740012	-0.4084170191
C	-1.5386436068	3.8450759167	-0.6597981852
H	-1.1617371252	4.8116973867	-0.3230122114
C	-0.6546640522	2.8461776265	-1.0418504514
C	0.8391158673	2.9079750218	-1.1104070104
H	1.2840561214	3.2658424774	-0.1713831213
H	1.1597442401	3.6187409204	-1.8864980506
C	1.2694841572	1.4733505385	-1.4472474124
H	1.9648145112	1.4398604373	-2.2963216473
H	1.828876156	1.0414978631	-0.6028658432
C	5.5476992387	-3.5636655192	-0.4579998486
C	4.7258521355	-4.1069102632	0.54051647
H	3.7957944601	-3.606015777	0.8046986483

C	5.1000981792	-5.2715594567	1.1986314986
H	4.4535792642	-5.6844980699	1.9707724967
C	6.3027171884	-5.9003826674	0.8805460113
H	6.5957293886	-6.8110334554	1.4001823364
C	7.1296603247	-5.3601185956	-0.0998208874
H	8.0694293363	-5.8475207732	-0.3531354796
C	6.7565612551	-4.1964189418	-0.7676096987
H	7.4043290677	-3.7862343969	-1.5409301369
C	6.0797916588	-1.9603771705	-2.7689242103
C	5.8058626845	-2.7414395306	-3.8959200352
H	4.9046744456	-3.3536873338	-3.9273259444
C	6.6771241702	-2.7304430452	-4.9810407443
H	6.4546701921	-3.3352226452	-5.8583528802
C	7.8272082267	-1.9452954821	-4.944998903
H	8.5063950798	-1.9365829398	-5.7957687396
C	8.1025695362	-1.1628482146	-3.8258066984
H	8.9922830053	-0.5367089929	-3.8009134777
C	7.2292940249	-1.1641440778	-2.743209325
H	7.4193635005	-0.5279887054	-1.8787883366
C	-5.4299239519	1.7709114518	-3.0964110101
C	-4.5140805957	2.2229146675	-4.0511472323
H	-3.5686124929	2.6620029082	-3.7346961575
C	-4.8062585417	2.1087311086	-5.4086037745
H	-4.0868614239	2.4618041203	-6.1454245336
C	-6.0094008892	1.5437655204	-5.8201252204
H	-6.2336126473	1.4519688713	-6.881450049
C	-6.9234912616	1.0866055519	-4.8719093301
H	-7.8592299085	0.6307319028	-5.189784247
C	-6.633773928	1.1938996123	-3.5178616151
H	-7.3327492086	0.806899522	-2.7763484879
C	-6.1275213899	3.440159474	-0.9073976874
C	-5.9635332389	4.6006793648	-1.6779699063
H	-5.2344502605	4.6142482181	-2.4890399694
C	-6.7322448257	5.7290901466	-1.4172616581
H	-6.5943323228	6.6276588594	-2.0159473763
C	-7.6835855636	5.7034388041	-0.3982743901
H	-8.2890498941	6.5859223738	-0.1984018329
C	-7.8635590969	4.5486184596	0.3570371108
H	-8.6084830303	4.5245276798	1.1500971697
C	-7.0889071395	3.4184226834	0.1069717066
H	-7.2102511957	2.5205946956	0.7092936645
Pd	-5.3691609615	-0.0000331023	0.0000183725
Cl	-5.1341611803	1.3843945582	1.9402742994
Cl	-5.1340465654	-1.3844509146	-1.9402287624
P	5.0255886234	2.0289023885	1.2867028901
C	-0.0500172542	0.6243124805	2.0644860627
P	-5.150540844	-1.953817103	1.3032269136
C	1.0853936984	1.5316147923	2.1598907502
C	2.4384246446	1.3193860603	1.8718663847

H	2.8280357751	0.3364644195	1.6341503303
C	3.3276374618	2.3949359061	1.8390168661
C	2.8775325806	3.6867068812	2.1595406561
H	3.5650113293	4.5310180299	2.1100451632
C	1.5428955084	3.8942311065	2.5131564868
H	1.1993882099	4.8975618376	2.7655795629
C	0.6491253987	2.8285240283	2.4976698354
C	-0.8245400782	2.829748931	2.7734247945
H	-1.3523280953	3.6354925946	2.2473252993
H	-1.0125871522	2.9951669774	3.8442151813
C	-1.3020200179	1.4347800337	2.3302063632
H	-1.950938323	0.9688338771	3.0832384199
H	-1.9213842963	1.5118156517	1.4218817618
C	0.0008938323	-0.6888556255	1.7087830357
C	-1.1243476114	-1.576012032	1.4443102436
C	-2.5027112833	-1.3458608245	1.4963741162
H	-2.912812866	-0.3828885335	1.7971430693
C	-3.3924093824	-2.3743376118	1.1547157579
C	-2.9141620787	-3.6115300027	0.7113635362
H	-3.6131154001	-4.389543384	0.4085424236
C	-1.5386325827	-3.845129208	0.6599141667
H	-1.1617212271	-4.8117581614	0.3231547663
C	-0.6546582025	-2.8462166277	1.0419420767
C	0.8391216825	-2.908005324	1.1105067825
H	1.2840663534	-3.2658888892	0.1714908586
H	1.1597516933	-3.6187545716	1.8866124209
C	1.2694818697	-1.4733711642	1.4473174402
H	1.964807269	-1.4398577987	2.2963949992
H	1.8288780549	-1.0415374126	0.6029290509
C	5.5476571535	3.5636859105	0.4580458867
C	4.7257979723	4.1069218041	-0.540465385
H	3.7957430817	3.6060185756	-0.8046398833
C	5.1000280383	5.2715737603	-1.1985842249
H	4.4534996753	5.6845051009	-1.9707210086
C	6.302642975	5.9004091088	-0.880508147
H	6.595642748	6.8110618566	-1.4001478754
C	7.1295978599	5.3601551557	0.0998542193
H	8.0693641195	5.8475665368	0.3531614614
C	6.7565145696	4.1964528814	0.7676478238
H	7.4042920962	3.7862762443	1.540964278
C	6.0797680696	1.9604252747	2.7689816621
C	5.8058258996	2.7415083802	3.8959609236
H	4.9046299597	3.3537459423	3.9273504535
C	6.6770824831	2.730545007	4.9810853089
H	6.4546178114	3.3353403779	5.8583838145
C	7.8271772394	1.9454114774	4.9450637037
H	8.5063608832	1.9367253555	5.7958363451
C	8.1025546538	1.1629484149	3.8258871912
H	8.9922782054	0.5368230258	3.801007763

C	7.2292833932	1.1642110884	2.7432854685
H	7.419370659	0.5280481969	1.8788743545
C	-5.4299374628	-1.7709392164	3.096459108
C	-4.5140723535	-2.2228601233	4.0512126733
H	-3.5685803005	-2.6619093889	3.7347797269
C	-4.8062599309	-2.1086455583	5.4086646715
H	-4.0868454638	-2.4616548526	6.1454990474
C	-6.0094339791	-1.543731929	5.8201643695
H	-6.2336528445	-1.451910742	6.8814855744
C	-6.9235476707	-1.0866559544	4.8719304132
H	-7.8593126045	-0.6308233855	5.1897869407
C	-6.6338206164	-1.1939803351	3.5178874948
H	-7.3328164568	-0.8070441237	2.7763601992
C	-6.1275063836	-3.4402263194	0.9074674969
C	-5.9635159695	-4.6007290551	1.6780652526
H	-5.2344426779	-4.6142741325	2.4891443827
C	-6.7322121748	-5.7291531151	1.4173696377
H	-6.5942981198	-6.6277083357	2.0160752732
C	-7.6835400372	-5.703532342	0.3983694975
H	-8.2889928696	-6.5860261644	0.198507098
C	-7.8635158478	-4.5487291185	-0.3569677048
H	-8.6084301234	-4.5246620309	-1.1500375984
C	-7.0888790106	-3.4185199999	-0.106915024
H	-7.2102254119	-2.5207052262	-0.7092560067

Energy= -6701.4401247

Zero-point correction= 1.277464 (Hartree/Particle)

Thermal correction to Gibbs Free Energy= 1.145537

Sum of electronic and zero-point Energies= -6700.162661

Sum of electronic and thermal Energies= -6700.076731

Sum of electronic and thermal Enthalpies= -6700.075787

Sum of electronic and thermal Free Energies= -6700.294588

Energy (wB97X-D/def2-TZVP(SDD))= -6704.4511013

***E*-3'**

Pd	5.3407563691	-4.3968104314	-2.2540128141
Cl	5.2939935293	-6.1878998747	-3.8101992534
P	4.6097058961	-2.9293435664	-3.992032942
C	-0.2254310293	-0.5857351366	-3.0849907974
Cl	5.3162232027	-2.5745962723	-0.6909933371
P	-4.9196586888	2.6835082697	-2.4476059022
C	0.7473173529	-1.6666723503	-3.2216155794
C	2.1097101462	-1.6454575752	-3.5628323005
H	2.610090821	-0.7145854911	-3.8105545764
C	2.8311054449	-2.8405403638	-3.607027454
C	2.1975542849	-4.0620538107	-3.3101228387
H	2.7752232289	-4.9881097868	-3.3415632284
C	0.8448480471	-4.0900053665	-2.9931776251
H	0.3584243761	-5.0386958387	-2.7637298462
C	0.1245762717	-2.9027229416	-2.9575706744
C	-1.3252180456	-2.7224337368	-2.6413850764
H	-1.5329915003	-3.0338546201	-1.6060127767
H	-1.9673754429	-3.3463951661	-3.2785848848
C	-1.5799173432	-1.2206206878	-2.8423859013
H	-2.2605215898	-1.055774218	-3.6894909413
H	-2.085129286	-0.7652171441	-1.9768462503
C	0.0068346963	0.7542887696	-3.0829162276
C	-0.9848743922	1.8019143479	-2.8531398527
C	-2.3818060012	1.7618755397	-2.8693795012
H	-2.9257090657	0.8570982904	-3.1348886476
C	-3.1174060755	2.9088948637	-2.5333882486
C	-2.4596116046	4.087445999	-2.166822817
H	-3.0339038447	4.9602536497	-1.8612169518
C	-1.064327411	4.1419907587	-2.1876169751
H	-0.5547468026	5.0667343967	-1.9176465144
C	-0.3362722811	3.0154826681	-2.54169
C	1.1526556797	2.8437370205	-2.593560887
H	1.5818066254	2.9019360163	-1.5812034893
H	1.6497956431	3.6303270452	-3.1750804446
C	1.3548474559	1.4386103461	-3.1881754814
H	1.6738841732	1.50656036	-4.2404973373
H	2.1533778165	0.893064969	-2.6707666967
C	4.6867400042	-3.5610795235	-5.7007340929
C	3.5529662656	-3.6746863321	-6.5096454228
H	2.5716915429	-3.4174305456	-6.1123833884
C	3.6751038599	-4.1186924857	-7.8248152262
H	2.7868660544	-4.2052187954	-8.4482548191
C	4.9247030439	-4.4523905821	-8.3372109144
H	5.0171078305	-4.8028765218	-9.3637122165
C	6.0576211198	-4.346958111	-7.5312853655
H	7.0351305835	-4.6214006928	-7.9231931051
C	5.9399427989	-3.909577859	-6.218648188
H	6.8210732772	-3.8581814713	-5.5794032434

C	5.1885850952	-1.2090496482	-4.1996873831
C	4.6918698669	-0.4439086456	-5.2668189277
H	3.9776610797	-0.8846757263	-5.9632784684
C	5.1086387395	0.8704387643	-5.4429545355
H	4.711747011	1.456651069	-6.2701230291
C	6.0407353857	1.4286707539	-4.5691830596
H	6.3717473011	2.456189388	-4.7108675421
C	6.5554889194	0.6671311946	-3.5246143682
H	7.2907939867	1.0958514247	-2.8463040662
C	6.1333423091	-0.6473758327	-3.3361041118
H	6.5191218354	-1.2366381881	-2.507677524
C	-5.4285045513	2.2544121202	-4.1446226063
C	-4.6216185517	2.5178493692	-5.2556350987
H	-3.6245413713	2.9350656949	-5.1178840407
C	-5.087817547	2.2406025539	-6.5386170517
H	-4.4528550444	2.4460879961	-7.3987533969
C	-6.3581778622	1.7017833413	-6.7194296748
H	-6.7190770808	1.482806826	-7.7229078267
C	-7.1644376952	1.4337797181	-5.6142872758
H	-8.1523927684	0.9982888809	-5.7506244289
C	-6.7012753398	1.7029463149	-4.3326641927
H	-7.3156260646	1.4609661159	-3.4658728453
C	-5.6860056562	4.3185711863	-2.2146481353
C	-5.3777133707	5.3662272121	-3.0938321487
H	-4.6413306938	5.2129852057	-3.883696805
C	-6.0118642146	6.5965972777	-2.9658643114
H	-5.7624456948	7.4074619737	-3.6478808773
C	-6.971061868	6.7853364982	-1.9718075193
H	-7.4700576639	7.7480602282	-1.874778148
C	-7.2941347675	5.7428155193	-1.1083026388
H	-8.0440247655	5.8874160863	-0.3330374071
C	-6.6539620976	4.5116494446	-1.2253645909
H	-6.884673485	3.7004656773	-0.5380820256
Pd	-5.0090181793	1.0037856189	-0.7828429886
Cl	-4.7548899353	2.726065446	0.8627485322
Cl	-5.2379725968	-0.64443007	-2.4869513796
P	5.377308684	-5.966678539	-0.4634870691
C	0.4691004524	-4.1135700084	0.6009274978
P	-4.3323678723	-0.620070019	0.8187855393
C	1.4973699201	-5.1417154522	0.4755121072
C	2.8291730299	-5.0595760658	0.0482130515
H	3.2652640774	-4.124032756	-0.2868369119
C	3.6368935024	-6.2032251795	0.0382064233
C	3.1123716712	-7.4363725735	0.4513323951
H	3.7424882261	-8.3241058414	0.4582475082
C	1.7774241143	-7.5304557469	0.8463691487
H	1.3705134521	-8.4944578607	1.1516412368
C	0.9765954528	-6.3965029506	0.8511793432
C	-0.4662601633	-6.2805076918	1.2418724439

H	-0.6068526394	-6.5428117882	2.2999704594
H	-1.1010983605	-6.9760774546	0.6772258719
C	-0.8261971277	-4.8068509234	0.9733917239
H	-1.5656831054	-4.7252827425	0.1599884366
H	-1.3016964574	-4.343080959	1.8480172476
C	0.6408636941	-2.7732162157	0.4409966155
C	-0.3590781212	-1.7143243322	0.5435885854
C	-1.7464885629	-1.7748698683	0.745228936
H	-2.2452201265	-2.7279121232	0.8903058556
C	-2.5093305424	-0.6022531321	0.7085262525
C	-1.8787598964	0.640404931	0.5158587123
H	-2.4774163754	1.551328576	0.4853021261
C	-0.5005818397	0.7066734584	0.3476736104
H	-0.02368444	1.6744190785	0.1896935503
C	0.2511292466	-0.4606593957	0.3390246392
C	1.720551264	-0.6071975848	0.1059467151
H	2.3035859455	-0.0978012441	0.8858089746
H	2.0245399926	-0.1453041846	-0.8447614352
C	1.9606694022	-2.1215361471	0.1012367401
H	2.31837406	-2.4505505465	-0.8842649198
H	2.7543627498	-2.4128824872	0.8010571425
C	6.2408444483	-5.4054450162	1.0381327065
C	7.3893281156	-4.6178423462	0.9085433456
H	7.6933232996	-4.2745492859	-0.0799160285
C	8.1179218947	-4.2552575372	2.0366668506
H	9.0084737635	-3.6390507412	1.9292182284
C	7.6972677203	-4.6644374096	3.3001821559
H	8.2635016963	-4.3728049291	4.1831137197
C	6.5474086941	-5.4389977421	3.4355892452
H	6.2133602284	-5.7544558932	4.4224433454
C	5.8207508534	-5.8117180266	2.3087279233
H	4.9220535325	-6.419462187	2.4146649895
C	6.0462353684	-7.635999065	-0.7428272119
C	5.367292847	-8.5293444612	-1.585643372
H	4.4354184916	-8.2251998137	-2.0563335486
C	5.8847442082	-9.7966977041	-1.8255169168
H	5.3464762948	-10.4806906549	-2.4787906481
C	7.0908006527	-10.184191855	-1.245309231
H	7.4964498414	-11.1754661647	-1.4402067392
C	7.7765169729	-9.2998077431	-0.4178782731
H	8.7184921084	-9.595918977	0.0400895881
C	7.2588130755	-8.032691269	-0.1652613177
H	7.7984592276	-7.3525615459	0.4916118055
C	-4.744458281	-0.2658759314	2.5543819412
C	-3.8907831929	-0.6570885907	3.5909244099
H	-2.9330254703	-1.1229525201	3.3579211099
C	-4.2628941036	-0.4499610709	4.9157843352
H	-3.5927468663	-0.7512987147	5.718925346
C	-5.487988662	0.1436747906	5.2115732244

H	-5.7764114676	0.3070682012	6.2485559153
C	-6.3397748407	0.5375765363	4.1818329965
H	-7.2908766991	1.0143060809	4.4108489834
C	-5.967982917	0.3402685261	2.856104736
H	-6.6126541695	0.6763788386	2.044705491
C	-4.7898065305	-2.3717322288	0.6137230715
C	-4.3465930111	-3.0647155939	-0.5234004965
H	-3.7607651144	-2.5408954049	-1.2750870967
C	-4.6549219393	-4.4088655717	-0.6927893566
H	-4.2962879364	-4.9333478289	-1.5775846089
C	-5.4250324045	-5.0761290438	0.2584842071
H	-5.6699447015	-6.1282449989	0.1231405539
C	-5.8844957665	-4.3915667441	1.3798753623
H	-6.487967114	-4.9057218556	2.1257042973
C	-5.5686716614	-3.04698967	1.5607384881
H	-5.921243494	-2.5255278675	2.449120871

Energy= -6701.4283518

Zero-point correction= 1.277704 (Hartree/Particle)

Thermal correction to Gibbs Free Energy= 1.144826

Sum of electronic and zero-point Energies= -6700.150648

Sum of electronic and thermal Energies= -6700.064659

Sum of electronic and thermal Enthalpies= -6700.063715

Sum of electronic and thermal Free Energies= -6700.283526

Energy= -6704.4364923

References

[1] Bruker, (2016). APEX3 (V2016.1-0), SAINT (Version 8.37A) and SADABS (Version 2014/5). Bruker AXS Inc., Madison, Wisconsin, USA.

[2] G. M. Sheldrick, *Acta Cryst. A*, **2008**, *A64*, 112–122.

[3] G. M. Sheldrick, *Acta Cryst. A*, **2015**, *71*, 3–8.

[4] A. L. Spek, *Acta Cryst. C,* **2015**, *71*, 9–18.

[5] S. J. Wezenberg, and B. L. Feringa, *Org. Lett.*, **2017**, 19, 324–327

[6] O. Kühl, *Phosphorus-31 NMR Spectroscopy: A Concise Introduction for the Synthetic Organic and Organometallic Chemist*, Springer-Verlag, Heidelberg, **2008**.

[7] I. Colomer, C. J. Empson, P. Craven, Z. Owen, R. G. Doveston, I. Churcher, S. P. Marsden, A. Nelson, *Chem. Commun.*, **2016**, 52, 7209-7212

[8] Gaussian 16, Revision B.02. Gaussian, Inc., Wallingford CT 2016.
